# Model‐free portfolio theory: A rough path approach

**DOI:** 10.1111/mafi.12376

**Published:** 2023-01-24

**Authors:** Andrew L. Allan, Christa Cuchiero, Chong Liu, David J. Prömel

**Affiliations:** ^1^ Durham University Durham UK; ^2^ University of Vienna Vienna Austria; ^3^ ShanghaiTech University Shanghai China; ^4^ University of Mannheim Mannheim Germany

**Keywords:** Cover's universal portfolio, log‐optimal portfolio, model uncertainty, pathwise integration, rough path, stochastic portfolio theory

## Abstract

Based on a rough path foundation, we develop a model‐free approach to stochastic portfolio theory (SPT). Our approach allows to handle significantly more general portfolios compared to previous model‐free approaches based on Föllmer integration. Without the assumption of any underlying probabilistic model, we prove a pathwise formula for the relative wealth process, which reduces in the special case of functionally generated portfolios to a pathwise version of the so‐called master formula of classical SPT. We show that the appropriately scaled asymptotic growth rate of a far reaching generalization of Cover's universal portfolio based on controlled paths coincides with that of the best retrospectively chosen portfolio within this class. We provide several novel results concerning rough integration, and highlight the advantages of the rough path approach by showing that (nonfunctionally generated) log‐optimal portfolios in an ergodic Itô diffusion setting have the same asymptotic growth rate as Cover's universal portfolio and the best retrospectively chosen one.

## 1 INTRODUCTION

Classical approaches to portfolio theory, going back to the seminal work of Markowitz (1959) (see also the early work of de Finetti (1940)), are essentially based on simplistic probabilistic models for the asset returns or prices. As a first step, classical portfolio selection, thus, requires to build and statistically estimate a probabilistic model of the future asset returns. The second step is usually to find an “optimal” portfolio with respect to the now fixed model. However, it is well known that the obtained optimal portfolios and their performance are highly sensitive to model misspecifications and estimation errors; see, for example, Chopra and Ziemba (1993); DeMiguel et al. (2007).

In order to account for model misspecification and model risk, the concept of model ambiguity, also known as Knightian uncertainty, has gained increasing importance in portfolio theory; see, for example, Pflug and Wozabal (2007); Guidolin and Rinaldi (2013). Here the rationale is to accomplish the portfolio selection with respect to a pool of probabilistic models, rather than a specific one. This has been pushed further by adopting completely *model‐free* (or pathwise) approaches, where the trajectories of the asset prices are assumed to be deterministic functions of time. That is, no statistical properties of the asset returns or prices are postulated; see, for example, Pal and Wong (2016); Schied et al. (2018); Cuchiero et al. (2019). In portfolio theory, there are two major approaches, which provide such model‐free ways of determining “optimal” portfolios: universal and stochastic portfolio theory (SPT).

The objective of universal portfolio theory is to find general preference‐free well‐performing investment strategies without referring to a probabilistic setting; see Li and Hoi (2014) for a survey. This theory was initiated by Cover (1991), who showed that a properly chosen “universal” portfolio has the same asymptotic growth rate as the best retrospectively chosen (constantly rebalanced) portfolio in a discrete‐time setting. Here, the word “universal” indicates the model‐free nature of the constructed portfolio.

SPT, initiated by Fernholz (1999, 2001), constitutes a descriptive theory aiming to construct and analyze portfolios using only properties of observable market quantities; see Fernholz (2002); Karatzas and Fernholz (2009) for detailed introductions. While classical SPT still relies on an underlying probabilistic model, its descriptive nature leads to essentially model‐free constructions of “optimal” portfolios.

A model‐free treatment of universal and SPT in *continuous‐time* was recently introduced in Schied et al. (2018); Cuchiero et al. (2019), clarifying the model‐free nature of these theories. So far, this analysis has been limited to so‐called (generalized) *functionally generated portfolios*, compare Fernholz (1999); Strong (2014); Schied et al. (2018). These are investment strategies based on logarithmic gradients of so‐called portfolio generating functions. This limitation is due to the fact that the corresponding portfolio wealth processes can be defined in a purely pathwise manner only for gradient‐type strategies, namely, via Föllmer's probability‐free notion of Itô integration; see Föllmer's pioneering work (Föllmer, 1981) and its extensions (Cont and Fournié, 2010; Cont and Perkowski, 2019; Chiu and Cont, 2022a, 2022b). Even though these limitations do not occur in discrete time, optimal portfolio selection approaches based on functionally generated portfolios have also gained attention in discrete time setups; see, for example, Campbell and Wong (2022). Another strand of research is robust maximization of asymptotic growth within a pool of Markovian models as pursued in Kardaras and Robertson (2012, 2021); Itkin and Larsson (2022). While these approaches clearly account for model uncertainty, a probabilistic structure still enters via a Markovian volatility matrix and an invariant measure for the market weights process. In a similar direction goes the construction of optimal arbitrages under model uncertainty as pioneered in Fernholz and Karatzas (2011).

The main goal of the present article is to develop an entirely model‐free portfolio theory in continuous‐time, in the spirit of stochastic and universal portfolio theory, which allows one to work with a significantly larger class of investment strategies and portfolios. For this purpose, we rely on the pathwise (rough) integration offered by rough path theory—as exhibited in, for example, Lyons and Qian (2002); Lyons et al. (2007); Friz and Victoir (2010); Friz and Hairer (2020)—and assume that the (deterministic) price trajectories on the underlying financial market satisfy the so‐called Property (RIE), as introduced in Perkowski and Prömel (2016); see Section 2.2. While Property (RIE) does not require any probabilistic structure, it is satisfied, for instance, by the sample paths of semimartingale models fulfilling the condition of “no unbounded profit with bounded risk” and, furthermore, it ensures that rough integrals are given as limits of suitable Riemann sums. This is essential in view of the financial interpretation of the integral as the wealth process associated to a given portfolio.

In the spirit of SPT, we are interested in the relative performance of the wealth processes, where the word “relative” may be interpreted as “in comparison with the market portfolio.” In other words, given *d* assets with associated price process $S={\left(S_{t}^{1},\text{…},S_{t}^{d}\right)}_{t\in [0,\infty )}$ satisfying Property (RIE), we choose the total market capitalization $S^{1}+\cdots +S^{d}$ as numéraire, so that the primary assets are the market weights $\mu ={\left(\mu_{t}^{1},\text{…},\mu_{t}^{d}\right)}_{t\in [0,\infty )}$, given by

$$\mu_{t}^{i}:=\frac{S_{t}^{i}}{S_{t}^{1}+\cdots +S_{t}^{d}},\;i=1,\cdots ,d,$$

which take values in the open unit simplex $\Delta_{+}^{d}$. The main contributions of the present work may be summarized by the following.

- In Proposition 3.9, we establish a pathwise formula for the relative wealth process associated to portfolios belonging to the space of controlled paths, as introduced in Definition 2.3 below. This includes functionally generated portfolios commonly considered in SPT—as for instance in Strong (2014); Schied and Voloshchenko (2016); Karatzas and Ruf (2017); Ruf and Xie (2019); Karatzas and Kim (2020)—as well as the class, which we refer to as *functionally controlled portfolios*, which are portfolios of the form
(1) $${\left(\pi_{t}^{F}\right)}^{i}=\mu_{t}^{i}\left(F^{i}\left(\mu_{t}\right)+1-\sum_{j=1}^{d}\mu_{t}^{j}F^{j}\left(\mu_{t}\right)\right),$$
  for some $F\in C^{2}\left({\bar{\Delta }}_{+}^{d};R^{d}\right)$. Here, ${\left(\pi^{F}\right)}^{i}$ denotes the proportion of the current wealth invested in asset i=1,⋯,d. In the case of functionally generated portfolios, that is, when *F* is the logarithmic gradient of some real‐valued function, we also derive in Theorem 3.11 a purely pathwise version of the classical master formula of SPT, compare Fernholz (2002); Strong (2014).
- We introduce Cover's universal portfolio defined via a mixture portfolio based on the notion of controlled paths, and show that its appropriately scaled logarithmic relative wealth process converges in the long‐run to that of the best retrospectively chosen portfolio; see Theorems 4.9 and 4.12. This extends the results of Cuchiero et al. (2019) to a considerably larger class of investment strategies.
- In Section 5, we introduce a probabilistic setup where the dynamics of the market weights are described by a stochastic differential equation (SDE) driven by Brownian motion. Using the law of large numbers for the increments of the Itô‐rough path lift of Brownian motion, this setting allows to replace the scaling function of Theorem 4.12 by 1/T. For this class of models, we can thus prove that the asymptotic growth rates of Cover's universal portfolio and the best retrospectively chosen one are the same (see Theorem 5.4(ii)). We also compare these two portfolios with the log‐optimal one assuming additionally that the SDE for the market weights is ergodic. In this case, the corresponding growth rates are all asymptotically equivalent, as shown in Theorem 5.4(iii). This is analogous to the result in Cuchiero et al. (2019), however, now proved for the significantly larger class of functionally controlled portfolios.
- We develop novel results in the theory of rough paths to allow for the pathwise treatment of portfolio theory. In particular, these results include an extension of Perkowski and Prömel (2016, Theorem 4.19), stating that the rough integral can be represented as a limit of left‐point Riemann sums—see Theorem 2.12—and the associativity of rough integration, exhibited in Section A.2.

**FIGURE 1:**
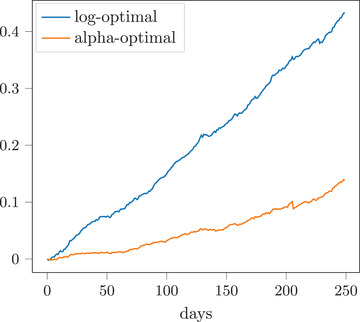
Expected utility of the log‐optimal versus the alpha‐optimal portfolio over time [Colour figure can be viewed at wileyonlinelibrary.com]

One important motivation for our work comes from classical considerations of the log‐optimal portfolio in ergodic Itô diffusion models for the market weights process. Indeed, this is one prominent example of an “optimal” portfolio that does not belong, in general, to the class of (generalized) functionally generated portfolios, but is still a functionally controlled portfolio of the form (1); see Section 5.2. As illustrated numerically in Figure 1, the log‐optimal portfolio (an example of a functionally controlled portfolio) might significantly outperform a corresponding “best” functionally generated portfolio. Indeed, the blue line illustrates the expected utility of the log‐optimal portfolio over time, whereas the orange line depicts that of a certain best functionally generated portfolio. For the details of this example, we refer to Section 5.3.

This indicates that going beyond functionally generated portfolios can have a substantial benefit. This holds true in particular for Cover's universal portfolio when defined as a mixture of portfolios of the form (1), since in ergodic market models, it asymptotically achieves the growth rate of the log‐optimal portfolio (see Theorem 5.4). Note that, due to the rough path approach, both the relative wealth processes obtained by investing according to the log‐optimal portfolio and according to the universal portfolio make sense for every individual price trajectory. This also gives a theoretical justification for learning a (nonfunctionally generated) log‐optimal portfolio from the observations of a single price path.

**Outline**: In Section 2, we provide an overview of the essential concepts of rough paths and rough integration relevant for our financial application. In Section 3, we introduce the pathwise description of the underlying financial market and study the growth of wealth processes relative to that of the market portfolio, which leads us to a pathwise master formula analogous to that of classical SPT. Section 4 is dedicated to Cover's universal portfolio and to proving that its appropriately scaled asymptotic growth rate is equal to that of the best retrospectively chosen portfolio. In Section 5, we introduce a probabilistic setup and show under an ergodicity assumption that the asymptotic growth rate coincides for Cover's universal portfolio, the best retrospectively chosen one and the log‐optimal one. In this setting, we also compare the wealth processes of functionally controlled portfolios and functionally generated ones, illustrating their performance by means of a concrete numerical example. Appendices A and B collect findings concerning rough path theory and rough integration needed to establish the aforementioned results.

## 2 ROUGH INTEGRATION FOR FINANCIAL APPLICATIONS

In this section, we provide the essential concepts from rough path theory for our applications in model‐free portfolio theory. Additional results regarding rough integration are developed in the appendices. For more detailed introductions to rough path theory, we refer to the books (Lyons and Qian, 2002; Lyons et al., 2007; Friz and Victoir, 2010; Friz and Hairer, 2020). Let us begin by introducing some basic notation commonly used in the theory of rough paths.

### 2.1 Basic notation

Let $\left(R^{d},|\;\cdot \;|\right)$ be standard Euclidean space and let A⊗B denote the tensor product of two vectors $A,B\in R^{d}$, that is, the d×d‐matrix with (i,j)‐component given by ${\left[A⊗B\right]}^{ij}=A^{i}B^{j}$ for 1≤i,j≤d. The space of continuous paths $S:\left[0,T\right]\to R^{d}$ is given by $C(\left[0,T\right];R^{d})$, and ${∥S∥}_{\infty ,[0,T]}$ denotes the supremum norm of *S* over the interval [0, *T*]. For the increment of a path $S:\left[0,T\right]\to R^{d}$, we use the standard shorthand notation

$$S_{s,t}:=S_{t}-S_{s},\;\text{for}\;\left(s,t\right)\in \Delta_{\left[0,T\right]}:=\{\left(u,v\right)\in {\left[0,T\right]}^{2}:u\leq v\}.$$

For any partition $P=\{0=t_{0}<t_{1}<\cdots <t_{N}=T\}$ of an interval [0, *T*], we denote the mesh size of P by $\left|P|:=\max\{|t_{k+1}-t_{k}|:k=0,1,\cdots ,N-1\right\}$. A *control function* is defined as a function $c:\Delta_{\left[0,T\right]}\to \left[0,\infty \right)$, which is superadditive, in the sense that c(s,u)+c(u,t)≤c(s,t) for all 0≤s≤u≤t≤T. For p∈[1,∞), the *p*‐variation of a path $S\in C(\left[0,T\right];R^{d})$ over the interval [s,t] is defined by

$${∥S∥}_{p,[s,t]}:=\underset{P\subset [s,t]}{\sup}{\left(\sum_{[u,v]\in P}{\left|S_{u,v}\right|}^{p}\right)}^{\frac{1}{p}},$$

where the supremum is taken over all finite partitions P of the interval [s,t], and we use the abbreviation ${∥S∥}_{p}:={∥S∥}_{p,[0,T]}$. We say that *S* has finite *p*‐variation if ${∥S∥}_{p}<\infty$, and we denote the space of continuous paths with finite *p*‐variation by $C^{p\text{-var}}\left(\left[0,T\right];R^{d}\right)$. Note that *S* having finite *p*‐variation is equivalent to the existence of a control function *c* such that $|S_{s,t}{|}^{p}\leq c\left(s,t\right)$ for all $\left(s,t\right)\in \Delta_{\left[0,T\right]}$. (For instance, one can take $c\left(s,t\right)={∥S∥}_{p,[s,t]}^{p}$.) Moreover, for a two‐parameter function $S:\Delta_{\left[0,T\right]}\to R^{d\times d}$, we introduce the corresponding notion of *p*‐variation by

$${∥S∥}_{p,[s,t]}:=\underset{P\subset [s,t]}{\sup}{\left(\sum_{[u,v]\in P}{\left|S_{u,v}\right|}^{p}\right)}^{\frac{1}{p}},$$

for p∈[1,∞).

Given a k∈N and a domain $A\subseteq R^{d}$, we will write $f\in C^{k}\left(A;R^{d}\right)$, or sometimes simply $f\in C^{k}$, to indicate that a function *f* defined on *A* with values in $R^{d}$ is *k*‐times continuously differentiable (seen as restriction of $C^{k}$‐functions on $R^{d}$ if *A* is closed), and we will make use of the associated norm

$${∥f∥}_{C^{k}}:=\max_{0\leq n\leq k}{∥D^{n}f∥}_{\infty },$$

where $D^{n}f$ denotes the *n*th‐order derivative of *f*, and ${∥\;\cdot \;∥}_{\infty }$ denotes the supremum norm.

For a k∈N and γ∈(0,1], we will write $f\in C^{k+\gamma }\left(A;R^{d}\right)$, or just $f\in C^{k+\gamma }$, to mean that a function *f* defined on *A* is *k*‐times continuously differentiable (in the Fréchet sense), and that its *k*‐order derivative $D^{k}f$ is locally γ‐Hölder continuous. In this case, we use the norm

$${∥f∥}_{C^{k+\gamma }}:=\max_{0\leq n\leq k}∥D^{n}{f∥}_{\infty }+{∥D^{k}f∥}_{\gamma \text{-H}\ddot{o}l},$$

where ${∥\;\cdot \;∥}_{\gamma \text{-H}\ddot{o}l}$ denotes the γ‐Hölder norm.

Finally, given two vector spaces U,V, we write L(U;V) for the space of linear maps from *U* to *V*.

Let (E,∥·∥) be a normed space and let f,g:E→R be two functions. We shall write f≲g or f≤Cg to mean that there exists a constant C>0 such that f(x)≤Cg(x) for all x∈E. Note that the value of such a constant may change from line to line, and that the constants may depend on the normed space, for example, through its dimension or regularity parameters.

### 2.2 Rough path theory and Property (RIE)

Let us briefly recall the fundamental definitions of a rough path and of a controlled path, which allow to set up rough integration.

Definition 2.1 For p∈(2,3), a *p* *‐rough path* is defined as a pair S=(S,S), consisting of a continuous path $S:\left[0,T\right]\to R^{d}$ and a continuous two‐parameter function $S:\Delta_{\left[0,T\right]}\to R^{d\times d}$, such that ${∥S∥}_{p}<\infty$, ${∥S∥}_{p/2}<\infty$, and Chen's relation

(2) $$S_{s,t}=S_{s,u}+S_{u,t}+S_{s,u}⊗S_{u,t}$$

holds for all 0≤s≤u≤t≤T.

Remark 2.2 The success of rough path theory in probability theory is based on the observation that sample paths of many important stochastic processes such as Brownian motion, semimartingales, and Markov processes can be enhanced to a rough path, by defining the “enhancement” S via stochastic integration; see, for example, Friz and Victoir (2010, Part III).

Definition 2.3 Let p∈(2,3) and q≥p be such that 2/p+1/q>1, and let r>1 be such that 1/r=1/p+1/q. Let $S\in C^{p\text{-var}}\left(\left[0,T\right];R^{d}\right)$, $F:\left[0,T\right]\to R^{d}$ and $F^{\prime }:\left[0,T\right]\to L\left(R^{d};R^{d}\right)$ be continuous paths. The pair $\left(F,F^{\prime }\right)$ is called a *controlled path* with respect to *S* (or an *S* *‐controlled path*), if the *Gubinelli derivative* $F^{\prime }$ has finite *q*‐variation, and the *remainder* $R^{F}$ has finite *r*‐variation, where $R^{F}:\Delta_{\left[0,T\right]}\to R^{d}$ is defined implicitly by the relation

$$F_{s,t}=F_{s}^{\prime }S_{s,t}+R_{s,t}^{F}\;\text{for}\;\left(s,t\right)\in \Delta_{\left[0,T\right]}.$$

We denote the space of controlled paths with respect to *S* by $V_{S}^{q}=V_{S}^{q}\left(\left[0,T\right];R^{d}\right)$, which becomes a Banach space when equipped with the norm

$$∥F,F^{\prime }{∥}_{V_{S}^{q},\left[0,T\right]}:=|F_{0}\left|+\right|F_{0}^{\prime }|+∥F^{\prime }{∥}_{q,[0,T]}+{∥R^{F}∥}_{r,[0,T]}.$$

Example 2.4 For a path $S\in C^{p\text{-var}}\left(\left[0,T\right];R^{d}\right)$ with p∈(2,3), the prototypical example of a controlled path is $\left(f\left(S\right),Df\left(S\right)\right)\in V_{S}^{q}$ for any $f\in C^{1+\varepsilon }$ with ε∈(p−2,1] and q=p/ε. Examples of more general controlled paths are discussed in Remark 3.5 and Section 4.1 in the context of universal portfolios.

Based on the above definitions, one can establish the existence of the rough integral of a controlled path $\left(F,F^{\prime }\right)$ with respect to a *p*‐rough path S. See Friz and Hairer (2020) for the corresponding theory presented in terms of Hölder regularity. The following formulation of rough integration in the language of *p*‐variation can be found in, for example, Perkowski and Prömel (2016, Theorem 4.9).

Theorem 2.5 (Rough integration) Let p∈(2,3) and q≥p be such that 2/p+1/q>1, and let r>1 be such that 1/r=1/p+1/q. Let S=(S,S) be a *p*‐rough path and let $\left(F,F^{\prime }\right)\in V_{S}^{q}$ be a controlled path with remainder $R^{F}$. Then the limit

(3) $$\int_{0}^{T}F_{u}\;dS_{u}:=\lim_{|P|\to 0}\sum_{[s,t]\in P}F_{s}S_{s,t}+F_{s}^{\prime }S_{s,t}$$

exists along every sequence of partitions P of the interval [0, *T*] with mesh size |P| tending to zero, and takes values in R. We call this limit the *rough integral* of $\left(F,F^{\prime }\right)$ against S. Here, the product $F_{s}S_{s,t}$ is understood as the Euclidean inner product, and the product $F_{s}^{\prime }S_{s,t}$ also takes values in R since the derivative $F^{\prime }$ takes values in $L\left(R^{d};R^{d}\right)≅L\left(R^{d\times d};R\right)$. Moreover, we have the estimate

(4) $$|\int_{s}^{t}F_{u}\;dS_{u}-F_{s}S_{s,t}-F_{s}^{\prime }S_{s,t}|\leq C\left(∥R^{F}{∥}_{r,[s,t]}{∥S∥}_{p,[s,t]}+∥F^{\prime }{∥}_{q,[s,t]}{∥S∥}_{\frac{p}{2},\left[s,t\right]}\right),$$

where the constant *C* depends only on p,q, and *r*.

In Theorem 2.5, we defined the rough integral of a controlled path $\left(F,F^{\prime }\right)$ against a rough path S=(S,S). As noted in Friz and Hairer (2020, Remark 4.12), one can actually define a more general integral of a controlled path $\left(F,F^{\prime }\right)$ against another controlled path $\left(G,G^{\prime }\right)$.

Lemma 2.6 Let S=(S,S) be a *p*‐rough path, and let $\left(F,F^{\prime }\right),\left(G,G^{\prime }\right)\in V_{S}^{q}$ be two controlled paths with remainders $R^{F}$ and $R^{G}$, respectively. Then the limit

(5) $$\int_{0}^{T}F_{u}\;dG_{u}:=\lim_{|P|\to 0}\sum_{[s,t]\in P}F_{s}G_{s,t}+F_{s}^{\prime }G_{s}^{\prime }S_{s,t}$$

exists along every sequence of partitions P of the interval [0, *T*] with mesh size |P| tending to zero, and comes with the estimate

(6) $$\begin{array}{c} \begin{array}{cc} & |\int_{s}^{t}F_{u}\;dG_{u}-F_{s}G_{s,t}-F_{s}^{\prime }G_{s}^{\prime }S_{s,t}|\\ & \;\leq C\left(∥F^{\prime }{∥}_{\infty }{\left(∥G^{\prime }{∥}_{q,[s,t]}^{q}+{∥S∥}_{p,[s,t]}^{p}\right)}^{\frac{1}{r}}{∥S∥}_{p,[s,t]}+{∥F∥}_{p,[s,t]}{∥R^{G}∥}_{r,[s,t]}\right.\\ & \;\;\;\;\left.+\;∥R^{F}{∥}_{r,[s,t]}∥G^{\prime }{∥}_{\infty }{∥S∥}_{p,[s,t]}+∥F^{\prime }G^{\prime }{∥}_{q,[s,t]}{∥S∥}_{\frac{p}{2},\left[s,t\right]}\right), \end{array} \end{array}$$

where the constant *C* depends only on p,q, and *r*.

Set $\Xi_{s,t}:=F_{s}G_{s,t}+F_{s}^{\prime }G_{s}^{\prime }S_{s,t}$ and $\delta \Xi_{s,u,t}:=\Xi_{s,t}-\Xi_{s,u}-\Xi_{u,t}$ for 0≤s≤u≤t≤T. Using Chen's relation (2), one can show that

(7) $$\delta \Xi_{s,u,t}=-F_{s}^{\prime }G_{s,u}^{\prime }S_{s,u}S_{u,t}-F_{s,u}R_{u,t}^{G}-R_{s,u}^{F}G_{u}^{\prime }S_{u,t}-{\left(F^{\prime }G^{\prime }\right)}_{s,u}S_{u,t}.$$

Since 1/r=1/p+1/q, Young's inequality gives

$$\begin{array}{cc} |-F_{s}^{\prime }G_{s,u}^{\prime }S_{s,u}S_{u,t}| & \leq ∥F^{\prime }{∥}_{\infty }∥G^{\prime }{∥}_{q,[s,u]}{∥S∥}_{p,[s,u]}{∥S∥}_{p,[u,t]}\\ & ≲∥F^{\prime }{∥}_{\infty }{\left(∥G^{\prime }{∥}_{q,[s,u]}^{q}+{∥S∥}_{p,[s,u]}^{p}\right)}^{\frac{1}{r}}{∥S∥}_{p,[u,t]}=w_{1}{\left(s,u\right)}^{\frac{1}{r}}w_{2}{\left(u,t\right)}^{\frac{1}{p}}, \end{array}$$

where $w_{1}\left(s,u\right):=∥F^{\prime }{∥}_{\infty }^{r}(∥G^{\prime }{∥}_{q,[s,u]}^{q}+{∥S∥}_{p,[s,u]}^{p})$ and $w_{2}\left(u,t\right):={∥S∥}_{p,[u,t]}^{p}$ are control functions. Treating the other three terms on the right‐hand side of Equation (7) similarly, we deduce the hypotheses of the generalized sewing lemma (Friz and Zhang, 2018, Theorem 2.5), from which the result follows.□

Rough integration offers strong pathwise stability estimates, and may be viewed as arguably the most general pathwise integration theory, generalizing classical notions of integration such as those of Riemann–Stieltjes, Young and Föllmer, and allowing one to treat many well‐known stochastic processes as integrators; see, for example, Friz and Hairer (2020). However, from the perspective of mathematical finance, rough integration comes with one apparent flaw: the definition of rough integral (3) is based on so‐called “compensated” Riemann sums, and thus does not (at first glance) come with the natural interpretation as the capital gain process associated to an investment in a financial market. Indeed, let us suppose that *S* represents the asset prices on a financial market and *F* an investment strategy. In this case, neither the associated rough path S=(S,S) nor the controlled path $\left(F,F^{\prime }\right)$, assuming they exist, are uniquely determined by *S* and *F*, but rather the value of the rough integral $\int_{0}^{T}F_{u}\;dS_{u}$ will depend in general on the choices of S and $F^{\prime }$. Moreover, the financial meaning of the term $F_{s}^{\prime }S_{s,t}$ appearing in the compensated Riemann sum in Equation (3) is far from obvious.

As observed in Perkowski and Prömel (2016), the aforementioned drawback of rough integration from a financial perspective can be resolved by introducing the following property of the price path *S*.

(RIE) Let p∈(2,3) and let $P^{n}=\left\{0=t_{0}^{n}<t_{1}^{n}<\cdots <t_{N_{n}}^{n}=T\right\}$, n∈N, be a sequence of partitions of the interval [0, *T*], such that $|P^{n}|\to 0$ as n→∞. For $S\in C(\left[0,T\right];R^{d})$, we define $S^{n}:\left[0,T\right]\to R^{d}$ by

$$S_{t}^{n}:=S_{T}1_{\left\{T\right\}}\left(t\right)+\sum_{k=0}^{N_{n}-1}S_{t_{k}^{n}}1_{\left[t_{k}^{n},t_{k+1}^{n}\right)}\left(t\right),\;t\in \left[0,T\right],$$

for each n∈N. We assume that

- the Riemann sums $\int_{0}^{t}S_{u}^{n}⊗dS_{u}:=\sum_{k=0}^{N_{n}-1}S_{t_{k}^{n}}⊗S_{t_{k}^{n}\wedge t,t_{k+1}^{n}\wedge t}$ converge uniformly as n→∞ to a limit, which we denote by $\int_{0}^{t}S_{u}⊗dS_{u}$, t∈[0,T],
- and that there exists a control function *c* such that^1^
$$\underset{\left(s,t\right)\in \Delta_{\left[0,T\right]}}{\sup}\frac{|S_{s,t}{|}^{p}}{c(s,t)}+\underset{n\in N}{\sup}\;\underset{0\leq k<\ell \leq N_{n}}{\sup}\frac{|\int_{t_{k}^{n}}^{t_{\ell }^{n}}S_{u}^{n}⊗dS_{u}-S_{t_{k}^{n}}⊗S_{t_{k}^{n},t_{\ell }^{n}}{|}^{\frac{p}{2}}}{c(t_{k}^{n},t_{\ell }^{n})}\leq 1.$$

Definition 2.7 A path $S\in C(\left[0,T\right];R^{d})$ is said to satisfy (RIE) with respect to *p* and ${\left(P^{n}\right)}_{n\in N}$, if *p*, ${\left(P^{n}\right)}_{n\in N}$ and *S* together satisfy Property (RIE).

As discussed in detail in Perkowski and Prömel (2016), if a path $S\in C(\left[0,T\right];R^{d})$ satisfies (RIE) with respect to *p* and ${\left(P^{n}\right)}_{n\in N}$, then *S* can be enhanced to a *p*‐rough path S=(S,S) by setting

(8) $$S_{s,t}:=\int_{s}^{t}S_{u}⊗dS_{u}-S_{s}⊗S_{s,t},\;\text{for}\;\left(s,t\right)\in \Delta_{\left[0,T\right]}.$$

In other words, Property (RIE) ensures the existence of a rough path associated to the path *S*. The advantage of the (more restrictive) Property (RIE) is that it guarantees that the corresponding rough integrals can be well approximated by classical left‐point Riemann sums, as we will see in Section 2.4, thus allowing us to restore the financial interpretation of such integrals as capital processes.

Remark 2.8 The assumption that the underlying price paths satisfy Property (RIE) appears to be rather natural in the context of portfolio theory. Indeed, in stochastic portfolio theory, the price processes are commonly modeled as semimartingales fulfilling the condition of “no unbounded profit with bounded risk” (NUPBR); see, for example, Fernholz (2002). The condition (NUPBR) is also essentially the minimal condition required to ensure that expected utility maximization problems are well‐posed; see Karatzas and Kardaras (2007); Imkeller and Perkowski (2015). As established in Perkowski and Prömel (2016, Proposition 2.7 and Remark 4.16), the sample paths of semimartingales fulfilling (NUPBR) almost surely satisfy Property (RIE) with respect to every p∈(2,3) and a suitably chosen sequence of partitions.

### 2.3 The bracket process and a rough Itô formula

A vital tool in many applications of stochastic calculus is Itô's formula, and it will also be an important ingredient in our contribution to portfolio theory. Usually, (pathwise) Itô formulae are based on the notion of quadratic variation. In rough path theory, a similar role as that of the quadratic variation is played by the so‐called bracket of a rough path, compare Friz and Hairer (2020, Definition 5.5).

Definition 2.9 Let S=(S,S) be a *p*‐rough path and let Sym(S) denote the symmetric part of S. The *bracket* of S is defined as the path $\left[S\right]:\left[0,T\right]\to R^{d\times d}$ given by

$${\left[S\right]}_{t}:=S_{0,t}⊗S_{0,t}-2\text{Sym}\left(S_{0,t}\right),\;t\in \left[0,T\right].$$

The bracket of a rough path allows one to derive Itô formulae for rough paths. For this purpose, note that [S] is a continuous path of finite p/2‐variation, which can be seen from the observation that

$${\left[S\right]}_{s,t}={\left[S\right]}_{t}-{\left[S\right]}_{s}=S_{s,t}⊗S_{s,t}-2\text{Sym}\left(S_{s,t}\right),\;\text{for all}\;\left(s,t\right)\in \Delta_{\left[0,T\right]}.$$

The following Itô formula for rough paths can be proven almost exactly as the one in Friz and Hairer (2020, Theorem 7.7), so we will omit its proof here; see also Friz and Zhang (2018, Theorem 2.12).

Proposition 2.10 Let S=(S,S) be a *p*‐rough path and let $\Gamma \in C^{\frac{p}{2}\text{-var}}\left(\left[0,T\right];R^{d}\right)$. Suppose that $F,F^{\prime }$ and $F^{\prime \prime }$ are such that $\left(F,F^{\prime }\right),\left(F^{\prime },F^{\prime \prime }\right)\in V_{S}^{q}$, and $F=\int_{0}^{\cdot }F_{u}^{\prime }\;dS_{u}+\Gamma$. If $g\in C^{p+\varepsilon }$ for some ε>0, then, for every t∈[0,T], we have

$$g\left(F_{t}\right)=g\left(F_{0}\right)+\int_{0}^{t}Dg\left(F_{u}\right)F_{u}^{\prime }\;dS_{u}+\int_{0}^{t}Dg\left(F_{u}\right)\;d\Gamma_{u}+\frac{1}{2}\int_{0}^{t}D^{2}g\left(F_{u}\right)\left(F_{u}^{\prime }⊗F_{u}^{\prime }\right)\;d{\left[S\right]}_{u}.$$

Assuming Property (RIE), it turns out that the bracket [S] of a rough path S=(S,S) does coincide precisely with the quadratic variation of the path *S* in the sense of Föllmer (1981).

Lemma 2.11 Suppose that $S\in C(\left[0,T\right];R^{d})$ satisfies (RIE) with respect to *p* and ${\left(P^{n}\right)}_{n\in N}$. Let S=(S,S) be the associated rough path as defined in Equation (8). Then, the bracket [S] has finite total variation, and is given by

$${\left[S\right]}_{t}=\lim_{n\to \infty }\sum_{k=0}^{N_{n}-1}S_{t_{k}^{n}\wedge t,t_{k+1}^{n}\wedge t}⊗S_{t_{k}^{n}\wedge t,t_{k+1}^{n}\wedge t},$$

where the convergence is uniform in t∈[0,T].

The (i,j)‐component of ${\left[S\right]}_{t}$ is given by

$${\left[S\right]}_{t}^{ij}=S_{0,t}^{i}S_{0,t}^{j}-S_{0,t}^{ij}-S_{0,t}^{ji}=S_{t}^{i}S_{t}^{j}-S_{0}^{i}S_{0}^{j}-\int_{0}^{t}S_{u}^{i}\;dS_{u}^{j}-\int_{0}^{t}S_{u}^{j}\;dS_{u}^{i}.$$

The result then follows from Lemmas 4.17 and 4.22 in Perkowski and Prömel (2016).□

In view of Lemma 2.11, when assuming Property (RIE), we also refer to the bracket [S] as the *quadratic variation* of *S*.

### 2.4 Rough integrals as limits of Riemann sums

As previously mentioned, the main motivation to introduce Property (RIE) is to obtain the rough integral as a limit of left‐point Riemann sums, in order to restore the interpretation of the rough integral as the capital process associated with a financial investment. Indeed, we present the following extension of Perkowski and Prömel (2016, Theorem 4.19), which will be another central tool in our pathwise portfolio theory. The proof of Theorem 2.12 is postponed to Appendix B.

Theorem 2.12 Suppose that $S\in C(\left[0,T\right];R^{d})$ satisfies (RIE) with respect to *p* and ${\left(P^{n}\right)}_{n\in N}$. Let q≥p such that 2/p+1/q>1. Let $f\in C^{p+\varepsilon }$ for some ε>0, so that in particular $\left(f\left(S\right),Df\left(S\right)\right)\in V_{S}^{q}$. Then, for any $\left(Y,Y^{\prime }\right)\in V_{S}^{q}$, the integral of $\left(Y,Y^{\prime }\right)$ against (f(S),Df(S)), as defined in Lemma 2.6, is given by

(9) $$\int_{0}^{t}Y_{u}\;df{\left(S\right)}_{u}=\lim_{n\to \infty }\sum_{k=0}^{N_{n}-1}Y_{t_{k}^{n}}f{\left(S\right)}_{t_{k}^{n}\wedge t,t_{k+1}^{n}\wedge t},$$

where the convergence is uniform in t∈[0,T].

As an immediate consequence of Theorem 2.12, assuming Property (RIE), we note that, for $\left(Y,Y^{\prime }\right)\in V_{S}^{q}$, the rough integral

(10) $$\int_{0}^{t}Y_{u}\;dS_{u}=\lim_{n\to \infty }\sum_{k=0}^{N_{n}-1}Y_{t_{k}^{n}}S_{t_{k}^{n}\wedge t,t_{k+1}^{n}\wedge t},$$

and indeed the more general rough integral in Equation (9), is independent of the Gubinelli derivative $Y^{\prime }$. However, in the spirit of Föllmer's pathwise quadratic variation and integration, the right‐hand sides of Equations (9) and (10) do in general depend on the sequence of partitions ${\left(P^{n}\right)}_{n\in N}$.

## 3 PATHWISE (RELATIVE) PORTFOLIO WEALTH PROCESSES AND MASTER FORMULA

In this section, we consider pathwise portfolio theory on the rough path foundation presented in Section 2. In particular, we study the growth of wealth processes relative to the market portfolio, and provide an associated pathwise master formula analogous to that of classical SPT, compare Fernholz (1999); Strong (2014); Schied et al. (2018). We start by introducing the basic assumptions on the underlying financial market.

### 3.1 The financial market

Since we want to investigate the long‐run behavior of wealth processes, we consider the price trajectories of *d* assets on the time interval [0, ∞). As is common in SPT, we do not include default risk—that is, all prices are assumed to be strictly positive—and we do not distinguish between risk‐free and risky assets.

A partition P of the interval [0, ∞) is a strictly increasing sequence of points ${\left(t_{i}\right)}_{i\geq 0}\subset \left[0,\infty \right)$, with $t_{0}=0$ and such that $t_{i}\to \infty$ as i→∞. Given any T>0, we denote by P([0,T]) the restriction of the partition P∪{T} to the interval [0, *T*], that is, P([0,T]):=(P∪{T})∩[0,T]. For a path $S:\left[0,\infty \right)\to R^{d}$, we write ${S|}_{\left[0,T\right]}$ for the restriction of *S* to [0, *T*], and we set $R_{+}:=\left(0,\infty \right)$.

Definition 3.1 For a fixed p∈(2,3), we say that a path $S\in C(\left[0,\infty \right);R_{+}^{d})$ is a *price path*, if there exists a sequence of partitions ${\left(P_{S}^{n}\right)}_{n\in N}$ of the interval [0, ∞), with vanishing mesh size on compacts, such that, for all T>0, the restriction ${S|}_{\left[0,T\right]}$ satisfies (RIE) with respect to *p* and ${\left(P_{S}^{n}\left(\left[0,T\right]\right)\right)}_{n\in N}$.

We denote the family of all such price paths by $\Omega_{p}$.

It seems to be natural to allow the partitions ${\left(P_{S}^{n}\right)}_{n\in N}$ to depend on the price path *S*, since partitions are typically given via stopping times in stochastic frameworks.

Throughout the remainder of the paper, we adopt the following assumption on the regularity parameters.

Assumption 3.2 Let p∈(2,3), q≥p, and r>1 be given such that

$$\frac{2}{p}+\frac{1}{q}>1\;\text{and}\;\frac{1}{r}=\frac{1}{p}+\frac{1}{q}.$$

In particular, we note that 1<p/2≤r<p≤q<∞.

By Property (RIE), we can (and do) associate to every price path $S\in \Omega_{p}$ the *p*‐rough path S=(S,S), as defined in Equation (8). We can then define the *market covariance* as the matrix $a={\left[a^{ij}\right]}_{1\leq i,j\leq d}$, with (i,j)‐component given by the measure

(11) $$a^{ij}\left(ds\right):=\frac{1}{S_{s}^{i}S_{s}^{j}}\;d{\left[S\right]}_{s}^{ij}.$$

Although we do not work in a probabilistic setting and thus should not, strictly speaking, talk about covariance in the probabilistic sense, the relation (11) is consistent with classical SPT (with the bracket process replaced by the quadratic variation), and it turns out to still be a useful quantity in pathwise frameworks, compare Schied and Voloshchenko (2016); Schied et al. (2018).

### 3.2 Pathwise portfolio wealth processes

We now introduce admissible portfolios and the corresponding wealth processes on the market defined above. To this end, we first fix the notation:

$$\begin{array}{c} \Delta^{d}:=\{x=\left(x^{1},\text{…},x^{d}\right)\in R^{d}\;:\;\sum_{i=1}^{d}x^{i}=1\}, \end{array}$$

$\Delta_{+}^{d}:=\left\{x\in \Delta^{d}:x^{i}>0\;\;\forall i=1,\text{…},d\right\}$ and ${\bar{\Delta }}_{+}^{d}:=\left\{x\in \Delta^{d}:x^{i}\geq 0\;\;\forall i=1,\text{…},d\right\}$.

Definition 3.3 We say that a path $F:\left[0,\infty \right)\to R^{d}$ is an *admissible strategy* if, for every T>0, there exists a path $F^{\prime }:\left[0,T\right]\to L\left(R^{d};R^{d}\right)$ such that $\left(F{|}_{\left[0,T\right]},F^{\prime }\right)\in V_{S}^{q}$ is a controlled path with respect to *S* (in the sense of Definition 2.3). We say that an admissible strategy π is a *portfolio* for *S* if additionally $\pi_{t}\in \Delta^{d}$ for all t∈[0,∞).

Remark 3.4 As explained in Friz and Hairer (2020, Remark 4.7), if *S* is sufficiently regular then, given an admissible strategy *F*, there could exist multiple different Gubinelli derivatives $F^{\prime }$ such that the pair $\left(F,F^{\prime }\right)$ defines a valid controlled path with respect to *S*. However, thanks to Property (RIE), Theorem 2.12 shows that the rough integral ∫FdS can be expressed as a limit of Riemann sums, which only involve *F* and *S*, and, therefore, is independent of the choice of $F^{\prime }$. Thus, the choice of the Gubinelli derivative $F^{\prime }$ is unimportant, provided that at least one exists. Indeed, one could define an equivalence relation ∼ on $V_{S}^{q}$ such that $\left(F,F^{\prime }\right)∼\left(G,G^{\prime }\right)$ if F=G, and define the family of admissible strategies as elements of the quotient space $V_{S}^{q}/∼$. By a slight abuse of notation, we shall, therefore, sometimes write simply $F\in V_{S}^{q}$ instead of $\left(F,F^{\prime }\right)\in V_{S}^{q}$.

Remark 3.5 While the admissible class of portfolios introduced in Definition 3.3 allows for a pathwise (model‐free) analysis (without notions like filtration or predictability), it also covers the most frequently applied classes of functionally generated portfolios—see Fernholz (1999)—and their generalizations as considered in, for example, Strong (2014) and Schied et al. (2018). Indeed, every path‐dependent functionally generated portfolio, which is sufficiently smooth in the sense of Dupire (2019) (see also Cont and Fournié (2010)), is a controlled path and thus an admissible strategy, as shown in Ananova (2020).

In the present work, we will principally focus on “adapted” strategies *F*, in the sense that *F* is a controlled path, as in Definition 3.3, with $F_{t}$ being a measurable function of ${S|}_{\left[0,t\right]}$ for each t∈[0,∞). In other words, if *S* is modeled by a stochastic process, then we require *F* to be adapted to the natural filtration generated by *S*. Clearly, such adapted admissible strategies are reasonable choices in the context of mathematical finance.

A portfolio $\pi =(\pi^{1},\cdots ,\pi^{d})$ represents the ratio of the investor's wealth invested into each of the *d* assets. As is usual, we normalize the initial wealth to be 1, since in the following, we will only be concerned with the long‐run growth. Suppose $S\in \Omega_{p}$ with corresponding sequence ${\left(P_{S}^{n}\right)}_{n\in N}$ of partitions. If we restrict the rebalancing according to the portfolio π to the discrete times given by $P_{S}^{n}={\left(t_{j}^{n}\right)}_{j\in N}$, then the corresponding wealth process $W^{n}$ satisfies

$$W_{t}^{n}=1+\sum_{j=1}^{\infty }\frac{\pi_{t_{j}}W_{t_{j}}^{n}}{S_{t_{j}}}S_{t_{j}\wedge t,t_{j+1}\wedge t}=1+\sum_{j=1}^{\infty }\sum_{i=1}^{d}\frac{\pi_{t_{j}}^{i}W_{t_{j}}^{n}}{S_{t_{j}}^{i}}S_{t_{j}\wedge t,t_{j+1}\wedge t}^{i}$$

with $t_{j}\wedge t:=\min\left\{t_{j},t\right\}$. Taking the limit to continuous‐time (i.e., n→∞) and keeping Property (RIE) in mind, we observe that the wealth process $W^{\pi }$ associated to the portfolio π should satisfy

(12) $$W_{t}^{\pi }=1+\int_{0}^{t}\frac{\pi_{s}W_{s}^{\pi }}{S_{s}}\;dS_{s},\;t\in \left[0,\infty \right).$$

Analogously to (classical) SPT (e.g., Karatzas and Kardaras (2007) or Schied et al. (2018)), the wealth process associated to a portfolio may be expressed as a (rough) exponential.

Lemma 3.6 Let π be a portfolio for $S\in \Omega_{p}$. Then the wealth process $W^{\pi }$ (with unit initial wealth), given by

$$W_{t}^{\pi }:=\exp\left(\int_{0}^{t}\frac{\pi_{s}}{S_{s}}\;dS_{s}-\frac{1}{2}\sum_{i,j=1}^{d}\int_{0}^{t}\frac{\pi_{s}^{i}\pi_{s}^{j}}{S_{s}^{i}S_{s}^{j}}\;d{\left[S\right]}_{s}^{ij}\right),\;t\in \left[0,\infty \right),$$

satisfies Equation (12), where $\int_{0}^{t}\frac{\pi_{s}}{S_{s}}\;dS_{s}$ is the rough integral of the controlled path π/S with respect to rough path S, and $\int_{0}^{t}\frac{\pi_{s}^{i}\pi_{s}^{j}}{S_{s}^{i}S_{s}^{j}}\;d{\left[S\right]}_{s}^{ij}$ is the usual Riemann–Stieltjes integral with respect to the (i,j)‐component of the (finite variation) bracket [S].

Note that, since 1/S=f(S) with the smooth function $f\left(x\right)=\left(1/x^{1},\text{…},1/x^{d}\right)$ on $R_{+}^{d}$, the pair $\left(1/S,Df\left(S\right)\right)\in V_{S}^{p}\subset V_{S}^{q}$ is a controlled path. Therefore, for each portfolio $\pi \in V_{S}^{q}$, we can define the quotient $\pi /S=(\pi^{1}/S^{1},\text{…},\pi^{d}/S^{d})$, which gives an element $\left(\pi /S,{\left(\pi /S\right)}^{\prime }\right)$ in $V_{S}^{q}$; see Lemma A.1.

Setting $Z:=\int_{0}^{\cdot }\frac{\pi_{s}}{S_{s}}\;dS_{s}$, by Lemma B.1, we have that

$$\left[Z\right]=\int_{0}^{\cdot }\left(\frac{\pi_{s}}{S_{s}}⊗\frac{\pi_{s}}{S_{s}}\right)\;d{\left[S\right]}_{s}=\sum_{i,j=1}^{d}\int_{0}^{\cdot }\frac{\pi_{s}^{i}\pi_{s}^{j}}{S_{s}^{i}S_{s}^{j}}\;d{\left[S\right]}_{s}^{ij},$$

where Z is the canonical rough path lift of *Z* (see Section A.3). We then have that $W_{t}^{\pi }=\exp\left(Z_{t}-\frac{1}{2}{\left[Z\right]}_{t}\right)$, so that, by Lemma A.5, $W^{\pi }$ satisfies

$$W_{t}^{\pi }=1+\int_{0}^{t}W_{s}^{\pi }\;dZ_{s},\;t\in \left[0,\infty \right).$$

By Lemma A.4 and Proposition A.2, it then follows that $W^{\pi }$ satisfies Equation (12).□

Remark 3.7 Every portfolio π can be associated to a self‐financing admissible strategy ξ by setting $\xi_{t}^{i}:=\pi_{t}^{i}W_{t}^{\pi }/S_{t}^{i}$ for i=1,…,d. Indeed, we have that $W_{t}^{\pi }=\sum_{i=1}^{d}\xi_{t}^{i}S_{t}^{i}$, and that

$$W_{t}^{\pi }=1+\int_{0}^{t}\frac{\pi_{s}W_{s}^{\pi }}{S_{s}}\;dS_{s}=1+\int_{0}^{t}\xi_{s}\;dS_{s},\;t\in \left[0,\infty \right),$$

so that ξ is self‐financing.

As in the classical setup of SPT (e.g., Fernholz (2002)) we introduce the market portfolio as a reference portfolio.

Lemma 3.8 The path $\mu :\left[0,\infty \right)\to \Delta_{+}^{d}$, defined by $\mu_{t}^{i}:=\frac{S_{t}^{i}}{S_{t}^{1}+\cdots +S_{t}^{d}}$ for i=1,…,d, is a portfolio for $S\in \Omega_{p}$, called the *market portfolio* (or *market weights process*). The corresponding wealth process (with initial wealth 1) is given by

$$W_{t}^{\mu }=\frac{S_{t}^{1}+\cdots +S_{t}^{d}}{S_{0}^{1}+\cdots +S_{0}^{d}}.$$

Since μ is a smooth function of *S*, it is a controlled path with respect to *S*, and is, therefore, an admissible strategy. Since $\mu_{t}^{1}+\cdots +\mu_{t}^{d}=1$, we see that μ is indeed a portfolio.

Let $f\left(x\right):=\log\left(x^{1}+\cdots +x^{d}\right)$ for $x\in R_{+}^{d}$. By the Itô formula for rough paths (Proposition 2.10), it follows that

$$\begin{array}{cc} f\left(S_{t}\right)-f\left(S_{0}\right) & =\int_{0}^{t}\left(\frac{1}{S_{s}^{1}+\cdots +S_{s}^{d}},\;\text{…},\;\frac{1}{S_{s}^{1}+\cdots +S_{s}^{d}}\right)dS_{s}-\frac{1}{2}\int_{0}^{t}\left(\frac{\mu_{s}}{S_{s}}⊗\frac{\mu_{s}}{S_{s}}\right)\;d{\left[S\right]}_{s}\\ & =\int_{0}^{t}\frac{\mu_{s}}{S_{s}}\;dS_{s}-\frac{1}{2}\sum_{i,j=1}^{d}\int_{0}^{t}\frac{\mu_{s}^{i}\mu_{s}^{j}}{S_{s}^{i}S_{s}^{j}}\;d{\left[S\right]}_{s}^{ij}, \end{array}$$

where we used the fact that $\frac{\mu_{s}^{i}}{S_{s}^{i}}=\frac{1}{S_{s}^{1}+\cdots +S_{s}^{d}}$. By Lemma 3.6, the right‐hand side is equal to $\log W_{t}^{\mu }$, so that

$$W_{t}^{\mu }=\exp\left(f\left(S_{t}\right)-f\left(S_{0}\right)\right)=\frac{S_{t}^{1}+\cdots +S_{t}^{d}}{S_{0}^{1}+\cdots +S_{0}^{d}}.$$

□

### 3.3 Formulae for the growth of wealth processes

In this subsection, we derive pathwise versions of classical formulae of SPT—see Fernholz (1999)—which describe the dynamics of the relative wealth of a portfolio with respect to the market portfolio; compare Schied et al. (2018) for analogous results relying on Föllmer's pathwise integration.

Given a portfolio π, we define the relative covariance of π by $\tau^{\pi }={\left[\tau_{ij}^{\pi }\right]}_{1\leq i,j\leq d}$, where

(13) $$\tau_{ij}^{\pi }\left(ds\right):={\left(\pi_{s}-e_{i}\right)}^{⊤}a\left(ds\right)\left(\pi_{s}-e_{j}\right),$$

where ${\left(e_{i}\right)}_{1\leq i\leq d}$ denotes the canonical basis of $R^{d}$, and we recall a(ds) as defined in Equation (11).

Henceforth, we will write

(14) $$V^{\pi }:=\frac{W^{\pi }}{W^{\mu }}$$

for the relative wealth of a portfolio π with respect to the market portfolio μ.

Proposition 3.9 Let π be a portfolio for $S\in \Omega_{p}$, and let μ be the market portfolio as above. We then have that

(15) $$\log V_{t}^{\pi }=\int_{0}^{t}\frac{\pi_{s}}{\mu_{s}}\;d\mu_{s}-\frac{1}{2}\sum_{i,j=1}^{d}\int_{0}^{t}\pi_{s}^{i}\pi_{s}^{j}\tau_{ij}^{\mu }\left(ds\right),\;t\in \left[0,\infty \right).$$

Remark 3.10 The integral $\int_{0}^{t}\frac{\pi_{s}}{\mu_{s}}\;d\mu_{s}$ appearing in Equation (15) is interpreted as the rough integral of the *S*‐controlled path π/μ against the *S*‐controlled path μ in the sense of Lemma 2.6. By Theorem 2.12, the integral $\int_{0}^{t}\frac{\pi_{s}}{\mu_{s}}\;d\mu_{s}$ can also be expressed as a limit of left‐point Riemann sums, which justifies the financial meaning of Equation (15).

Proof of Proposition 3.9 *Step 1*. By the Itô formula for rough paths (Proposition 2.10), with the usual notational convention $\log x=\sum_{i=1}^{d}\log x^{i}$, we have

$$\log S_{t}=\log S_{0}+\int_{0}^{t}\frac{1}{S_{s}}\;dS_{s}-\frac{1}{2}\sum_{i=1}^{d}\int_{0}^{t}\frac{1}{{\left(S_{s}^{i}\right)}^{2}}\;d{\left[S\right]}_{s}^{ii},\;t\in \left[0,\infty \right).$$

Since π and logS are *S*‐controlled paths, we can define the integral of π against logS in the sense of Lemma 2.6. By the associativity of rough integration (Proposition A.2), we have

$$\int_{0}^{t}\pi_{s}\;d\log S_{s}=\int_{0}^{t}\frac{\pi_{s}}{S_{s}}\;dS_{s}-\frac{1}{2}\sum_{i=1}^{d}\int_{0}^{t}\frac{\pi_{s}^{i}}{{\left(S_{s}^{i}\right)}^{2}}\;d{\left[S\right]}_{s}^{ii}.$$

It is convenient to introduce the excess growth rate of the portfolio π, given by

$$\gamma_{\pi }^{*}\left(ds\right):=\frac{1}{2}\left(\sum_{i=1}^{d}\pi_{s}^{i}a^{ii}\left(ds\right)-\sum_{i,j=1}^{d}\pi_{s}^{i}\pi_{s}^{j}a^{ij}\left(ds\right)\right).$$

By Lemma 3.6, we have that

(16) $$\log W_{t}^{\pi }=\int_{0}^{t}\frac{\pi_{s}}{S_{s}}\;dS_{s}-\frac{1}{2}\sum_{i,j=1}^{d}\int_{0}^{t}\pi_{s}^{i}\pi_{s}^{j}a^{ij}\left(ds\right)=\int_{0}^{t}\pi_{s}\;d\log S_{s}+\gamma_{\pi }^{*}\left(\left[0,t\right]\right).$$

In particular, this implies that

(17) $$\log V_{t}^{\pi }=\int_{0}^{t}\left(\pi_{s}-\mu_{s}\right)\;d\log S_{s}+\gamma_{\pi }^{*}\left(\left[0,t\right]\right)-\gamma_{\mu }^{*}\left(\left[0,t\right]\right).$$

*Step 2*. By Lemma 3.8 and Equation (16), we have

(18) $$\begin{array}{cc} \log\mu_{t}^{i} & =\log\mu_{0}^{i}+\log S_{t}^{i}-\log S_{0}^{i}-\log W_{t}^{\mu }\\ & =\log\mu_{0}^{i}+\log S_{t}^{i}-\log S_{0}^{i}-\int_{0}^{t}\mu_{s}\;d\log S_{s}-\gamma_{\mu }^{*}\left(\left[0,t\right]\right)\\ & =\log\mu_{0}^{i}+\int_{0}^{t}\left(e_{i}-\mu_{s}\right)\;d\log S_{s}-\gamma_{\mu }^{*}\left(\left[0,t\right]\right). \end{array}$$

By part (ii) of Proposition B.2 and Lemma B.1, we deduce that

(19) $${\left[\log S\right]}_{t}=a\left(\left[0,t\right]\right),\;\text{and}\;{\left[\log\mu \right]}_{t}=\tau^{\mu }\left(\left[0,t\right]\right).$$

Applying the Itô formula for rough paths (Proposition 2.10) to $\exp(\log\mu^{i})$, using the associativity of rough integration (Proposition A.2), and recalling Equation (18), we have

$$\int_{0}^{t}\frac{\pi_{s}^{i}}{\mu_{s}^{i}}\;d\mu_{s}^{i}=\int_{0}^{t}\pi_{s}^{i}\left(e_{i}-\mu_{s}\right)\;d\log S_{s}-\int_{0}^{t}\pi_{s}^{i}\;d\gamma_{\mu }^{*}\left(ds\right)+\frac{1}{2}\int_{0}^{t}\pi_{s}^{i}\;d{\left[\log\mu \right]}_{s}^{ii}.$$

Using Equation (19) and summing over i=1,…,d, we obtain

(20) $$\int_{0}^{t}\frac{\pi_{s}}{\mu_{s}}\;d\mu_{s}=\int_{0}^{t}\left(\pi_{s}-\mu_{s}\right)\;d\log S_{s}-\gamma_{\mu }^{*}\left(\left[0,t\right]\right)+\frac{1}{2}\sum_{i=1}^{d}\int_{0}^{t}\pi_{s}^{i}\tau_{ii}^{\mu }\left(ds\right).$$

*Step 3*. Taking the difference of Equations (17) and (20), we have

$$\log V_{t}^{\pi }=\int_{0}^{t}\frac{\pi_{s}}{\mu_{s}}\;d\mu_{s}+\gamma_{\pi }^{*}\left(\left[0,t\right]\right)-\frac{1}{2}\sum_{i=1}^{d}\int_{0}^{t}\pi_{s}^{i}\tau_{ii}^{\mu }\left(ds\right).$$

It remains to note that

$$\gamma_{\pi }^{*}\left(\left[0,t\right]\right)=\frac{1}{2}\left(\sum_{i=1}^{d}\int_{0}^{t}\pi_{s}^{i}\tau_{ii}^{\mu }\left(ds\right)-\sum_{i,j=1}^{d}\int_{0}^{t}\pi_{s}^{i}\pi_{s}^{j}\tau_{ij}^{\mu }\left(ds\right)\right),$$

which follows from a straightforward calculation; see, for example, Fernholz (2002, Lemma 1.3.4).□

While Definition 3.3 allows for rather general portfolios, so‐called functionally generated portfolios are the most frequently considered ones in SPT. In a pathwise setting, such portfolios and the corresponding master formula were studied previously in Schied et al. (2018) and Cuchiero et al. (2019). We conclude this section by deriving such a master formula for functionally generated portfolios in the present (rough) pathwise setting.

Let *G* be a strictly positive function in $C^{p+\varepsilon }\left(\Delta_{+}^{d};R_{+}\right)$ for some ε>0. One can verify that $\nabla \log G\left(\mu \right)\in V_{\mu }^{q}$ is a μ‐controlled path for a suitable choice of *q* (see Example 2.4), and is, therefore, also an *S*‐controlled path by Lemma A.4. Since the product of controlled paths is itself a controlled path (by Lemma A.1), we see that the path π defined by

(21) $$\pi_{t}^{i}:=\mu_{t}^{i}\left(\frac{\partial }{\partial x_{i}}\log G\left(\mu_{t}\right)+1-\sum_{k=1}^{d}\mu_{t}^{k}\frac{\partial }{\partial x_{k}}\log G\left(\mu_{t}\right)\right),\;t\in \left[0,\infty \right),\;\;i=1,\text{…},d,$$

is a μ‐controlled (and hence also an *S*‐controlled) path, and is indeed a portfolio for $S\in \Omega_{p}$. The function *G* is called a *portfolio generating function*, and we say that *G* *generates* π.

Theorem 3.11 (The master formula) Let $G\in C^{p+\varepsilon }\left(\Delta_{+}^{d};R_{+}\right)$ for some ε>0 be a portfolio generating function, and let π be the portfolio generated by *G*. The wealth of π relative to the market portfolio is given by

$$\log V_{t}^{\pi }=\log\left(\frac{G(\mu_{t})}{G(\mu_{0})}\right)-\frac{1}{2}\sum_{i,j=1}^{d}\int_{0}^{t}\frac{1}{G(\mu_{s})}\frac{\partial^{2}G\left(\mu_{s}\right)}{\partial x_{i}\partial x_{j}}\mu_{s}^{i}\mu_{s}^{j}\tau_{ij}^{\mu }\left(ds\right),\;t\in \left[0,\infty \right).$$

Let g=∇logG(μ), so that $g^{i}=\frac{\partial }{\partial x_{i}}\log G\left(\mu \right)=\frac{1}{G(\mu )}\frac{\partial G}{\partial x_{i}}\left(\mu \right)$ for each i=1,…,d. We can then rewrite Equation (21) as

(22) $$\pi^{i}=\mu^{i}\left(g^{i}+1-\sum_{k=1}^{d}\mu^{k}g^{k}\right),$$

so that $\pi^{i}/\mu^{i}=g^{i}+1-\sum_{k=1}^{d}\mu^{k}g^{k}$. Since $\sum_{i=1}^{d}\mu_{s}^{i}=1$ for all s≥0, we must have that $\sum_{i=1}^{d}\mu_{s,t}^{i}=0$ for all s<t. Thus

$$\int_{0}^{t}\frac{\pi_{s}}{\mu_{s}}\;d\mu_{s}=\lim_{n\to \infty }\sum_{k=0}^{N_{n}-1}\sum_{i=1}^{d}\frac{\pi_{t_{k}^{n}}^{i}}{\mu_{t_{k}^{n}}^{i}}\mu_{t_{k}^{n}\wedge t,t_{k+1}^{n}\wedge t}^{i}=\lim_{n\to \infty }\sum_{k=0}^{N_{n}-1}\sum_{i=1}^{d}g_{t_{k}^{n}}^{i}\mu_{t_{k}^{n}\wedge t,t_{k+1}^{n}\wedge t}^{i}=\int_{0}^{t}g_{s}\;d\mu_{s}.$$

We have from Equation (13) that $\sum_{j=1}^{d}\mu_{s}^{j}\tau_{ij}^{\mu }\left(ds\right)={\left(\mu_{s}-e_{i}\right)}^{⊤}a\left(ds\right)\left(\mu_{s}-\mu_{s}\right)=0$. It follows from this and Equation (22) that

(23) $$\sum_{i,j=1}^{d}\pi_{s}^{i}\pi_{s}^{j}\tau_{ij}^{\mu }\left(ds\right)=\sum_{i,j=1}^{d}g_{s}^{i}g_{s}^{j}\mu_{s}^{i}\mu_{s}^{j}\tau_{ij}^{\mu }\left(ds\right).$$

Recall from Equation (19) that ${\left[\log\mu \right]}_{t}=\tau^{\mu }\left(\left[0,t\right]\right)$. By applying the Itô formula for rough paths (Proposition 2.10) to $\mu^{i}=\exp\left(\log\mu^{i}\right)$, we see that the path $t\mapsto \mu_{t}^{i}-\int_{0}^{t}\mu_{s}^{i}\;d\log\mu_{s}^{i}$ is of finite variation. By part (ii) of Proposition B.2 and Lemma B.1, we, therefore, have that

(24) $${\left[\mu \right]}_{t}^{ij}=\int_{0}^{t}\mu_{s}^{i}\mu_{s}^{j}\;d{\left[\log\mu \right]}_{s}^{ij}=\int_{0}^{t}\mu_{s}^{i}\mu_{s}^{j}\tau_{ij}^{\mu }\left(ds\right).$$

By the Itô formula for rough paths (Proposition 2.10), we then have

$$\begin{array}{cc} \log\left(\frac{G(\mu_{t})}{G(\mu_{0})}\right) & =\int_{0}^{t}g_{s}\;d\mu_{s}+\frac{1}{2}\sum_{i,j=1}^{d}\int_{0}^{t}\left(\frac{1}{G(\mu_{s})}\frac{\partial^{2}G\left(\mu_{s}\right)}{\partial x_{i}\partial x_{j}}-g_{s}^{i}g_{s}^{j}\right)\;d{\left[\mu \right]}_{s}^{ij}\\ & =\int_{0}^{t}\frac{\pi_{s}}{\mu_{s}}\;d\mu_{s}+\frac{1}{2}\sum_{i,j=1}^{d}\int_{0}^{t}\left(\frac{1}{G(\mu_{s})}\frac{\partial^{2}G\left(\mu_{s}\right)}{\partial x_{i}\partial x_{j}}-g_{s}^{i}g_{s}^{j}\right)\mu_{s}^{i}\mu_{s}^{j}\tau_{ij}^{\mu }\left(ds\right). \end{array}$$

Combining this with Equations (15) and (23), we deduce the result.□

## 4 COVER'S UNIVERSAL PORTFOLIOS AND THEIR OPTIMALITY

Like SPT, Cover's universal portfolios (Cover, 1991) aim to give general recipes to construct preference‐free asymptotically “optimal” portfolios; see also Jamshidian (1992) and Cover and Ordentlich (1996). A first link between SPT and these universal portfolios was established in a pathwise framework based on Föllmer integration in Cuchiero et al. (2019) (see also Wong (2015)). In this section, we shall generalize the pathwise theory regarding Cover's universal portfolios developed in Cuchiero et al. (2019) to the present rough path setting.

Cover's universal portfolio is based on the idea of trading according to a portfolio, which is defined as the average over a family A of admissible portfolios. In the spirit of Cuchiero et al. (2019), we introduce pathwise versions of Cover's universal portfolios—that is, portfolios of the form

$$\pi_{t}^{\nu }:=\frac{\int_{A}\pi_{t}V_{t}^{\pi }\;d\nu \left(\pi \right)}{\int_{A}V_{t}^{\pi }\;d\nu \left(\pi \right)},\;t\in \left[0,\infty \right),$$

where ν is a given probability measure on A. In order to find suitable classes A of admissible portfolios, we recall Assumption 3.2 and make the following standing assumption throughout the entire section.

Assumption 4.1 We fix $q^{\prime }>q$ and $r^{\prime }>r$ such that $\frac{2}{p}+\frac{1}{q^{\prime }}>1$ and $\frac{1}{r^{\prime }}=\frac{1}{p}+\frac{1}{q^{\prime }}$.

### 4.1 Admissible portfolios

As a first step to construct Cover's universal portfolios in our rough path setting, we need to find a suitable set of admissible portfolios. To this end, we set

$$V_{\mu }^{q}\left(\left[0,\infty \right);\Delta^{d}\right):=\{\left(\pi ,\pi^{\prime }\right):\forall \;T>0,\;\left(\pi ,\pi^{\prime }\right){|}_{\left[0,T\right]}\in V_{\mu }^{q}\left(\left[0,T\right];\Delta^{d}\right)\}.$$

Then, for some fixed control function $c_{\mu }$, which controls the *p*‐variation norm of the market portfolio μ, and for some M>0, we introduce a class of *admissible portfolios* as the set

(25) $$A^{M,q}\left(c_{\mu }\right):=\left\{\left(\pi ,\pi^{\prime }\right)\in V_{\mu }^{q}\left(\left[0,\infty \right);\Delta^{d}\right)\;:\;\begin{array}{c} |\frac{\pi_{0}}{\mu_{0}}\left|+\right|{\left(\frac{\pi }{\mu }\right)}_{0}^{\prime }|\leq M,\\ \underset{s\leq t}{\sup}\frac{{\left|{\left(\frac{\pi }{\mu }\right)}_{s,t}^{\prime }\right|}^{q}}{c_{\mu }\left(s,t\right)}+\underset{s\leq t}{\sup}\frac{{\left|R_{s,t}^{\frac{\pi }{\mu }}\right|}^{r}}{c_{\mu }\left(s,t\right)}\leq 1 \end{array}\right\}.$$

Here $\left(\pi /\mu ,{\left(\pi /\mu \right)}^{\prime }\right)$ denotes the product of the two μ‐controlled paths $\left(\pi ,\pi^{\prime }\right)$ and $\left(\frac{1}{\mu },{\left(\frac{1}{\mu }\right)}^{\prime }\right)$ (see Lemma A.1). In particular, ${\left(\pi /\mu \right)}^{\prime }=\pi^{\prime }/\mu +\pi {\left(1/\mu \right)}^{\prime }$, and $R^{\frac{\pi }{\mu }}$ is the remainder of the controlled rough path π/μ.

Remark 4.2 We consider here controlled paths with respect to μ, instead of with respect to *S*. As noted in Remark 3.10, every *S*‐controlled path $\left(\pi ,\pi^{\prime }\right)\in V_{S}^{q}$ can be used to define the integral $\int \frac{\pi_{t}}{\mu_{t}}\;d\mu_{t}$, and all the results in this section can also be established based on $V_{S}^{q}$ with appropriate modifications. We choose to consider $\left(\pi ,\pi^{\prime }\right)\in V_{\mu }^{q}$ as a μ‐controlled path in order to slightly simplify the notation. It is straightforward to check that $V_{\mu }^{q}\subseteq V_{S}^{q}$.

Let us recall from Definition 2.3 that, for any T>0,

$$∥\left(Y,Y^{\prime }\right){∥}_{V_{\mu }^{q},\left[0,T\right]}=|Y_{0}\left|+\right|Y_{0}^{\prime }|+∥Y^{\prime }{∥}_{q,[0,T]}+{∥R^{Y}∥}_{r,[0,T]}$$

defines a complete norm on $V_{\mu }^{q}\left(\left[0,T\right];\Delta^{d}\right)$. We endow $A^{M,q}\left(c_{\mu }\right)\subset V_{\mu }^{q^{\prime }}\left(\left[0,\infty \right);\Delta^{d}\right)$ with the seminorms

(26) $$p_{T}^{\mu ,q^{\prime }}\left(\left(\pi ,\pi^{\prime }\right)\right):=∥\frac{\pi }{\mu },{\left(\frac{\pi }{\mu }\right)}^{\prime }{∥}_{V_{\mu }^{q^{\prime }},\left[0,T\right]},\;T>0.$$

The reason for taking $q^{\prime }>q$ is that it will allow us to obtain a compact embedding of $A^{M,q}\left(c_{\mu }\right)$ into $V_{\mu }^{q^{\prime }}$. This compactness of the set of admissible portfolios plays a crucial role in obtaining optimality of universal portfolios.

Let us discuss some examples of admissible portfolios. We first check that the functionally generated portfolios treated in Cuchiero et al. (2019) belong to $A^{M,q}\left(c_{\mu }\right)$ provided that the control function $c_{\mu }$ is chosen appropriately. Recall that $C^{k}\left({\bar{\Delta }}_{+}^{d};R_{+}\right)$ denotes the space of *k*‐times continuously differentiable $R_{+}$‐valued functions on the closed (non‐negative) simplex ${\bar{\Delta }}_{+}^{d}$, and that ${∥G∥}_{C^{k}}:=\max_{0\leq n\leq k}{∥D^{n}G∥}_{\infty }$.

Lemma 4.3 Let K>0 be a constant, and let

$$G^{K}=\left\{G\in C^{3}\left({\bar{\Delta }}_{+}^{d};R_{+}\right):{∥G∥}_{C^{3}}\leq K,\;G\geq \frac{1}{K}\right\}.$$

Then the portfolio π generated by *G*, as defined in Equation (21), belongs to $A^{M,p}\left(c_{\mu }\right)$ for a suitable control function $c_{\mu }$ and constant *M*. More precisely, there exists a control function of the form $c_{\mu }\left(\;\cdot \;,\;\cdot \;\right)=C{∥\mu ∥}_{p,[\;\cdot \;,\;\cdot \;]}^{p}$ and a constant M>0, such that *C* and *M* only depend on *K*, and

$$\{\left(\pi^{G},{\left(\pi^{G}\right)}^{\prime }\right):\pi^{G}\;\text{defined in Equation (21) for some}\;G\in G^{K}\}\subset A^{M,p}\left(c_{\mu }\right).$$

Note that here we take q=p and r=p/2.

Fix $G\in G^{K}$, and let π be the associated portfolio as defined in Equation (21). Since π is defined as a *C* ^2^ function of μ, we know immediately that it is a μ‐controlled path.

A simple calculation shows that

$$\frac{\pi_{t}}{\mu_{t}}=g_{t}+\left(1-\mu_{t}\cdot g_{t}\right)1,$$

where we write 1=(1,…,1) and $g_{t}=\nabla \log G\left(\mu_{t}\right)$, and we use · to denote the standard inner product on $R^{d}$. The pair (1,0) is trivially a μ‐controlled path with $1^{\prime }=0$ and $R^{1}=0$, and thus clearly satisfies the required bounds in Equation (25) with an arbitrary control function. It thus suffices to show that $\left(g,g^{\prime }\right)$ and $\left(\mu \cdot g,{\left(\mu \cdot g\right)}^{\prime }\right)$ satisfy the required bounds with control functions $c_{\mu }^{1}$ and $c_{\mu }^{2}$, respectively, since then $c_{\mu }:=c_{\mu }^{1}+c_{\mu }^{2}$ gives the desired control function.

We begin with $\left(g,g^{\prime }\right)$. Let F:=∇logG, so that g=F(μ) and $g^{\prime }=DF\left(\mu \right)$. By Taylor expansion, we can verify that, for all s≤t,

(27) $$|g_{s,t}{|\leq ∥DF∥}_{\infty }|\mu_{s,t}\left|,\;\right|g_{s,t}^{\prime }|\leq ∥D^{2}{F∥}_{\infty }|\mu_{s,t}\left|,\;\right|R_{s,t}^{g}|\leq ∥D^{2}{F∥}_{\infty }{\left|\mu_{s,t}\right|}^{2}.$$

Note that *F*, D*F*, and D^2^ *F* only depend on D*G*, D^2^ *G*, D^3^ *G*, and 1/G, and therefore, since ${∥G∥}_{C^{3}}\leq K$ and G≥1/K, there exists a constant C=C(K), which only depends on *K*, such that ${∥F∥}_{\infty }\leq C$, ${∥DF∥}_{\infty }\leq C$, and $∥D^{2}{F∥}_{\infty }\leq C$. It follows that we can choose $c_{\mu }^{1}\left(s,t\right)=C{∥\mu ∥}_{p,[s,t]}^{p}$. Note also that ${∥g∥}_{\infty }\leq C$ and $∥g^{\prime }{∥}_{\infty }\leq C$.

We now turn to $\left(\mu \cdot g,{\left(\mu \cdot g\right)}^{\prime }\right)$. Noting that μ is trivially a μ‐controlled path with $\mu^{\prime }=1$ and $R^{\mu }=0$, and that $R_{s,t}^{\mu \cdot g}=\mu_{s}\cdot R_{s,t}^{g}+\mu_{s,t}\cdot g_{s,t}$, we deduce that

$${|\left(\mu \cdot g\right)}_{s,t}^{\prime }\left|\leq \right|g_{s,t}{|+∥\mu ∥}_{\infty }|g_{s,t}^{\prime }|+∥g^{\prime }{∥}_{\infty }|\mu_{s,t}\left|,\;\right|R_{s,t}^{\mu \cdot g}{|\leq ∥\mu ∥}_{\infty }|R_{s,t}^{g}\left|+\right|\mu_{s,t}\left|\right|g_{s,t}|.$$

Since $\mu_{t}$ takes values in the bounded set $\Delta_{+}^{d}$, we can use the bounds in Equation (27) to show that there exists a constant L=L(K), depending only on *K*, such that ${|\left(\mu \cdot g\right)}_{s,t}^{\prime }\left|\leq L\right|\mu_{s,t}|$ and $|R_{s,t}^{\mu \cdot g}\left|\leq L\right|\mu_{s,t}{|}^{2}$. It follows that we may take $c_{\mu }^{2}\left(s,t\right):=L{∥\mu ∥}_{p,[s,t]}^{p}$. Finally, we note that the initial values $\pi_{0}/\mu_{0}=g_{0}+\left(1-\mu_{0}\cdot g_{0}\right)1=F\left(\mu_{0}\right)+\left(1-\mu_{0}\cdot F\left(\mu_{0}\right)\right)1$ and ${\left(\pi /\mu \right)}_{0}^{\prime }=DF\left(\mu_{0}\right)-\left(F\left(\mu_{0}\right)+\mu_{0}DF\left(\mu_{0}\right)\right)1$ are also bounded by a constant *M* depending only on *K*.□

One particular advantage of rough integration is that the admissible strategies need not be of gradient type, giving us more flexibility in choosing admissible portfolios compared to previous approaches relying on Föllmer integration.

Example 4.4 (Functionally controlled portfolios) Let

$$F^{2,K}:=\left\{\left(\pi^{F},\pi^{F,\prime }\right):F\in C^{2}\left({\bar{\Delta }}_{+}^{d};R^{d}\right),\;{∥F∥}_{C^{2}}\leq K\right\}$$

for a given constant K>0, where

(28) $$\begin{array}{c} {\left(\pi_{t}^{F}\right)}^{i}=\mu_{t}^{i}\left(F^{i}\left(\mu_{t}\right)+1-\sum_{j=1}^{d}\mu_{t}^{j}F^{j}\left(\mu_{t}\right)\right) \end{array}$$

for t≥0 and i=1,…,d. Then $F^{2,K}\subset A^{M,p}\left(c_{\mu }\right)$, where we can again take q=p. The point here is that we can consider all *C* ^2^‐functions *F*, rather than requiring that *F* is of the form F=∇logG for some function *G*. One can verify that $F^{2,K}\subset A^{M,p}\left(c_{\mu }\right)$ for a suitable control function $c_{\mu }$ by following the proof of Lemma 4.3 almost verbatim.

Example 4.5 (Controlled equation generated portfolios) Let us define

$$C^{3,K}:=\{f\in C^{3}\left(R^{d};L\left(R^{d};R^{d}\right)\right){:∥f∥}_{C^{3}}\leq K\}.$$

For a given $f\in C^{3,K}$, a classical result in rough path theory is that the controlled differential equation with the vector field *f*, driven by μ,

(29) $$dY_{t}^{f}=f\left(Y_{t}^{f}\right)\;d\mu_{t},\;Y_{0}=\xi \in \Delta^{d},$$

admits a unique solution $\left(Y^{f},{\left(Y^{f}\right)}^{\prime }\right)=\left(\xi +\int_{0}^{\cdot }f\left(Y_{u}^{f}\right)\;d\mu_{u},f\left(Y^{f}\right)\right)$, which is itself a μ‐controlled path. Moreover, writing $A_{s,t}^{\mu }=\int_{s}^{t}\mu_{s,u}⊗d\mu_{u}$ for the canonical rough path lift of μ (see Section A.3), and $c_{\mu }\left(s,t\right):={∥\mu ∥}_{p,[s,t]}^{p}+{∥A^{\mu }∥}_{\frac{p}{2},\left[s,t\right]}^{\frac{p}{2}}$, for every T>0, there exists a constant $\Gamma_{T}$ depending on *p*, $c_{\mu }\left(\left[0,T\right]\right)$ and *K*, such that

$$\underset{\left(s,t\right)\in \Delta_{\left[0,T\right]}}{\sup}\frac{{\left|{\left(Y^{f}\right)}_{s,t}^{\prime }\right|}^{p}}{\Gamma_{T}c_{\mu }\left(s,t\right)}+\underset{\left(s,t\right)\in \Delta_{\left[0,T\right]}}{\sup}\frac{{\left|R_{s,t}^{Y^{f}}\right|}^{\frac{p}{2}}}{\Gamma_{T}c_{\mu }\left(s,t\right)}\leq 1.$$

Consequently, as in the proof of Lemma 4.3, one can show that there exists an increasing function $\Gamma :\left[0,\infty \right)\to R_{+}$, depending on *p*, $c_{\mu }$, and *K* such that

$$\underset{0\leq s\leq t<\infty }{\sup}\frac{{\left|{\left(\frac{\pi^{f}}{\mu }\right)}_{s,t}^{\prime }\right|}^{p}}{{\tilde{c}}_{\mu }\left(s,t\right)}+\underset{0\leq s\leq t<\infty }{\sup}\frac{{\left|R_{s,t}^{\frac{\pi^{f}}{\mu }}\right|}^{\frac{p}{2}}}{{\tilde{c}}_{\mu }\left(s,t\right)}\leq 1,$$

where $\pi^{f}:=\mu \left(Y^{f}+\left(1-\mu \cdot Y^{f}\right)1\right)$ and ${\tilde{c}}_{\mu }\left(s,t\right):=\Gamma_{t}c_{\mu }\left(s,t\right)$ is again a control function. This implies that the set

$$\{\pi^{f}=\mu \left(Y^{f}+\left(1-\mu \cdot Y^{f}\right)1\right):Y^{f}\;\text{is the solution of Equation (29) for some}\;f\in C^{3,K}\}\subset A^{M,p}\left({\tilde{c}}_{\mu }\right)$$

for a suitable constant M>0.

### 4.2 Asymptotic growth of universal portfolios

To investigate the asymptotic growth rates of our pathwise versions of Cover's universal portfolio, we first require some auxiliary results—in particular the compactness of the set of admissible portfolios.

Lemma 4.6 The set $A^{M,q}\left(c_{\mu }\right)$ is compact in the topology generated by the family of seminorms $\left\{p_{T}^{\mu ,q^{\prime }}:T\in N\right\}$ as defined in Equation (26), where we recall that $q<q^{\prime }$.

*Step 1*: We first show that the set

$$A:=\left\{\left(Y,Y^{\prime }\right)\in V_{\mu }^{q}\left(\left[0,\infty \right);R^{d}\right)\;:\;|Y_{0}\left|+\right|Y_{0}^{\prime }|\leq M\;\text{and}\;\underset{s\leq t}{\sup}\frac{{\left|Y_{s,t}^{\prime }\right|}^{q}}{c_{\mu }\left(s,t\right)}+\underset{s\leq t}{\sup}\frac{{\left|R_{s,t}^{Y}\right|}^{r}}{c_{\mu }\left(s,t\right)}\leq 1\right\}$$

is compact with respect to the topology generated by the seminorms ${∥\;\cdot \;,\;\cdot \;∥}_{V_{\mu }^{q^{\prime }},\left[0,T\right]}$ for T∈N. It suffices to show that for every fixed T∈N, the set

AT:=|Ys,t′|qcμ(s,t)(Y,Y′)∈Vμq[0,T];Rd:|Y0|+Y0′≤Mandsup(s,t)∈Δ[0,T]|Ys,t′|qcμ(s,t)+sup(s,t)∈Δ[0,T]|Rs,tY|rcμ(s,t)≤1

is compact with respect to the norm ${∥\;\cdot \;,\;\cdot \;∥}_{V_{\mu }^{q^{\prime }},\left[0,T\right]}$. We first note that, for all $\left(Y,Y^{\prime }\right)\in A_{T}$,

$$∥Y^{\prime }{∥}_{q,[0,T]}\leq c_{\mu }{\left(0,T\right)}^{\frac{1}{q}},\;∥Y^{\prime }{∥}_{\infty ,[0,T]}\leq M+c_{\mu }{\left(0,T\right)}^{\frac{1}{q}}\;\text{and}\;{∥R^{Y}∥}_{r,[0,T]}\leq c_{\mu }{\left(0,T\right)}^{\frac{1}{r}},$$

where the second bound follows from the fact that $|Y_{t}^{\prime }\left|\leq \right|Y_{0}^{\prime }\left|+\right|Y_{0,t}^{\prime }|\leq M+∥Y^{\prime }{∥}_{q,[0,T]}$. The *p*‐variation of *Y* can also be controlled as follows. From $Y_{s,t}=Y_{s}^{\prime }\mu_{s,t}+R_{s,t}^{Y}$, we have

$$|Y_{s,t}{|}^{p}\leq 2^{p-1}\left(∥Y^{\prime }{∥}_{\infty ,[0,T]}^{p}{\left|\mu_{s,t}\right|}^{p}+{\left|R_{s,t}^{Y}\right|}^{p}\right),\;\left(s,t\right)\in \Delta_{\left[0,T\right]},$$

and hence

$${∥Y∥}_{p,[0,T]}\leq 2^{\frac{p-1}{p}}\left(∥Y^{\prime }{∥}_{\infty ,[0,T]}{∥\mu ∥}_{p,[0,T]}+{∥R^{Y}∥}_{p,[0,T]}\right)\leq 2^{\frac{p-1}{p}}\left(∥Y^{\prime }{∥}_{\infty ,[0,T]}{∥\mu ∥}_{p,[0,T]}+{∥R^{Y}∥}_{r,[0,T]}\right),$$

since r<p (see, e.g., Chistyakov and Galkin (1998, Remark 2.5)), and thus

$${∥Y∥}_{\infty ,[0,T]}\leq M+{∥Y∥}_{p,[0,T]}\leq M+2^{\frac{p-1}{p}}\left(\left(M+c_{\mu }{\left(0,T\right)}^{\frac{1}{q}}\right){∥\mu ∥}_{p,[0,T]}+c_{\mu }{\left(0,T\right)}^{\frac{1}{r}}\right).$$

Therefore, by Friz and Victoir (2010, Proposition 5.28), every sequence ${\left(Y^{n},Y^{n,\prime }\right)}_{n\geq 1}\subset A_{T}$ has a convergent subsequence, which we still denote by ${\left(Y^{n},Y^{n,\prime }\right)}_{n\geq 1}$, and limits $Y\in C^{p\text{-var}}\left(\left[0,T\right];R^{d}\right)$ and $Y^{\prime }\in C^{q\text{-var}}\left(\left[0,T\right];R^{d}\right)$, such that $|Y_{0}^{n}-Y_{0}|+∥Y^{n}{-Y∥}_{p^{\prime },\left[0,T\right]}\to 0$ and $|Y_{0}^{n,\prime }-Y_{0}^{\prime }|+∥Y^{n,\prime }-Y^{\prime }{∥}_{q^{\prime },\left[0,T\right]}\to 0$, respectively, as n→∞, for an arbitrary $p^{\prime }>p$. Since

$$\begin{array}{cc} \left|R_{s,t}^{Y^{n}}-R_{s,t}^{Y^{n+1}}\right| & \leq \left|Y_{s}^{n,\prime }\mu_{s,t}-Y_{s}^{n+1,\prime }\mu_{s,t}\right|+\left|Y_{s,t}^{n}-Y_{s,t}^{n+1}\right|\\ & \leq \left|Y_{s}^{n,\prime }-Y_{s}^{n+1,\prime }\right|\left|\mu_{s,t}\right|+\left|Y_{t}^{n}-Y_{t}^{n+1}\right|+\left|Y_{s}^{n}-Y_{s}^{n+1}\right|\;⟶\;0 \end{array}$$

as n→∞, uniformly in $\left(s,t\right)\in \Delta_{\left[0,T\right]}$, we have that

$$\begin{array}{cc} {\left\|R^{Y^{n}}-R^{Y^{n+1}}\right\|}_{r^{\prime },\left[0,T\right]} & \leq {\left\|R^{Y^{n}}-R^{Y^{n+1}}\right\|}_{r,[0,T]}^{\frac{r}{r^{\prime }}}\underset{\left(s,t\right)\in \Delta_{\left[0,T\right]}}{\sup}{\left|R_{s,t}^{Y^{n}}-R_{s,t}^{Y^{n+1}}\right|}^{\frac{r^{\prime }-r}{r^{\prime }}}\\ & \leq 2^{\frac{r}{r^{\prime }}}c_{\mu }{\left(0,T\right)}^{\frac{1}{r^{\prime }}}\underset{\left(s,t\right)\in \Delta_{\left[0,T\right]}}{\sup}{\left|R_{s,t}^{Y^{n}}-R_{s,t}^{Y^{n+1}}\right|}^{\frac{r^{\prime }-r}{r^{\prime }}}\;⟶\;0 \end{array}$$

as n→∞. Thus, $R^{Y^{n}}$ also converges to some $R^{Y}$ in $r^{\prime }$‐variation.

To see that the limit $\left(Y,Y^{\prime }\right)\in A_{T}$, we simply note that

$$\begin{array}{cc} \frac{{\left|Y_{s,t}^{\prime }\right|}^{q}}{c_{\mu }\left(s,t\right)}+\frac{{\left|R_{u,v}^{Y}\right|}^{r}}{c_{\mu }\left(u,v\right)} & =\lim_{n\to \infty }\left(\frac{{\left|Y_{s,t}^{n,\prime }\right|}^{q}}{c_{\mu }\left(s,t\right)}+\frac{{\left|R_{u,v}^{Y^{n}}\right|}^{r}}{c_{\mu }\left(u,v\right)}\right)\leq 1, \end{array}$$

and then take the supremum over $\left(s,t\right)\in \Delta_{\left[0,T\right]}$ and $\left(u,v\right)\in \Delta_{\left[0,T\right]}$ on the left‐hand side.

Thus, $A_{T}$ is compact with respect to $p_{T}^{\mu ,q^{\prime }}$, and A is then compact in the topology generated by the seminorms $p_{T}^{\mu ,q^{\prime }}$ for T∈N.

*Step 2*: Now suppose that ${\left\{\left(\pi^{n},\pi^{n,\prime }\right)\right\}}_{n\in N}$ is a sequence of portfolios in $A^{M,q}\left(c_{\mu }\right)$. Correspondingly, ${\left\{\left(\frac{\pi^{n}}{\mu },{\left(\frac{\pi^{n}}{\mu }\right)}^{\prime }\right)\right\}}_{n\in N}$ is then a sequence in A which, by the result in Step 1 above, admits a convergent subsequence with respect to the seminorms ${∥\;\cdot \;,\;\cdot \;∥}_{V_{\mu }^{q^{\prime }},\left[0,T\right]}$ for T∈N. Since $∥\frac{\pi }{\mu },{\left(\frac{\pi }{\mu }\right)}^{\prime }{∥}_{V_{\mu }^{q^{\prime }},\left[0,T\right]}=p_{T}^{\mu ,q^{\prime }}\left(\left(\pi ,\pi^{\prime }\right)\right)$, the convergence also applies to the corresponding subsequence of ${\left\{\left(\pi^{n},\pi^{n,\prime }\right)\right\}}_{n\in N}$ with respect to the seminorms ${\left\{p_{T}^{\mu ,q^{\prime }}\right\}}_{T\in N}$. Let $\left(\phi ,\phi^{\prime }\right)$ be the limit of (the convergent subsequence of) ${\left\{\left(\frac{\pi^{n}}{\mu },{\left(\frac{\pi^{n}}{\mu }\right)}^{\prime }\right)\right\}}_{n\in N}$. It is then easy to see that ϕμ, the product of controlled paths $\left(\phi ,\phi^{\prime }\right)$ and (μ,I), is a cluster point of ${\left\{\left(\pi^{n},\pi^{n,\prime }\right)\right\}}_{n\in N}$ in $A^{M,q}\left(c_{\mu }\right)$ with respect to the seminorms ${\left\{p_{T}^{\mu ,q^{\prime }}\right\}}_{T\in N}$.□

In the next auxiliary result, we establish continuity of the relative wealth of admissible portfolios with respect to the market portfolio. To this end, we recall the family of seminorms ${\left\{p_{T}^{\mu ,q^{\prime }}\right\}}_{T>0}$, defined in Equation (26), and, for a given sequence $\beta ={\left\{\beta_{N}\right\}}_{N\in N}$ with $\beta_{N}>0$ for all N∈N and $\lim_{N\to \infty }\beta_{N}=\infty$, we introduce a metric $d_{\beta }$ on $A^{M,q}\left(c_{\mu }\right)$ via

$$d_{\beta }\left(\left(\pi ,\pi^{\prime }\right),\left(\phi ,\phi^{\prime }\right)\right):=\underset{N\geq 1}{\sup}\frac{1}{\beta_{N}\gamma_{N}}p_{N}^{\mu ,q^{\prime }}\left(\left(\pi ,\pi^{\prime }\right)-\left(\phi ,\phi^{\prime }\right)\right),$$

where

$$\gamma_{N}:=1+M+c_{\mu }{\left(0,N\right)}^{\frac{1}{q}}+c_{\mu }{\left(0,N\right)}^{\frac{1}{r}}.$$

Since $p_{N}^{\mu ,q^{\prime }}\left(\left(\pi ,\pi^{\prime }\right)\right)\leq \gamma_{N}$, we have that $d_{\beta }\left(\left(\pi ,\pi^{\prime }\right),\left(\phi ,\phi^{\prime }\right)\right)<\infty$ for all portfolios $\left(\pi ,\pi^{\prime }\right),\left(\phi ,\phi^{\prime }\right)\in A^{M,q}\left(c_{\mu }\right)$. The metric $d_{\beta }$ is thus well‐defined on $A^{M,q}\left(c_{\mu }\right)$. Moreover, it is not hard to see that the topology induced by the metric $d_{\beta }$ coincides with the topology generated by the family of seminorms ${\left\{p_{T}^{\mu ,q^{\prime }}\right\}}_{T\in N}$, so that $\left(A^{M,q}\left(c_{\mu }\right),d_{\beta }\right)$ is a compact metric space. For T>0, we also denote

(30) $$\xi_{T}:={∥\mu ∥}_{p,[0,T]}+{∥A^{\mu }∥}_{\frac{p}{2},\left[0,T\right]}+\sum_{i=1}^{d}{\left[\mu \right]}_{T}^{ii}.$$

Lemma 4.7 For any T>0, we have that the estimate

(31) $$|\log V_{T}^{\pi }-\log V_{T}^{\phi }|\leq C\beta_{N}\gamma_{N}^{2}\xi_{T}d_{\beta }\left(\left(\pi ,\pi^{\prime }\right),\left(\phi ,\phi^{\prime }\right)\right)$$

holds for all $\left(\pi ,\pi^{\prime }\right),\left(\phi ,\phi^{\prime }\right)\in A^{M,q}\left(c_{\mu }\right)$, for some constant *C*, which depends only on $p,q^{\prime },r^{\prime }$ and the dimension *d*, where N=⌈T⌉, and $V^{\pi }$ denotes the relative wealth process as defined in Equation (14). In particular, the map from $A^{M,q}\left(c_{\mu }\right)\to R$ given by $\left(\pi ,\pi^{\prime }\right)\mapsto V_{T}^{\pi }$ is continuous with respect to the metric $d_{\beta }$.

By Proposition 3.9 and the relation in Equation (24), we have that, for any $\left(\pi ,\pi^{\prime }\right)\in A^{M,q}\left(c_{\mu }\right)$,

$$\log V_{T}^{\pi }=\int_{0}^{T}\frac{\pi_{s}}{\mu_{s}}\;d\mu_{s}-\frac{1}{2}\sum_{i,j=1}^{d}\int_{0}^{T}\frac{\pi_{s}^{i}\pi_{s}^{j}}{\mu_{s}^{i}\mu_{s}^{j}}\;d{\left[\mu \right]}_{s}^{ij},$$

which implies that, for $\left(\pi ,\pi^{\prime }\right),\left(\phi ,\phi^{\prime }\right)\in A^{M,q}\left(c_{\mu }\right)$,

$$\left|\log V_{T}^{\pi }-\log V_{T}^{\phi }\right|\leq \left|\int_{0}^{T}\frac{\pi_{s}-\phi_{s}}{\mu_{s}}\;d\mu_{s}\right|+\frac{1}{2}\left|\sum_{i,j=1}^{d}\int_{0}^{T}\frac{\left(\pi_{s}^{i}-\phi_{s}^{i}\right)\left(\pi_{s}^{j}+\phi_{s}^{j}\right)}{\mu_{s}^{i}\mu_{s}^{j}}\;d{\left[\mu \right]}_{s}^{ij}\right|.$$

We aim to bound the two terms on the right‐hand side. Let $A^{\mu }$ be the canonical rough path lift of μ (as defined in Section A.3), namely $A_{s,t}^{\mu }=\int_{s}^{t}\mu_{s,u}⊗d\mu_{u}$. Writing N=⌈T⌉, by the estimate for rough integrals in Equation (4), we obtain

$$\begin{array}{cc} \left|\int_{0}^{T}\frac{\pi_{s}-\phi_{s}}{\mu_{s}}\;d\mu_{s}\right| & ≲{\left\|R^{\frac{\pi -\phi }{\mu }}\right\|}_{r^{\prime },\left[0,T\right]}{∥\mu ∥}_{p,[0,T]}+{\left\|{\left(\frac{\pi -\phi }{\mu }\right)}^{\prime }\right\|}_{q^{\prime },\left[0,T\right]}{∥A^{\mu }∥}_{\frac{p}{2},\left[0,T\right]}\\ & \;+\left|\frac{\pi_{0}-\phi_{0}}{\mu_{0}}\right|{∥\mu ∥}_{p,[0,T]}+\left|{\left(\frac{\pi -\phi }{\mu }\right)}_{0}^{\prime }\right|{∥A^{\mu }∥}_{\frac{p}{2},\left[0,T\right]}\\ & ≲p_{N}^{\mu ,q^{\prime }}\left(\left(\pi ,\pi^{\prime }\right)-\left(\phi ,\phi^{\prime }\right)\right)\left({∥\mu ∥}_{p,[0,T]}+{∥A^{\mu }∥}_{\frac{p}{2},\left[0,T\right]}\right)\\ & \leq \beta_{N}\gamma_{N}d_{\beta }\left(\left(\pi ,\pi^{\prime }\right),\left(\phi ,\phi^{\prime }\right)\right)\left({∥\mu ∥}_{p,[0,T]}+{∥A^{\mu }∥}_{\frac{p}{2},\left[0,T\right]}\right). \end{array}$$

For the second term, we note that

(32) $$|\int_{0}^{T}\frac{\left(\pi_{s}^{i}-\phi_{s}^{i}\right)\left(\pi_{s}^{j}+\phi_{s}^{j}\right)}{\mu_{s}^{i}\mu_{s}^{j}}\;d{\left[\mu \right]}_{s}^{ij}|≲∥\frac{\pi -\phi }{\mu }{∥}_{\infty ,[0,T]}∥\frac{\pi +\phi }{\mu }{∥}_{\infty ,[0,T]}\sum_{i=1}^{d}{\left[\mu \right]}_{T}^{ii}.$$

It follows from the relation $\frac{\pi_{t}}{\mu_{t}}=\frac{\pi_{0}}{\mu_{0}}+{\left(\frac{\pi }{\mu }\right)}_{0}^{\prime }\mu_{0,t}+R_{0,t}^{\frac{\pi }{\mu }}$, and the fact that μ takes values in the bounded set $\Delta_{+}^{d}$, that

$$∥\frac{\pi }{\mu }{∥}_{\infty ,[0,T]}≲M+c_{\mu }{\left(0,T\right)}^{\frac{1}{r}}\leq \gamma_{N}.$$

It follows similarly from $\frac{\pi_{t}-\phi_{t}}{\mu_{t}}=\frac{\pi_{0}-\phi_{0}}{\mu_{0}}+{\left(\frac{\pi -\phi }{\mu }\right)}_{0}^{\prime }\mu_{0,t}+R_{0,t}^{\frac{\pi -\phi }{\mu }}$, that

$$∥\frac{\pi -\phi }{\mu }{∥}_{\infty ,[0,T]}≲p_{N}^{\mu ,q^{\prime }}\left(\left(\pi ,\pi^{\prime }\right)-\left(\phi ,\phi^{\prime }\right)\right)\leq \beta_{N}\gamma_{N}d_{\beta }\left(\left(\pi ,\pi^{\prime }\right),\left(\phi ,\phi^{\prime }\right)\right).$$

Substituting back into Equation (32), we obtain

$$\left|\int_{0}^{T}\frac{\left(\pi_{s}^{i}-\phi_{s}^{i}\right)\left(\pi_{s}^{j}+\phi_{s}^{j}\right)}{\mu_{s}^{i}\mu_{s}^{j}}\;d{\left[\mu \right]}_{s}^{ij}\right|≲\beta_{N}\gamma_{N}^{2}d_{\beta }\left(\left(\pi ,\pi^{\prime }\right),\left(\phi ,\phi^{\prime }\right)\right)\sum_{i=1}^{d}{\left[\mu \right]}_{T}^{ii}.$$

Combining the inequalities above, we deduce the desired estimate.□

In the following, we will sometimes write simply $A^{M,q}:=A^{M,q}\left(c_{\mu }\right)$ for brevity.

For $\left(\pi ,\pi^{\prime }\right)\in A^{M,q}$, we have by definition that π is a μ‐controlled path. We also have that the relative wealth $V^{\pi }$ is also a μ‐controlled rough path—as can be seen for instance from Proposition 3.9—and hence the product $\pi V^{\pi }$ is also a controlled path. Let ν be a fixed probability measure on $\left(A^{M,q},d_{\beta }\right)$. Observe that for every T>0, the space $V_{\mu }^{q}\left(\left[0,T\right];R^{d}\right)$ of controlled paths is a Banach space, and that, as we will see during the proof of Lemma 4.8 below, $V^{\pi }$ is the unique solution to the rough differential equation (34), which implies that the mapping $\pi \mapsto V^{\pi }{|}_{\left[0,T\right]}\in V_{\mu }^{q}\left(\left[0,T\right];R^{d}\right)$ is continuous by the continuity of the Itô–Lyons map (see, e.g., Lejay (2012, Theorem 1)). Hence, for every T>0, we can define the Bochner integral $\int_{A^{M,q}}\left(\pi V^{\pi }\right){|}_{\left[0,T\right]}\;d\nu \left(\pi \right)$, which is thus itself another controlled path defined on [0, *T*]. The μ‐controlled path

(33) $$\pi_{t}^{\nu }:=\frac{\int_{A^{M,q}}\pi_{t}V_{t}^{\pi }\;d\nu \left(\pi \right)}{\int_{A^{M,q}}V_{t}^{\pi }\;d\nu \left(\pi \right)},\;t\in \left[0,\infty \right),$$

is then well‐defined, and defines indeed a portfolio in $V_{\mu }^{q}$, called the *universal portfolio* associated to the set $A^{M,q}$ of admissible portfolios.

Lemma 4.8 Let $\pi^{\nu }$ be the universal portfolio as defined in Equation (33). Then, for all T>0,

$$V_{T}^{\pi^{\nu }}=\int_{A^{M,q}}V_{T}^{\pi }\;d\nu \left(\pi \right).$$

By Proposition 3.9 and the relation in Equation (24), we have, for any portfolio π,

$$V_{t}^{\pi }=\exp\left(\int_{0}^{t}\frac{\pi_{s}}{\mu_{s}}\;d\mu_{s}-\frac{1}{2}\sum_{i,j=1}^{d}\int_{0}^{t}\frac{\pi_{s}^{i}\pi_{s}^{j}}{\mu_{s}^{i}\mu_{s}^{j}}\;d{\left[\mu \right]}_{s}^{ij}\right).$$

Setting $Z:=\int_{0}^{\cdot }\frac{\pi_{s}}{\mu_{s}}\;d\mu_{s}$, by Lemma B.1, we can rewrite the relation above as $V^{\pi }=\exp\left(Z-\frac{1}{2}\left[Z\right]\right)$. Thus, by Lemma A.5, Lemma A.4, and Proposition A.2, we deduce that $V^{\pi }$ is the unique solution *Y* to the linear rough differential equation

(34) $$Y_{t}=1+\int_{0}^{t}Y_{s}\frac{\pi_{s}}{\mu_{s}}\;d\mu_{s},\;t\geq 0.$$

It is, therefore, sufficient to show that the path $t\mapsto \int_{A^{M,q}}V_{t}^{\pi }\;d\nu \left(\pi \right)$ also satisfies the RDE (34) with π replaced by $\pi^{\nu }$. By the definition of the universal portfolio in Equation (33), we have

(35) $$\int_{A^{M,q}}V_{s}^{\pi }\;d\nu \left(\pi \right)\frac{\pi_{s}^{\nu }}{\mu_{s}}=\int_{A^{M,q}}\frac{\pi_{s}}{\mu_{s}}V_{s}^{\pi }\;d\nu \left(\pi \right).$$

Recalling that $V^{\pi }$ satisfies Equation (34), we know that

$$V_{t}^{\pi }=1+\int_{0}^{t}\frac{\pi_{s}}{\mu_{s}}V_{s}^{\pi }\;d\mu_{s}.$$

By the Fubini theorem for rough integration (Theorem A.6), we then have that

$$\begin{array}{cc} \int_{A^{M,q}}V_{t}^{\pi }\;d\nu \left(\pi \right) & =1+\int_{0}^{t}\int_{A^{M,q}}\frac{\pi_{s}}{\mu_{s}}V_{s}^{\pi }\;d\nu \left(\pi \right)\;d\mu_{s}=1+\int_{0}^{t}\int_{A^{M,q}}V_{s}^{\pi }\;d\nu \left(\pi \right)\frac{\pi_{s}^{\nu }}{\mu_{s}}\;d\mu_{s}, \end{array}$$

where we used Equation (35) to obtain the last equality. Hence, both $V^{\pi^{\nu }}$ and $\int_{A^{M,q}}V^{\pi }\;d\nu \left(\pi \right)$ are the unique solution of the same RDE, and thus coincide.□

With these preparations in place, we now aim to compare the growth rates of the universal portfolio (33) and the best retrospectively chosen portfolio. For this purpose, we fix an M>0, and assume that there exists a compact metric space $\left(K,d_{K}\right)$ together with a mapping $\iota :\left(K,d_{K}\right)\to \left(A^{M,q},d_{\beta }\right)$ such that ι is continuous and injective (and thus a homeomorphism onto its image), and that for every T>0 and x,y∈K, we have that

(36) $$\left|\log V_{T}^{\iota (x)}-\log V_{T}^{\iota (y)}\right|\leq C\lambda \left(T\right)d_{K}\left(x,y\right),$$

where λ is a positive function of *T*, and *C* is a universal constant independent of *T*. Here we list some examples of $\left(K,d_{K}\right)$, ι, and λ:

(1) $K=C^{p+\alpha ,K}\left({\bar{\Delta }}_{+}^{d};R^{d}\right)=\{G\in C^{p+\alpha }\left({\bar{\Delta }}_{+}^{d};R^{d}\right){:∥G∥}_{C^{p+\alpha }}\leq K,\;G\geq \frac{1}{K}\}$, $d_{K}\left(G,\tilde{G}\right)={∥G-\tilde{G}∥}_{C^{2}}$, $\iota \left(G\right)=\pi^{G}$, where α>0 and $\pi^{G}$ is a classical functionally generated portfolio of the form (21). In this case, we can take $\lambda \left(T\right)=1+\max_{i=1,\text{…},d}{\left[\mu \right]}_{T}^{ii}$; see the proof of Cuchiero et al. (2019, Lemma 4.4).
(2) $K=C^{2+\alpha ,K}\left({\bar{\Delta }}_{+}^{d};R^{d}\right)=\{F\in C^{2+\alpha }\left({\bar{\Delta }}_{+}^{d};R^{d}\right){:∥F∥}_{C^{2+\alpha }}\leq K\}$, $d_{K}\left(F,\tilde{F}\right)={∥F-\tilde{F}∥}_{C^{2}}$, $\iota \left(F\right)=\pi^{F}$, where α∈(0,1] and $\pi^{F}$ is a functionally controlled portfolio defined as in Equation (28). In this case one may take $\lambda \left(T\right)={(1+∥\mu ∥}_{p,[0,T]}^{2})\xi_{T}$, where $\xi_{T}$ is defined in Equation (30); see Lemma 4.11 below.
(3) $K=A^{M,q}$, $d_{K}=d_{\beta }$, $\iota ={\text{Id}}_{A^{M,q}}$. In view of Equation (31), we have $\lambda \left(T\right)=\beta_{\left\lceil T\right\rceil }\gamma_{\left\lceil T\right\rceil }^{2}\xi_{T}$.

Given such a compact space $\left(K,d_{K}\right)$ equipped with an embedding ι as above, we define

$$V_{T}^{*,K,\iota }=\underset{x\in K}{\sup}V_{T}^{\iota (x)}=\underset{\pi \in \iota (K)}{\sup}V_{T}^{\pi }.$$

By the compactness of K and the continuity provided by the estimate in Equation (36), we have that, for each T>0, there exists a portfolio $\pi^{*,T}\in \iota \left(K\right)$, which can be expressed as $\pi^{*,T}=\iota \left(x^{*}\right)$ for some $x^{*}\in K$, known as the *best retrospectively chosen portfolio* associated with K and ι, such that

(37) $$V_{T}^{*,K,\iota }=V^{\pi^{*,T}}.$$

The following theorem provides an analog of Cuchiero et al. (2019, Theorem 4.11) in our rough path setting.

Theorem 4.9 Let $\left(K,d_{K}\right)$ be a compact metric space equipped with a continuous embedding $\iota :\left(K,d_{K}\right)\to \left(A^{M,q},d_{\beta }\right)$, which satisfies the bound in Equation (36) for some positive function λ. Let *m* be a probability measure on K with full support, and let $\nu =\iota_{*}\left(m\right)$ denote the pushforward measure on $A^{M,q}$. If $\lim_{T\to \infty }\lambda \left(T\right)=\infty$, then

$$\lim_{T\to \infty }\frac{1}{\lambda (T)}\left(\log V_{T}^{*,K,\iota }-\log V_{T}^{\pi^{\nu }}\right)=0.$$

In particular, if $K=C^{p+\alpha ,K}\left({\bar{\Delta }}_{+}^{d};R^{d}\right)=\{G\in C^{p+\alpha }\left({\bar{\Delta }}_{+}^{d};R^{d}\right){:∥G∥}_{C^{p+\alpha }}\leq K,\;G\geq \frac{1}{K}\}$, $d_{K}\left(G,\tilde{G}\right)={∥G-\tilde{G}∥}_{C^{2}}$, $\iota \left(G\right)=\pi^{G}$, where $\pi^{G}$ is a classical functionally generated portfolio of the form (21), and $\lambda \left(T\right)=1+\max_{i=1,\text{…},d}{\left[\mu \right]}_{T}^{ii}$, then one also infers the version of Cover's theorem obtained in Cuchiero et al. (2019, Theorem 4.11).

Proof of Theorem 4.9 As the inequality “≥” is trivial, we need only show the reverse inequality. As K is compact and *m* has full support, we have that, for any η∈(0,1), there exists a δ>0 such that every η‐ball around a point x∈K with respect to $d_{K}$ has *m*‐measure bigger than δ.

Let T>0 be such that λ(T)≥1, and let $\pi^{*,T}=\iota \left(x^{*}\right)$ be the best retrospectively chosen portfolio, as in Equation (37). For any portfolio $\pi =\iota \left(x\right)\in \iota \left(K\right)\subseteq A^{M,q}\left(c_{\mu }\right)$ such that $d_{K}\left(x,x^{*}\right)\leq \eta$, the estimate in Equation (36) implies that

$$\frac{1}{\lambda (T)}\left(\log V_{T}^{\pi }-\log V_{T}^{\pi^{*,T}}\right)\geq -Cd_{K}\left(x,x^{*}\right)\geq -C\eta ,$$

for some constant *C*. For any ε>0, we can, therefore, choose η small enough such that

(38) $$\frac{1}{\lambda (T)}\left(\log V_{T}^{\pi }-\log V_{T}^{\pi^{*,T}}\right)\geq -\varepsilon .$$

Let $B_{\eta }\left(x^{*}\right)$ denote the η‐ball in K around the point $x^{*}$ with respect to the metric $d_{K}$, which has *m*‐measure $|B_{\eta }\left(x^{*}\right)|\geq \delta$. By Lemma 4.8 and Jensen's inequality, we have that

$${\left(V_{T}^{\pi^{\nu }}\right)}^{\frac{1}{\lambda (T)}}\geq {\left(\int_{B_{\eta }\left(x^{*}\right)}V_{T}^{\iota (x)}\;dm\left(x\right)\right)}^{\frac{1}{\lambda (T)}}\geq {\left|B_{\eta }\left(x^{*}\right)\right|}^{\frac{1}{\lambda (T)}-1}\int_{B_{\eta }\left(x^{*}\right)}{\left(V_{T}^{\iota (x)}\right)}^{\frac{1}{\lambda (T)}}\;dm\left(x\right).$$

Then, using Equation (38), we have

$${\left(\frac{V_{T}^{\pi^{\nu }}}{V_{T}^{\pi^{*,T}}}\right)}^{\frac{1}{\lambda (T)}}\geq |B_{\eta }\left(x^{*}\right){|}^{\frac{1}{\lambda (T)}-1}\int_{B_{\eta }\left(x^{*}\right)}{\left(\frac{V_{T}^{\iota (x)}}{V_{T}^{\iota (x^{*})}}\right)}^{\frac{1}{\lambda (T)}}\;dm\left(x\right)\geq {\left|B_{\eta }\left(x^{*}\right)\right|}^{\frac{1}{\lambda (T)}}e^{-\varepsilon }\geq \delta^{\frac{1}{\lambda (T)}}e^{-\varepsilon }.$$

Taking ε>0 arbitrarily small (which determines η and hence also δ) and then T>0 sufficiently large, we deduce the desired inequality.□

### 4.3 Universal portfolios based on functionally controlled portfolios

The most frequently considered classes of portfolios are those which are generated by functions acting on the underlying price trajectories, such as the functionally generated portfolios in Lemma 4.3. In this section, we shall investigate the growth rate of universal portfolios based on the more general class of functionally controlled portfolios, as introduced in Example 4.4. More precisely, we fix constants α∈(0,1] and K>0, and consider the sets

$$C^{2+\alpha ,K}\left({\bar{\Delta }}_{+}^{d};R^{d}\right):=\left\{F\in C^{2+\alpha }\left({\bar{\Delta }}_{+}^{d};R^{d}\right):{∥F∥}_{C^{2+\alpha }}\leq K\right\}$$

and

$$F^{2+\alpha ,K}:=\{\left(\pi^{F},\pi^{F,\prime }\right):F\in C^{2+\alpha ,K}\left({\bar{\Delta }}_{+}^{d};R^{d}\right)\},$$

where the portfolio $\pi^{F}$ is of the form in Equation (28). Here we recall that $C^{2+\alpha }$ denotes the space of twice continuously differentiable functions whose second derivative is α‐Hölder continuous.

Lemma 4.10 For any T>0 and any $F,G\in C^{2+\alpha ,K}\left({\bar{\Delta }}_{+}^{d};R^{d}\right)$, we have that

(39) $$p_{T}^{\mu ,p}\left(\left(\pi^{F},\pi^{F,\prime }\right)-\left(\pi^{G},\pi^{G,\prime }\right)\right)\leq C{∥F-G∥}_{C^{2}}\left(1+{∥\mu ∥}_{p,[0,T]}^{2}\right),$$

where the constant *C* depends only on p,d, and *K*. Considering the map $\Phi :C^{2+\alpha ,K}\left({\bar{\Delta }}_{+}^{d};R^{d}\right)\to F^{2+\alpha ,K}$ given by^2^

$$F\;\mapsto \;\Phi \left(F\right):=\left(\pi^{F},\pi^{F,\prime }\right),$$

where $\pi^{F}$ is of the form in Equation (28), we thus have that Φ is continuous with respect to the *C* ^2^‐distance on $C^{2+\alpha ,K}\left({\bar{\Delta }}_{+}^{d};R^{d}\right)$ and each of the seminorms ${\left\{p_{T}^{\mu ,p}\right\}}_{T>0}$ on $F^{2+\alpha ,K}\subset A^{M,p}\left(c_{\mu }\right)$. As the notation suggests, here $p_{T}^{\mu ,p}$ is defined as in Equation (26) with $q^{\prime }$ replaced by *p*.

In the following, for notational simplicity, we will omit the Gubinelli derivative in the norms ${∥\;\cdot \;,\;\cdot \;∥}_{V_{\mu }^{p},\left[0,T\right]}$ and seminorms $p_{T}^{\mu ,p}\left(\left(\;\cdot \;,\;\cdot \;\right)\right)$; that is, we will write, for example, ${∥\pi ∥}_{V_{\mu }^{p},\left[0,T\right]}$ instead of $∥\pi ,\pi^{\prime }{∥}_{V_{\mu }^{p},\left[0,T\right]}$. Let $F,G\in C^{2+\alpha ,K}$ and s≤t. We have

$$\begin{array}{cc} |\left(DF-DG\right)\left(\mu_{t}\right)-\left(DF-DG\right)\left(\mu_{s}\right)| & =|\int_{0}^{1}\left(D^{2}F-D^{2}G\right)\left(\mu_{s}+\lambda \mu_{s,t}\right)\mu_{s,t}\;d\lambda |\\ & \leq ∥D^{2}F-D^{2}{G∥}_{\infty }\left|\mu_{s,t}\right|, \end{array}$$

so that

$${∥DF\left(\mu \right)-DG\left(\mu \right)∥}_{p,[0,T]}\leq {∥F-G∥}_{C^{2}}{∥\mu ∥}_{p,[0,T]}.$$

Similarly, since

$$R_{s,t}^{F(\mu )}=F\left(\mu_{t}\right)-F\left(\mu_{s}\right)-DF\left(\mu_{s}\right)\mu_{s,t}=\int_{0}^{1}\int_{0}^{1}D^{2}F\left(\mu_{s}+\lambda_{1}\lambda_{2}\mu_{s,t}\right)\mu_{s,t}^{⊗2}\lambda_{1}\;d\lambda_{2}\;d\lambda_{1},$$

we have

$$∥R^{F(\mu )}-R^{G(\mu )}{∥}_{\frac{p}{2},\left[0,T\right]}\leq {∥F-G∥}_{C^{2}}{∥\mu ∥}_{p,[0,T]}^{2}.$$

Thus, for μ‐controlled paths (F(μ),DF(μ)) and (G(μ),DG(μ)), we have that

(40) $${∥F\left(\mu \right)-G\left(\mu \right)∥}_{V_{\mu }^{p},\left[0,T\right]}≲{∥F-G∥}_{C^{2}}\left(1+{∥\mu ∥}_{p,[0,T]}^{2}\right).$$

Writing $\pi_{t}^{F}/\mu_{t}=F\left(\mu_{t}\right)+\left(1-\mu_{t}\cdot F\left(\mu_{t}\right)\right)1$ and $\pi_{t}^{G}/\mu_{t}=G\left(\mu_{t}\right)+\left(1-\mu_{t}\cdot G\left(\mu_{t}\right)\right)1$, we have that

$$\frac{\pi_{t}^{F}-\pi_{t}^{G}}{\mu_{t}}=F\left(\mu_{t}\right)-G\left(\mu_{t}\right)-\left(\mu_{t}\cdot \left(F\left(\mu_{t}\right)-G\left(\mu_{t}\right)\right)\right)1,$$

so that

(41) $$p_{T}^{\mu ,p}\left(\pi^{F}-\pi^{G}\right)≲{∥F\left(\mu \right)-G\left(\mu \right)∥}_{V_{\mu }^{p},\left[0,T\right]}+{∥\mu \cdot \left(F\left(\mu \right)-G\left(\mu \right)\right)∥}_{V_{\mu }^{p},\left[0,T\right]}.$$

Similarly to the proof of Lemma 4.3, noting that $R_{s,t}^{\mu \cdot (F(\mu )-G(\mu ))}=\mu_{s}\cdot R_{s,t}^{F(\mu )-G(\mu )}+\mu_{s,t}\cdot {\left(F\left(\mu \right)-G\left(\mu \right)\right)}_{s,t}$, we have that

$$\left|R_{s,t}^{\mu \cdot (F(\mu )-G(\mu ))}\right|\leq {∥\mu ∥}_{\infty ,[0,T]}\left|R_{s,t}^{F(\mu )-G(\mu )}\right|+{\left|\mu_{s,t}{||\left(F\left(\mu \right)-G\left(\mu \right)\right)}_{s,t}{|≲∥F-G∥}_{C^{2}}|\mu_{s,t}\right|}^{2},$$

where we used the fact that μ is bounded, and we deduce that

$${∥\mu \cdot \left(F\left(\mu \right)-G\left(\mu \right)\right)∥}_{V_{\mu }^{p},\left[0,T\right]}≲{∥F-G∥}_{C^{2}}\left(1+{∥\mu ∥}_{p,[0,T]}^{2}\right).$$

Combining this with Equations (40) and (41), we obtain the estimate in Equation (39), which then implies the desired continuity of Φ.□

Lemma 4.11 For any T>0 and any $F,G\in C^{2+\alpha ,K}\left({\bar{\Delta }}_{+}^{d};R^{d}\right)$, we have that

(42) $$|\log V_{T}^{\pi^{F}}-\log V_{T}^{\pi^{G}}{|\leq C∥F-G∥}_{C^{2}}{(1+∥\mu ∥}_{p,[0,T]}^{2})\xi_{T},$$

where $\xi_{T}$ is defined as in Equation (30), and the constant *C* depends only on p,d, and *K*.

We recall that during the proof of Lemma 4.7, we showed that

$$\left|\log V_{T}^{\pi^{F}}-\log V_{T}^{\pi^{G}}\right|\leq \left|\int_{0}^{T}\frac{\pi_{s}^{F}-\pi_{s}^{G}}{\mu_{s}}\;d\mu_{s}\right|+\frac{1}{2}\left|\sum_{i,j=1}^{d}\int_{0}^{T}\frac{\left(\pi_{s}^{F,i}-\pi_{s}^{G,i}\right)\left(\pi_{s}^{F,j}+\pi_{s}^{G,j}\right)}{\mu_{s}^{i}\mu_{s}^{j}}\;d{\left[\mu \right]}_{s}^{ij}\right|,$$

and (in the current setting replacing $q^{\prime }$ by *p*)

$$\left|\int_{0}^{T}\frac{\pi_{s}^{F}-\pi_{s}^{G}}{\mu_{s}}\;d\mu_{s}\right|≲p_{T}^{\mu ,p}\left(\left(\pi^{F},\pi^{F,\prime }\right)-\left(\pi^{G},\pi^{G,\prime }\right)\right)\left({∥\mu ∥}_{p,[0,T]}+{∥A^{\mu }∥}_{\frac{p}{2},\left[0,T\right]}\right).$$

By the estimate in Equation (39), we obtain

$$\left|\int_{0}^{T}\frac{\pi_{s}^{F}-\pi_{s}^{G}}{\mu_{s}}\;d\mu_{s}\right|≲\left(1+{∥\mu ∥}_{p,[0,T]}^{2}\right)\left({∥\mu ∥}_{p,[0,T]}+{∥A^{\mu }∥}_{\frac{p}{2},\left[0,T\right]}\right){∥F-G∥}_{C^{2}}.$$

Since ${∥F∥}_{C^{2+\alpha }}\leq K$ and ${∥G∥}_{C^{2+\alpha }}\leq K$, recalling Equation (28), we can verify that

$$\left|\frac{\left(\pi_{s}^{F,i}-\pi_{s}^{G,i}\right)\left(\pi_{s}^{F,j}+\pi_{s}^{G,j}\right)}{\mu_{s}^{i}\mu_{s}^{j}}\right|≲{∥F-G∥}_{C^{2}}.$$

Hence, we have that

$$\left|\sum_{i,j=1}^{d}\int_{0}^{T}\frac{\left(\pi_{s}^{F,i}-\pi_{s}^{G,i}\right)\left(\pi_{s}^{F,j}+\pi_{s}^{G,j}\right)}{\mu_{s}^{i}\mu_{s}^{j}}\;d{\left[\mu \right]}_{s}^{ij}\right|≲{∥F-G∥}_{C^{2}}\sum_{i=1}^{d}{\left[\mu \right]}_{T}^{ii}.$$

Combining the estimates above, we obtain Equation (42).□

As a special case of Theorem 4.9, we can deduce an asymptotic growth rate for the universal portfolio in the case that our portfolios are restricted to the class $F^{2+\alpha ,K}$ of functionally controlled portfolios.

Let *m* be a fixed probability measure on $C^{2+\alpha ,K}=C^{2+\alpha ,K}\left({\bar{\Delta }}_{+}^{d};R^{d}\right)$, and define $\nu :=\Phi_{*}m$ as the pushforward measure on $F^{2+\alpha ,K}$ of *m* under the map Φ given in Lemma 4.10. The universal portfolio based on functionally controlled portfolios is then defined by

(43) $$\pi_{t}^{\nu }:=\frac{\int_{F^{2+\alpha ,K}}\pi_{t}V_{t}^{\pi }\;d\nu \left(\pi \right)}{\int_{F^{2+\alpha ,K}}V_{t}^{\pi }\;d\nu \left(\pi \right)},\;t\in \left[0,\infty \right),$$

and the wealth process of the best retrospectively chosen portfolio is defined as

(44) $$V_{T}^{*,K,\alpha }:=\underset{\pi \in F^{2+\alpha ,K}}{\sup}V_{T}^{\pi }=\underset{F\in C^{2+\alpha ,K}}{\sup}V_{T}^{\pi^{F}}.$$

By Lemma 4.11, the mapping $F\mapsto V_{T}^{\pi^{F}}$ is a continuous map on $C^{2+\alpha ,K}$ with respect to the *C* ^2^‐norm. We also have that $C^{2+\alpha ,K}$ is compact with respect to the *C* ^2^‐norm (see Cuchiero et al. (2019, Lemma 4.1)). Combining these two facts, we see that, for each T>0, there exists a function $F_{T}^{*}\in C^{2+\alpha ,K}$ such that

$$V_{T}^{*,K,\alpha }=V_{T}^{\pi^{F_{T}^{*}}}.$$

Theorem 4.12 Let *m* be a probability measure on $C^{2+\alpha ,K}$ with full support. Let $\pi^{\nu }$ be the universal portfolio as defined in Equation (43), and define $V_{T}^{*,K,\alpha }$ as in Equation (44).

(i) If $\lim_{T\to \infty }{(1+∥\mu ∥}_{p,[0,T]}^{2})\xi_{T}=\infty$, where as usual $\xi_{T}$ is defined as in Equation (30), then
(45) $$\lim_{T\to \infty }\frac{1}{{(1+∥\mu ∥}_{p,[0,T]}^{2})\xi_{T}}\left(\log V_{T}^{*,K,\alpha }-\log V_{T}^{\pi^{\nu }}\right)=0.$$
(ii) With the shorthand notation $\xi_{k,k+1}:={∥\mu ∥}_{p,[k,k+1]}+{∥A^{\mu }∥}_{\frac{p}{2},\left[k,k+1\right]}+\sum_{i=1}^{d}{\left[\mu \right]}_{k,k+1}^{ii}$ for each k∈N, if $\lim_{T\to \infty }\sum_{k=0}^{\lceil T\rceil -1}{(1+∥\mu ∥}_{p,[k,k+1]}^{2})\xi_{k,k+1}=\infty$, then
(46) $$\lim_{T\to \infty }\frac{1}{\sum_{k=0}^{\lceil T\rceil -1}{(1+∥\mu ∥}_{p,[k,k+1]}^{2})\xi_{k,k+1}}\left(\log V_{T}^{*,K,\alpha }-\log V_{T}^{\pi^{\nu }}\right)=0.$$

The result of part (i) follows from Theorem 4.9 applied with $K=C^{2+\alpha ,K}$, $d_{K}\left(F,G\right)={∥F-G∥}_{C^{2}}$, ι=Φ, and $\lambda \left(T\right)={(1+∥\mu ∥}_{p,[0,T]}^{2})\xi_{T}$, noting from the result of Lemma 4.11 that the bound in Equation (36) is indeed satisfied in this case.

The result of part (ii) follows similarly with $\lambda \left(T\right)=\sum_{k=0}^{\lceil T\rceil -1}{(1+∥\mu ∥}_{p,[k,k+1]}^{2})\xi_{k,k+1}$. That the bound in Equation (36) is satisfied in this case follows from a very straightforward adaptation of the proofs of Lemmas 4.10 and 4.11, whereby the same estimates are applied over the subinterval [k,k+1] for each k=0,…,⌈T⌉−1, and the integrals over [0, *T*] in the proof of Lemma 4.11 are trivially bounded by the sum of integrals over these subintervals.□

Remark 4.13 The result of Theorem 4.12 is stated for two different “clocks,” namely ${(1+∥\mu ∥}_{p,[0,T]}^{2})\xi_{T}$ and $\sum_{k=0}^{\lceil T\rceil -1}{(1+∥\mu ∥}_{p,[k,k+1]}^{2})\xi_{k,k+1}$. One may wonder whether one of these clocks always dominates the other, making one of the statements superfluous. However, this is not the case.

On the one hand, in Section 4.4 below, we will exhibit a particular scenario, which demonstrates the nontriviality of the growth rate established in Equation (45). In this setting, one may check that ${(1+∥\mu ∥}_{p,[0,T]}^{2})\xi_{T}$ gives a strictly better asymptotic rate than if one were to use the sum over a partition of subintervals, as in part (ii) of Theorem 4.12.

On the other hand, in Section 5 below, we will consider a probabilistic model, where the market portfolio μ is given by the solution of a stochastic differential equation driven by Brownian motion. Using the fact that Brownian motion has independent increments, and the strong law of large numbers, in Theorem 5.4, we will use Equation (46) to improve the asymptotic growth rate to *T*. That is, we will actually show that, almost surely,

$$\lim_{T\to \infty }\frac{1}{T}\left(\log V_{T}^{*,K,\alpha }-\log V_{T}^{\pi^{\nu }}\right)=0.$$

It is, therefore, valuable to include both parts of Theorem 4.12.

Remark 4.14 Strictly speaking, Theorems 4.9 (which also recovers the version of Cover's theorem established in Cuchiero et al. (2019)) and 4.12 do not say that the universal portfolio $\pi^{\nu }$ performs asymptotically as well as the best retrospectively chosen one; rather, they provide bounds on how large the gap can become as time increases. For instance, for classical functionally generated portfolios of the form in Equation (21), the gap is $o(\max_{i=1,\text{…},d}{\left[\mu \right]}_{T}^{ii})$, and for functionally controlled portfolios of the form in Equation (28), the gap is, for example, $o((1+{∥\mu ∥}_{p,[0,T]}^{2})\xi_{T})$.

### 4.4 The nontriviality of the asymptotic growth rate

In this section, we will show that the asymptotic growth rate $\lambda \left(T\right)={(1+∥\mu ∥}_{p,[0,T]}^{2})\xi_{T}$ for functionally controlled portfolios, as established in part (i) of Theorem 4.12, is nontrivial, in the sense that there exists an instance of the market portfolio $\mu ={\left(\mu_{t}\right)}_{t\in [0,\infty )}$ such that

$$\underset{T\to \infty }{lim sup}\frac{\log V_{T}^{*,K,\alpha }}{\left(1+{∥\mu ∥}_{p,[0,T]}^{2}\right)\xi_{T}}>0\;\text{and}\;\lim_{T\to \infty }\frac{\log V_{T}^{*,K,\alpha }-\log V_{T}^{\pi^{\nu }}}{\left(1+{∥\mu ∥}_{p,[0,T]}^{2}\right)\xi_{T}}=0,$$

where $\nu =\Phi_{*}m$ for an arbitrary probability measure *m* on $C^{2+\alpha ,K}$ with full support.

Lemma 4.15 Let p∈(2,3) as usual, and then fix λ>0 such that $\frac{1}{p}<\lambda <\frac{1}{2}$. Let d=3 and let $\mu ={\left(\mu_{t}\right)}_{t\in [0,\infty )}$ be the continuous $\Delta_{+}^{3}$‐valued path given by

$$\mu_{t}=\left(\begin{array}{c} \mu_{t}^{1}\\ \mu_{t}^{2}\\ \mu_{t}^{3} \end{array}\right)=\left(\begin{array}{c} \frac{1}{3}\left(1+\frac{k^{-\lambda }}{3}\left(1-\cos t\right)\right)\\ \frac{1}{3}\left(1+\frac{k^{-\lambda }}{3}\sin t\right)\\ \frac{1}{3}\left(1+\frac{k^{-\lambda }}{3}\left(\cos t-1-\sin t\right)\right) \end{array}\right),\;t\in \left[2\pi \left(k-1\right),2\pi k\right),$$

for each k∈N. For α∈(0,1] and K>0, let $V_{T}^{*,K,\alpha }$ be the wealth induced by the best retrospectively chosen portfolio over $F^{2+\alpha ,K}$ at time *T*. Then,

$$\underset{T\to \infty }{lim sup}\frac{\log V_{T}^{*,K,\alpha }}{\left(1+{∥\mu ∥}_{p,[0,T]}^{2}\right)\xi_{T}}>0.$$

Recall that for any portfolio π, it follows from Proposition 3.9 that

$$\log V_{T}^{\pi }=\int_{0}^{T}\frac{\pi_{s}}{\mu_{s}}\;d\mu_{s}-\frac{1}{2}\sum_{i,j=1}^{d}\int_{0}^{T}\frac{\pi_{s}^{i}\pi_{s}^{j}}{\mu_{s}^{i}\mu_{s}^{j}}\;d{\left[\mu \right]}_{s}^{ij}.$$

Clearly, since μ is continuous with bounded variation on every compact interval, we have that [μ]=0, so that the second term vanishes. For any functionally controlled portfolio $\pi^{F}\in F^{2+\alpha ,K}$, using the relation

$$\frac{\pi_{t}^{F,i}}{\mu_{t}^{i}}=F^{i}\left(\mu_{t}\right)+1-\sum_{j=1}^{d}\mu_{t}^{j}F^{j}\left(\mu_{t}\right),\;i=1,\text{…},d,$$

together with the fact that $\sum_{i=1}^{d}d\mu_{t}^{i}=0$ (since $\sum_{i=1}^{d}\mu_{t}^{i}=1$), we deduce that

(47) $$\log V_{T}^{\pi^{F}}=\int_{0}^{T}\frac{\pi_{t}^{F}}{\mu_{t}}\;d\mu_{t}=\sum_{i=1}^{d}\int_{0}^{T}\frac{\pi_{t}^{F,i}}{\mu_{t}^{i}}\;d\mu_{t}^{i}=\sum_{i=1}^{d}\int_{0}^{T}F^{i}\left(\mu_{t}\right)\;d\mu_{t}^{i}.$$

We now choose the function $F\in C^{2+\alpha ,K}$ given by

$$F\left(x\right)=\left(\begin{array}{c} x_{2}\\ 0\\ 0 \end{array}\right)$$

for $x={\left(x_{1},x_{2},x_{3}\right)}^{⊤}\in {\bar{\Delta }}_{+}^{3}$. Substituting this function into Equation (47), we have

$$\log V_{T}^{\pi^{F}}=\int_{0}^{T}\frac{\pi_{t}^{F}}{\mu_{t}}\;d\mu_{t}=\sum_{i=1}^{3}\int_{0}^{T}F^{i}\left(\mu_{t}\right)\;d\mu_{t}^{i}=\int_{0}^{T}\mu_{t}^{2}\;d\mu_{t}^{1}.$$

For n∈N, we compute

$$\begin{array}{cc} \int_{0}^{2\pi n}\mu_{t}^{2}\;d\mu_{t}^{1} & =\sum_{k=1}^{n}\int_{2\pi (k-1)}^{2\pi k}\mu_{t}^{2}\;d\mu_{t}^{1}=\sum_{k=1}^{n}\int_{0}^{2\pi }\frac{1}{3}\left(1+\frac{k^{-\lambda }}{3}\sin t\right)\cdot \frac{k^{-\lambda }}{9}\sin t\;dt\\ & =\sum_{k=1}^{n}\frac{k^{-2\lambda }}{81}\int_{0}^{2\pi }\sin^{2}t\;dt=\frac{\pi }{81}\sum_{k=1}^{n}k^{-2\lambda }, \end{array}$$

and note that

$${∥\mu ∥}_{p,[0,2\pi n]}≲{\left(\sum_{k=1}^{n}k^{-\lambda p}\right)}^{\frac{1}{p}}<{\left(\sum_{k=1}^{\infty }k^{-\lambda p}\right)}^{\frac{1}{p}}<\infty$$

for every n∈N.

Writing $A_{s,t}^{\mu }={\left[A_{s,t}^{\mu ,i,j}\right]}_{i,j=1,2,3}=\int_{s}^{t}\left(\mu_{u}-\mu_{s}\right)⊗d\mu_{u}$ for the canonical rough path lift of μ, and using the monotonicity of trigonometric functions on the intervals $\left[0,\frac{\pi }{2}\right]$, $\left[\frac{\pi }{2},\pi \right]$, $\left[\pi ,\frac{3\pi }{2}\right]$, and $\left[\frac{3\pi }{2},2\pi \right]$, one can readily check that

$$∥A^{\mu }{∥}_{\frac{p}{2},\left[0,2\pi n\right]}∼A_{0,2\pi n}^{\mu ,2,1}=\int_{0}^{2\pi n}\mu_{t}^{2}\;d\mu_{t}^{1}∼\sum_{k=1}^{n}k^{-2\lambda }.$$

Recalling that $\xi_{T}={∥\mu ∥}_{p,[0,T]}+{∥A^{\mu }∥}_{\frac{p}{2},\left[0,T\right]}$ (since [μ]=0), and combining the calculations above, we deduce that, for T=2πn,

$$\frac{\log V_{T}^{*,K,\alpha }}{\left(1+{∥\mu ∥}_{p,[0,T]}^{2}\right)\xi_{T}}\geq \frac{\log V_{2\pi n}^{\pi^{F}}}{\left(1+{∥\mu ∥}_{p,[0,2\pi n]}^{2}\right)\xi_{2\pi n}}≳\frac{\sum_{k=1}^{n}k^{-2\lambda }}{1+\sum_{k=1}^{n}k^{-2\lambda }}\;⟶\;1\;\text{as}\;n\to \infty ,$$

where we used the fact that 2λ<1.□

The example in Lemma 4.15 thus shows that for functionally controlled portfolios $\pi^{F}$ generated by a function $F\in C^{2+\alpha ,K}$ which is not necessarily of gradient‐type, the asymptotic growth rate ${(1+∥\mu ∥}_{p,[0,T]}^{2})\xi_{T}$ appearing in Theorem 4.12 is actually sharp, in the sense that the log‐relative wealth $\log V_{T}^{\pi^{F}}$ and the rate ${(1+∥\mu ∥}_{p,[0,T]}^{2})\xi_{T}$ grow at the same rate (up to a multiplicative constant) as T→∞.

### 4.5 Functionally controlled portfolios have better performance

Let us conclude this section by showing that classical functionally generated portfolios of form in Equation (21), which are induced by functions of gradient type, are in general not optimal among the class of functionally controlled portfolios of the form in Equation (28).

Let μ be a continuous $\Delta_{+}^{d}$‐valued path which, for simplicity, we assume to have finite variation on every bounded interval (and which, therefore, trivially satisfies Property (RIE)). For any $F\in C^{2+\alpha ,K}\left({\bar{\Delta }}_{+}^{d};R^{d}\right)$, we know, as we saw in Equation (47) above, that for every T>0,

$$\log V_{T}^{\pi^{F}}=\int_{0}^{T}\frac{\pi_{s}^{F}}{\mu_{s}}\;d\mu_{s}-\frac{1}{2}\sum_{i,j=1}^{d}\int_{0}^{T}\frac{\pi_{s}^{F,i}\pi_{s}^{F,j}}{\mu_{s}^{i}\mu_{s}^{j}}\;d{\left[\mu \right]}_{s}^{ij}=\int_{0}^{T}F\left(\mu_{s}\right)\;d\mu_{s},$$

since the quadratic variation [μ] vanishes. Suppose now that the generating function *F* were of gradient‐type, so that F=∇f for some suitably smooth real‐valued function *f*. We then have that

$$\log V_{T}^{\pi^{F}}=\int_{0}^{T}\nabla f\left(\mu_{s}\right)\;d\mu_{s}=f\left(\mu_{T}\right)-f\left(\mu_{0}\right),$$

which implies together with the mean value theorem that

$$\left|\log V_{T}^{\pi^{F}}\right|\leq {∥\nabla f∥}_{\infty }|\mu_{T}-\mu_{0}{|=∥F∥}_{\infty }\left|\mu_{T}-\mu_{0}\right|\leq 2K,$$

as ${∥F∥}_{\infty }\leq K$ and $\mu_{T},\mu_{0}\in \Delta_{+}^{d}$. In particular, we have that

(48) $$\underset{T\geq 0}{\sup}\;\log V_{T}^{\pi^{F}}\leq 2K<\infty$$

for every generating function *F* of gradient type.

Now let μ be the market portfolio given in Lemma 4.15, and let $F\left(x_{1},x_{2},x_{3}\right)={\left(x_{2},0,0\right)}^{⊤}$, which we note is *not* of gradient type. In the proof of Lemma 4.15 we saw, for T=2πn with any n∈N, that $\log V_{T}^{\pi^{F}}=\int_{0}^{T}\mu_{t}^{2}\;d\mu_{t}^{1}=\frac{\pi }{81}\sum_{k=1}^{n}k^{-2\lambda }$ for some positive $\lambda <\frac{1}{2}$. We thus immediately have that

(49) $$\underset{T\to \infty }{lim sup}\;\log V_{T}^{\pi^{F}}=\infty .$$

Comparing Equation (49) with Equation (48), it is clear that the best retrospectively chosen portfolio over the set of functionally controlled portfolios cannot be of gradient type. Indeed, we infer that among the class of all functionally controlled portfolios, those corresponding to gradient‐type generating functions are in general far from being optimal, demonstrating the need to go beyond gradient‐type generating functions.

## 5 FUNCTIONALLY CONTROLLED PORTFOLIOS IN PROBABILISTIC MODELS

In this section, we shall demonstrate some further links between our purely pathwise theory and classical SPT in a probabilistic setting. In particular, this will allow us to illustrate again the advantages of functionally controlled portfolios, as introduced in Example 4.4, compared to (pathwise) functionally generated portfolios (see Lemma 4.3), as were previously treated in Schied et al. (2018); Cuchiero et al. (2019) based on Föllmer integration.

### 5.1 Probabilistic model for the market portfolio

Whereas in the previous sections we worked in a purely pathwise setting, we now assume that the market portfolio (also known as the market weights process) $\mu ={\left(\mu_{t}^{1},\text{…},\mu_{t}^{d}\right)}_{t\in [0,\infty )}$ is described by a time‐homogeneous Markovian Itô‐diffusion with values in $\Delta_{+}^{d}$, of the form

(50) $$\mu_{t}=\mu_{0}+\int_{0}^{t}c\left(\mu_{s}\right)\lambda \left(\mu_{s}\right)\;ds+\int_{0}^{t}\sqrt{c(\mu_{s})}\;dW_{s},\;t\in \left[0,\infty \right),$$

where μ_0_ is distributed according to some measure ρ on $\Delta_{+}^{d}$, *W* is a *d*‐dimensional Brownian motion and $\sqrt{\;\cdot \;}$ denotes the matrix square root. We assume that μ is the canonical process defined on path space (Ω,F,P), that is, $\Omega =C(\left[0,\infty \right);\Delta_{+}^{d})$, $F=\sigma (\mu_{t}:t\in \left[0,\infty \right))$, and P denotes the law of μ. For the moment, λ is just assumed to be a Borel measurable function from $\Delta_{+}^{d}$ to $R^{d}$. Writing $S_{+}^{d}$ for the set of positive semi‐definite symmetric matrices, $c\in C({\bar{\Delta }}_{+}^{d};S_{+}^{d})$ is such that

$$c\left(x\right)1=0\;\text{for all}\;x\in \Delta_{+}^{d}.$$

The latter requirement is necessary to guarantee that the process μ lies in $\Delta_{+}^{d}$. For a complete characterization of stochastic invariance of the closed simplex (under additional regularity conditions on the coefficients λ and *c*), we refer to Abi Jaber et al. (2019, Theorem 2.3) and the references therein. To ensure that the process stays in the open simplex $\Delta_{+}^{d}$, conditions for nonattainment of the boundary are established for instance in Filipović and Larsson (2016, Theorem 5.7). These conditions build on versions of what is sometimes called “McKean's argument” (see Mayerhofer et al. (2011) for an overview and further references).

We further suppose that the so‐called *structure condition* is satisfied, that is

(51) $$\int_{0}^{T}\lambda^{⊤}\left(\mu_{s}\right)c\left(\mu_{s}\right)\lambda \left(\mu_{s}\right)\;ds<\infty \;P\text{-a.s.},\;\text{for all}\;T\in \left[0,\infty \right),$$

which is equivalent to “no unbounded profit with bounded risk” (NUPBR); see, for example, Theorem 3.4 in Hulley and Schweizer (2010).

Remark 5.1 As (NUPBR) is satisfied due to Equation (51), the sample paths of μ almost surely satisfy Property (RIE) with respect to every p∈(2,3) and a suitable sequence of partitions, compare Remark 2.8.

We further impose the following ergodicity assumption in the spirit of Eberle (2016, Section 2.2, Theorem 2.6 and Section 2.2.3, Theorem 2.8), along with an integrability condition on λ.

Assumption 5.2 We assume that the market portfolio μ, given by the dynamics in Equation (50), is an ergodic process with stationary measure ρ on $\Delta_{+}^{d}$. That is, we suppose that $\rho p_{t}=\rho$ for every t∈[0,∞), where here ${\left(p_{t}\right)}_{t\in [0,\infty )}$ denotes the transition probability of μ. Furthermore, we suppose that $\lambda \in L^{2}\left(\Delta_{+}^{d},\rho ;R^{d}\right)$.

Note that the assumption that ρ is a stationary measure implies that the shift semigroup $\Theta_{t}\left(\omega \right)=\omega \left(t+\cdot \right)$, t∈[0,∞), ω∈Ω, preserves the measure P, in the sense that $P\circ \Theta_{t}^{-1}=P$. Hence, the “ergodic theorem in continuous time” (see Eberle (2016, Section 2.2, Theorem 2.6, Theorem 2.8)) can be applied.

While on the pathwise market $\Omega_{p}$, the portfolios were given by μ‐controlled paths $\left(\pi ,\pi^{\prime }\right)\in V_{\mu }^{q}$ (recall Definition 2.3), in the present semimartingale setting, we consider a portfolio π to be an element of the set Π of all predictable processes π taking values in $\Delta^{d}$, such that the Itô integral

$$\int_{0}^{T}\frac{\pi_{s}}{\mu_{s}}\;d\mu_{s}=\int_{0}^{T}\sum_{i=1}^{d}\frac{\pi_{s}^{i}}{\mu_{s}^{i}}\;d\mu_{s}^{i}$$

is well‐defined for every T∈[0,∞). As established in Cuchiero et al. (2019, Section 4.2.3), for π∈Π, the relative wealth process (recall Equation 14) can be written in the usual form, that is

(52) $$\begin{array}{cc} V_{T}^{\pi } & =\exp\left(\int_{0}^{T}\frac{\pi_{s}}{\mu_{s}}\;d\mu_{s}-\frac{1}{2}\int_{0}^{T}\sum_{i,j=1}^{d}\frac{\pi_{s}^{i}\pi_{s}^{j}}{\mu_{s}^{i}\mu_{s}^{j}}c^{ij}\left(\mu_{s}\right)\;ds\right),\;T\in \left[0,\infty \right). \end{array}$$

Remark 5.3 Note that if $\left(\pi ,\pi^{\prime }\right)$ is an adapted process with sample paths, which are almost surely μ‐controlled paths, then it is predictable, and under Property (RIE), the rough integral interpretation of $\int_{0}^{T}\frac{\pi_{s}}{\mu_{s}}\;d\mu_{s}$ coincides almost surely with the Itô integral interpretation. Indeed, the rough integral can be approximated by left‐point Riemann sums (see Theorem 2.12), while the Itô integral can be approximated by the same Riemann sums in probability (see, e.g., Protter (2004, Theorem II.21)). Moreover, as established in Proposition 3.9, the identity in Equation (52) holds even in a pathwise setting.

### 5.2 The log‐optimal portfolio and equivalence of its asymptotic growth rate with Cover's universal and the best retrospectively chosen portfolio

The results in this section will illustrate that in the presence of an appropriate probabilistic structure, the asymptotic growth rate can be significantly improved for scenarios outside a null set.

For a given T>0, the *log‐optimal portfolio* $\hat{\pi }$ is the maximizer of the optimization problem

(53) $$\underset{\pi \in \Pi }{\sup}E\left[\log V_{T}^{\pi }\right].$$

We write

$${\hat{V}}_{T}:=V_{T}^{\hat{\pi }}$$

for the corresponding wealth process. As shown in Cuchiero et al. (2019, Section 4.2.3), if μ satisfies the dynamics in Equation (50), then $\hat{\pi }=\left({\hat{\pi }}^{1},\cdots ,{\hat{\pi }}^{d}\right)$ can be expressed as

(54) $$\begin{array}{c} {\hat{\pi }}_{t}^{i}=\mu_{t}^{i}\left(\lambda^{i}\left(\mu_{t}\right)+1-\sum_{j=1}^{d}\mu_{t}^{j}\lambda^{j}\left(\mu_{t}\right)\right),\;t\in \left[0,\infty \right), \end{array}$$

and, due to Equation (52), the expected value of the log‐optimal portfolio satisfies

(55) $$\begin{array}{c} E\left[\log{\hat{V}}_{T}\right]=\underset{\pi \in \Pi }{\sup}E\left[\log V_{T}^{\pi }\right]=\frac{1}{2}E\left[\int_{0}^{T}\lambda^{⊤}\left(\mu_{s}\right)c\left(\mu_{s}\right)\lambda \left(\mu_{s}\right)\;ds\right]. \end{array}$$

We suppose that the log‐optimal portfolio has finite maximal expected utility and require thus additionally to Equation (51) that

$$E\left[\int_{0}^{T}\lambda^{⊤}\left(\mu_{s}\right)c\left(\mu_{s}\right)\lambda \left(\mu_{s}\right)\;ds\right]<\infty .$$

From the expression in Equation (54), we see immediately that the log‐optimal portfolio $\hat{\pi }$ belongs to the class of functionally controlled portfolios, as defined in Example 4.4, whenever λ is sufficiently smooth. In general, however, it does *not* belong to the smaller class of functionally generated portfolios, as we will see in Section 5.3.

In Equation (53), the supremum is taken over *all* predictable strategies in Π. However, since the optimizer is actually of the form in Equation (54), we can also take the supremum in Equation (53) over a smaller set. Indeed, it is sufficient to consider (functionally controlled) portfolios of the form

(56) $${\left(\pi_{t}^{F}\right)}^{i}=\mu_{t}^{i}\left(F^{i}\left(\mu_{t}\right)+1-\sum_{j=1}^{d}\mu_{t}^{j}F^{j}\left(\mu_{t}\right)\right),$$

for functions *F* in the space $L^{2}\left(\Delta_{+}^{d},\rho ;R^{d}\right)$.

Clearly, any portfolio $\pi^{F}$ of the form in Equation (56) can itself be considered as a function $\pi^{F}\in L^{2}\left(\Delta_{+}^{d},\rho ;R^{d}\right)$, which maps $x\mapsto \pi^{F}\left(x\right)$, where

(57) $${\left[\pi^{F}\left(x\right)\right]}^{i}=x^{i}\left(F^{i}\left(x\right)+1-\sum_{j=1}^{d}x^{j}F^{j}\left(x\right)\right),$$

with the corresponding portfolio then being given by $t\mapsto \pi^{F}\left(\mu_{t}\right)$.

In the current probabilistic setting, we establish the following equivalence of the asymptotic growth rates of the log‐optimal, best retrospectively chosen and the universal portfolio based on functionally controlled portfolios of the form in Equation (56), which can be viewed as a generalization of Cuchiero et al. (2019, Theorem 4.12) for nonfunctionally generated portfolios.

Theorem 5.4 Let μ be a market weights process with the dynamics in Equation (50).

(i) Suppose that μ and λ satisfy Assumption 5.2, and that $c\in C({\bar{\Delta }}_{+}^{d};S_{+}^{d})$. Let *m* be a probability measure on $L^{2}\left(\Delta_{+}^{d},\rho ;R^{d}\right)$ such that λ∈supp(m). Define the universal portfolio $\pi^{\nu }$ analogously to Equation (43) but with ν being the pushforward measure of *m* under the mapping $F\;\mapsto \;\pi^{F}$ with $\pi^{F}$ as in Equation (57), compare Cuchiero et al. (2019, Section 4.2.2). Suppose that there exists an integrable random variable *w* such that, for each T>0, the growth rate of the universal portfolio satisfies
(58) $$\frac{1}{T}\log V_{T}^{\pi^{\nu }}\geq -w.$$
  We then have that
(59) $$\underset{T\to \infty }{lim inf}\frac{1}{T}\log V_{T}^{\pi^{\nu }}=\lim_{T\to \infty }\frac{1}{T}\log{\hat{V}}_{T}=\hat{L},\;P\text{-a.s.},$$
  where $\hat{L}$ is given by
$$\hat{L}:=\frac{1}{2}\int_{\Delta_{+}^{d}}\lambda^{⊤}\left(x\right)c\left(x\right)\lambda \left(x\right)\;\rho \left(dx\right).$$
(ii) Suppose that
(60) $$\lambda \in C_{b}^{3}\left({\bar{\Delta }}_{+}^{d};R^{d}\right),\;\text{and}\;\sqrt{c}\in C_{b}^{3}\left({\bar{\Delta }}_{+}^{d};S_{+}^{d}\right).$$
  With the same notation as in Section 4.3, let *m* be a probability measure on $C^{2+\alpha ,K}$ with full support, and let $\nu =\Phi_{*}m$ be the pushforward measure on $F^{2+\alpha ,K}$ of *m* under the map Φ given in Lemma 4.10. Let $\pi^{\nu }$ be the universal portfolio as defined in Equation (43), and let $V^{*,K,\alpha }$ be the wealth process of the best retrospectively chosen portfolio, as in Equation (44). We then have that
(61) $$\lim_{T\to \infty }\frac{1}{T}\left(\log V_{T}^{*,K,\alpha }-\log V_{T}^{\pi^{\nu }}\right)=0,\;P\text{-a.s.}$$
(iii) Suppose that μ,λ, and *c* satisfy both Assumption 5.2 and Equation (60), and that K>0 is sufficiently large to ensure that $\lambda \in C^{2+\alpha ,K}$. Let $m,\nu ,\pi^{\nu }$, and $V^{*,K,\alpha }$ be as in part (ii) above. Then,
(62) $$\underset{T\to \infty }{lim inf}\frac{1}{T}\log V_{T}^{\pi^{\nu }}=\underset{T\to \infty }{lim inf}\frac{1}{T}\log V_{T}^{*,K,\alpha }=\lim_{T\to \infty }\frac{1}{T}\log{\hat{V}}_{T}=\hat{L},\;P\text{-a.s.}$$

Remark 5.5 Note that the assumption of ergodicity in Assumption 5.2 is only needed for assertions (i) and (iii). The equivalence of the asymptotic growth rates of the best retrospectively chosen and Cover's universal portfolio, as established in part (ii), holds for all Brownian‐driven SDEs with sufficiently smooth coefficients.

As preparation for the proof of Theorem 5.4, we need the following technical lemma, which is an adaptation of Hubalek et al. (2002, Lemma 3.1).

Lemma 5.6 Let ${\left(f_{n}\right)}_{n\in N}$ be a sequence of non‐negative measurable functions on some topological space A, such that the map $a\mapsto {lim inf}_{n\to \infty }f_{n}\left(a\right)$ is continuous at some point $\hat{a}\in A$. Let ν be a probability measure on A with $\hat{a}\in \mathrm{supp}\left(\nu \right)$. Then,

$$\underset{n\to \infty }{lim inf}f_{n}\left(\hat{a}\right)\leq \underset{n\to \infty }{lim inf}{\left(\int_{A}f_{n}^{n}\left(a\right)\;\nu \left(da\right)\right)}^{\frac{1}{n}}.$$

Let g≥0 be bounded measurable function such that $\int_{A}g\left(a\right)\;\nu \left(da\right)=1$. By Fatou's lemma and Hölder's inequality,

$$\begin{array}{cc} \int_{A} & \underset{n\to \infty }{lim inf}f_{n}\left(a\right)g\left(a\right)\;\nu \left(da\right)\leq \underset{n\to \infty }{lim inf}\int_{A}f_{n}\left(a\right)g\left(a\right)\;\nu \left(da\right)\\ & \leq \underset{n\to \infty }{lim inf}{\left(\int_{A}f_{n}^{n}\left(a\right)\;\nu \left(da\right)\right)}^{\frac{1}{n}}{\left(\int_{A}g^{\frac{n}{n-1}}\left(a\right)\;\nu \left(da\right)\right)}^{\frac{n-1}{n}}=\underset{n\to \infty }{lim inf}{\left(\int_{A}f_{n}^{n}\left(a\right)\;\nu \left(da\right)\right)}^{\frac{1}{n}}, \end{array}$$

where the last equality follows from the fact that $\lim_{n\to \infty }\int_{A}g^{\frac{n}{n-1}}\;\nu \left(da\right)=\int_{A}g\left(a\right)\;\nu \left(da\right)$ by the dominated convergence theorem. Since *g* was arbitrary, $\hat{a}$ lies in the support of ν, and ${lim inf}_{n\to \infty }f_{n}$ is continuous at $\hat{a}$, we deduce the result.□

Proof of Theorem 5.4 *Part (i)*: By the conditions on λ and *c*, and the fact that we consider portfolios of the form in Equation (56) with $F\in L^{2}\left(\Delta_{+}^{d},\rho ;R^{d}\right)$, we see that the assumptions of Cuchiero et al. (2019, Theorem 4.9) are satisfied. Thus, for each $F\in L^{2}\left(\Delta_{+}^{d},\rho ;R^{d}\right)$, we have that

(63) $$\lim_{T\to \infty }\frac{1}{T}\log V_{T}^{\pi^{F}}=L^{\pi^{F}},\;P\text{-a.s.},$$

where

$$L^{\pi^{F}}:=\int_{\Delta_{+}^{d}}{\left(\frac{\pi^{F}\left(x\right)}{x}\right)}^{⊤}c\left(x\right)\lambda \left(x\right)\;\rho \left(dx\right)-\frac{1}{2}\int_{\Delta_{+}^{d}}{\left(\frac{\pi^{F}\left(x\right)}{x}\right)}^{⊤}c\left(x\right)\left(\frac{\pi^{F}\left(x\right)}{x}\right)\;\rho \left(dx\right).$$

Taking the supremum over $F\in L^{2}\left(\Delta_{+}^{d},\rho ;R^{d}\right)$, we find that

$$\underset{F\in L^{2}\left(\Delta_{+}^{d},\rho ;R^{d}\right)}{\sup}L^{\pi^{F}}=L^{\pi^{\lambda }}=\hat{L}.$$

Recalling Equations (54) and (63), it follows that, P‐a.s.,

(64) $$\lim_{T\to \infty }\frac{1}{T}\log{\hat{V}}_{T}=\lim_{T\to \infty }\frac{1}{T}\log V_{T}^{\pi^{\lambda }}=L^{\pi^{\lambda }}=\hat{L}.$$

Note that the map

$$F\;\mapsto \;\exp\left(L^{\pi^{F}}\right)=\lim_{T\to \infty }{\left(V_{T}^{\pi^{F}}\right)}^{\frac{1}{T}}$$

is continuous with respect to the $L^{2}\left(\Delta_{+}^{d},\rho ;R^{d}\right)$‐norm. Thus, applying Lemma 5.6 with $f_{T}\left(F\right)={\left(V_{T}^{\pi^{F}}\right)}^{\frac{1}{T}}$, and recalling Lemma 4.8, we deduce that

(65) $$\lim_{T\to \infty }\frac{1}{T}\log V_{T}^{\pi^{\lambda }}\leq \underset{T\to \infty }{lim inf}\frac{1}{T}\log V_{T}^{\pi^{\nu }},\;P\text{-a.s.}$$

On the other hand, by the definition of the log‐optimal portfolio,

(66) $$E\left[\log V_{T}^{\pi^{\nu }}\right]\leq E\left[\log{\hat{V}}_{T}\right].$$

By Equation (55) and the ergodicity of the process μ, we have that

(67) $$\lim_{T\to \infty }\frac{1}{T}E\left[\log{\hat{V}}_{T}\right]=\hat{L}.$$

By Fatou's lemma (which we may apply by the condition in Equation (58)), Equations (66), (67), (64), and (65), we then have that, P‐a.s.,

$$\begin{array}{cc} E\left[\underset{T\to \infty }{lim inf}\frac{1}{T}\log V_{T}^{\pi^{\nu }}\right] & \leq \underset{T\to \infty }{lim inf}\frac{1}{T}E\left[\log V_{T}^{\pi^{\nu }}\right]\leq \underset{T\to \infty }{lim inf}\frac{1}{T}E\left[\log{\hat{V}}_{T}\right]\\ & =\hat{L}=\lim_{T\to \infty }\frac{1}{T}\log{\hat{V}}_{T}\leq \underset{T\to \infty }{lim inf}\frac{1}{T}\log V_{T}^{\pi^{\nu }}, \end{array}$$

from which the result (59) follows.

*Part (ii)*: The process μ is assumed to satisfy the Itô SDE (50), but since the vector fields λ(·)c(·) and $\sqrt{c}\left(\cdot \right)$ are in *C* ^3^ with bounded derivatives, μ also coincides almost surely with the unique solution of the rough differential equation

$$\mu_{t}=\mu_{0}+\int_{0}^{t}c\left(\mu_{s}\right)\lambda \left(\mu_{s}\right)\;ds+\int_{0}^{t}\sqrt{c(\mu_{s})}\;dW_{s},$$

driven by the standard Itô‐rough path lift W=(W,W) of *W* (see, e.g., Friz and Hairer (2020)). By standard rough path estimates (see, e.g., Friz and Hairer (2020, (11.10))), for each k∈N, we may deduce an estimate of the form

$${∥\mu ∥}_{p,[k,k+1]}≲1+\left({∥W∥}_{p,[k,k+1]}\vee {∥W∥}_{p,[k,k+1]}^{p}\right),$$

where ${∥W∥}_{p,[k,k+1]}:={∥W∥}_{p,[k,k+1]}+{∥W∥}_{\frac{p}{2},\left[k,k+1\right]}^{\frac{1}{2}}$, and the implied multiplicative constant is independent of *k* and *T*. Using the bound in Equation (6), a similar estimate can be inferred for the rough path lift $A^{\mu }$ of μ, defined as in Equation (A.4). Writing tr(·) for the trace operator, it also follows from Lemma B.1 and the boundedness of *c* that

$$\sum_{i=1}^{d}{\left[\mu \right]}_{k,k+1}^{ii}=\text{tr}\left(\int_{k}^{k+1}c\left(\mu_{t}\right)\;d{\left[W\right]}_{t}\right)=\int_{k}^{k+1}\text{tr}\left(c\left(\mu_{t}\right)\right)\;dt≲1,$$

where we used that ${\left[W\right]}_{t}=tI_{d}$ as shown, for example, in Friz and Hairer (2020, Example 5.9). We, therefore, deduce the existence of a polynomial *g* such that

(68) $$\left(1+{∥\mu ∥}_{p,[k,k+1]}^{2}\right)\xi_{k,k+1}\leq {g(∥W∥}_{p,[k,k+1]})$$

for every k∈N, with $\xi_{k,k+1}$ defined as in Theorem 4.12.

Since Brownian motion is a Lévy process, the random variables $g(∥W{∥}_{p,[k,k+1]})$, k∈N, are independent and identically distributed. Moreover, by the enhanced Burkholder–Davis–Gundy inequality^3^ (see Friz and Victoir (2010, Theorem 14.12)) applied to each of the monomials comprising *g*, we have that ${E[g(∥W∥}_{p,[0,1]})]<\infty$. Thus, by the strong law of large numbers, we have that, almost surely,

(69) $$\frac{1}{T}\sum_{k=0}^{\lceil T\rceil -1}{g(∥W∥}_{p,[k,k+1]}{)\;⟶\;E[g(∥W∥}_{p,[0,1]})]\;\text{as}\;T\;⟶\;\infty .$$

From Equations (68), (69), and the result of part (ii) of Theorem 4.12, we then deduce that, almost surely,

$$\begin{array}{cc} & \underset{T\to \infty }{lim sup}\frac{1}{T}\left(\log V_{T}^{*,K,\alpha }-\log V_{T}^{\pi^{\nu }}\right)\\ & \leq \underset{T\to \infty }{lim sup}\frac{\sum_{k=0}^{\lceil T\rceil -1}{g(∥W∥}_{p,[k,k+1]})}{T}\cdot \frac{\log V_{T}^{*,K,\alpha }-\log V_{T}^{\pi^{\nu }}}{\sum_{k=0}^{\lceil T\rceil -1}{(1+∥\mu ∥}_{p,[k,k+1]}^{2})\xi_{k,k+1}}=0, \end{array}$$

which immediately implies Equation (61).

*Part (iii)*: We have from part (ii) that Equation (61) holds. It is straightforward to check that the result of part (i) also holds when we restrict to portfolios generated by functions $F\in C^{2+\alpha ,K}$. Thus, it suffices to verify the technical condition in Equation (58), since then part (i) implies that Equation (59) holds, which, combined with Equation (61), gives Equation (62).

To this end, we first note that, similarly to the proof of part (ii) above, we may deduce that there exists a polynomial *g* such that, for any $F\in C^{2+\alpha ,K}$,

$$\left|\log V_{T}^{\pi^{F}}\right|\leq {∥F∥}_{C^{2}}\sum_{k=0}^{\lceil T\rceil -1}g\left({∥W∥}_{p,[k,k+1]}\right)$$

for all T>0. In particular, we have that

$$\log V_{T}^{\pi^{F}}\geq -K\sum_{k=0}^{\lceil T\rceil -1}{g(∥W∥}_{p,[k,k+1]}).$$

Since, by Lemma 4.8, $V_{T}^{\pi^{\nu }}=\int_{C^{2+\alpha ,K}}V_{T}^{\pi^{F}}\;dm\left(F\right)$, and using Jensen's inequality, we then have

$$\frac{1}{T}\log V_{T}^{\pi^{\nu }}\geq \frac{1}{T}\int_{C^{2+\alpha ,K}}\log V_{T}^{\pi^{F}}\;dm\left(F\right)\geq -\frac{K}{T}\sum_{k=0}^{\lceil T\rceil -1}g\left({∥W∥}_{p,[k,k+1]}\right),$$

and, again by the strong law of large numbers, Equation (69) holds almost surely. It is also straightforward to verify that

$${\left(\frac{1}{T}\sum_{k=0}^{\lceil T\rceil -1}{g(∥W∥}_{p,[k,k+1]})\right)}^{2}\leq \frac{\left\lceil T\right\rceil }{T^{2}}\sum_{k=0}^{\lceil T\rceil -1}g{\left({∥W∥}_{p,[k,k+1]}\right)}^{2},$$

so that, for all T>1,

$$E\left[{\left(\frac{1}{T}\sum_{k=0}^{\lceil T\rceil -1}g\left({∥W∥}_{p,[k,k+1]}\right)\right)}^{2}\right]\leq \frac{{\left\lceil T\right\rceil }^{2}}{T^{2}}E\left[g{\left(∥W{∥}_{p,[0,1]}\right)}^{2}\right]\leq 4E\left[g{\left(∥W{∥}_{p,[0,1]}\right)}^{2}\right]<\infty .$$

We deduce that the family $\frac{1}{T}\sum_{k=0}^{\lceil T\rceil -1}{g(∥W∥}_{p,[k,k+1]})$ for T>1 is bounded in $L^{2}\left(\Omega ,P\right)$, and therefore uniformly integrable. Thus, $\frac{1}{T}\sum_{k=0}^{\lceil T\rceil -1}{g(∥W∥}_{p,[k,k+1]}{)\to E[g(∥W∥}_{p,[0,1]})]$ as T→∞ both almost surely and in $L^{1}\left(\Omega ,P\right)$. It follows that

$$\frac{1}{T}\log V_{T}^{\pi^{\nu }}\geq -w_{T},$$

for some random variables $w_{T}$, T>0, which converge as T→∞ to an integrable random variable *w* almost surely and in $L^{1}\left(\Omega ,P\right)$. Although weaker than the condition in Equation (58), it is straightforward to verify that this condition suffices, as it is sufficient for the application of Fatou's lemma in the proof of part (i).□

### 5.3 Comparison of functionally controlled and functionally generated portfolios

Recall that, as we observed from the expression in Equation (54), the log‐optimal portfolio $\hat{\pi }$ belongs to the class of functionally controlled portfolios, provided that the drift characteristic λ—as introduced in the model (50)—is sufficiently smooth. In fact, the log‐optimal portfolio $\hat{\pi }$ is known to be even a (classical) functionally generated portfolio if λ can be written in the gradient form

$$\lambda \left(x\right)=\nabla \log G\left(x\right)=\frac{\nabla G(x)}{G(x)},\;x\in \Delta_{+}^{d},$$

for some differentiable function $G:\Delta_{+}^{d}\to R_{+}$; see Cuchiero et al. (2019, Proposition 4.7).

Considering again the stochastic model in Equation (50), we shall show in this section that the log‐optimal portfolio may genuinely *not* be a functionally generated portfolio, but still a functionally controlled one, in cases when λ is not of the above gradient type. We will then illustrate numerically that the difference between the true log‐optimal portfolio and an approximate “best” portfolio based on a class of gradient type trading strategies can be substantial. This demonstrates that such extensions beyond classical functionally generated portfolios are crucial.

Let us consider a so‐called volatility stabilized market model of the form in Equation (50), where, for some γ>0, the diffusion matrix is given by

$$c^{ij}\left(\mu \right):=\gamma \mu^{i}\left(\delta_{ij}-\mu^{j}\right),\;i,j=1,\text{…},d,$$

where $\delta_{ij}$ is the Kronecker delta, and the drift is given by

c(μ)λ(μ)=Bμ,

where $B\in R^{d\times d}$ is defined by $B^{ij}:=\frac{1+\alpha }{2}\left(1-\delta_{ij}d\right)$ for some α>γ−1. In the context of SPT, these models were first considered in Fernholz and Karatzas (2005). The condition α>γ−1 assures nonattainment of the boundary, as proved in Cuchiero (2019, Proposition 5.7), that is, the process μ takes values in $\Delta_{+}^{d}$.

We can solve this linear system for λ, and find as general solution

$$\lambda^{i}\left(\mu \right)=\frac{1+\alpha }{2\gamma \mu^{i}}+C,\;i=1,\text{…},d,$$

for an arbitrary C∈R. Note that this is well‐defined as μ always stays within the interior of the unit simplex $\Delta_{+}^{d}$ due to the condition α>γ−1. We now define the function $f^{\alpha }:R_{+}^{d}\to R$ by

(70) $$\begin{array}{c} f^{\alpha }\left(x\right):=\frac{1+\alpha }{2\gamma }\sum_{i=1}^{d}\log\left(x^{i}\right)+C\sum_{i=1}^{d}x^{i}. \end{array}$$

Then $\partial_{i}f^{\alpha }\left(x\right)=\left(1+\alpha \right)/\left(2\gamma x^{i}\right)+C$ for i=1,…,d, so that

$$\lambda \left(x\right)=\nabla f^{\alpha }\left(x\right)=\nabla \log G\left(x\right),\;x\in \Delta_{+}^{d},$$

where $G\left(x\right):=\exp(f^{\alpha }\left(x\right))$. Hence, in this volatility stabilized model, the log‐optimal portfolio $\hat{\pi }$ can be realized as a functionally generated portfolio. It follows from Equation (55) that

$$\underset{\pi \in \Pi }{\sup}E\left[\log V_{T}^{\pi }\right]=\frac{{\left(1+\alpha \right)}^{2}}{8\gamma }\left(E\left[\int_{0}^{T}\sum_{i=1}^{d}\frac{1}{\mu_{s}^{i}}\;ds\right]-d^{2}T\right).$$

A generalization of this model is a polynomial model with the same diffusion matrix (for some fixed γ), but a more general drift matrix *B* just satisfying $B^{jj}=-\sum_{i\neq j}B^{ij}$ and $B^{ij}\geq 0$ for i≠j (see Cuchiero (2019, Definition 4.9)). In this case, λ is in general no longer of gradient type. To see this, let d=3, and

(71) $$\begin{array}{c} B=\left(\begin{array}{ccc} -p & q & r\\ p & -q & 0\\ 0 & 0 & -r \end{array}\right) \end{array}$$

for p,q,r>0 such that 2min(p,q,r)−γ≥0, where the latter condition is imposed to guarantee nonattainment of the boundary (see Cuchiero (2019, Propostion 5.7)). We refer also to Cuchiero (2019, Theorem 5.1) for the relation to (NUPBR) and relative arbitrages.

The solution λ of c(x)λ(x)=Bx is now found to be

$$\begin{array}{cc} \lambda^{1}\left(x\right) & =\frac{1}{\gamma }\left(r-p+q\frac{x^{2}}{x^{1}}+r\frac{x^{3}}{x^{1}}\right)+C,\\ \lambda^{2}\left(x\right) & =\frac{1}{\gamma }\left(r-q+p\frac{x^{1}}{x^{2}}\right)+C,\\ \lambda^{3}\left(x\right) & =C, \end{array}$$

which cannot be realized as a gradient, for instance since $\frac{\partial \lambda^{3}}{\partial x^{1}}\neq \frac{\partial \lambda^{1}}{\partial x^{3}}$.

Let us now compare the log‐optimal portfolio

$${\left({\hat{\pi }}_{t}\right)}^{i}=\mu_{t}^{i}\left(\lambda^{i}\left(\mu_{t}\right)+1-\sum_{j=1}^{d}\mu_{t}^{j}\lambda^{j}\left(\mu_{t}\right)\right)$$

with the functionally generated portfolio

$$\left(\pi_{t}^{\alpha }\right)=\mu_{t}^{i}\left(\partial_{i}f^{\alpha }\left(\mu_{t}\right)+1-\sum_{j=1}^{d}\mu_{t}^{j}\partial_{j}f^{\alpha }\left(\mu_{t}\right)\right),$$

with $f^{\alpha }$ as defined in Equation (70). We seek the value of α, which optimizes

$$\underset{\alpha }{\sup}E\left[\log V_{T}^{\pi^{\alpha }}\right].$$

By Equations (50) and (52), we have that

$$\begin{array}{cc} E & \left[\log V_{T}^{\pi^{\alpha }}\right]=E\left[\int_{0}^{T}\nabla^{⊤}f^{\alpha }\left(\mu_{s}\right)B\mu_{s}\;ds-\frac{1}{2}\int_{0}^{T}\nabla^{⊤}f^{\alpha }\left(\mu_{s}\right)c\left(\mu_{s}\right)\nabla f^{\alpha }\left(\mu_{s}\right)\;ds\right]\\ & =\frac{1+\alpha }{2\gamma }E\left[\int_{0}^{T}\left(\frac{1}{\mu_{s}^{1}},\text{…},\frac{1}{\mu_{s}^{d}}\right)B\mu_{s}\;ds\right]-\frac{{\left(1+\alpha \right)}^{2}}{8\gamma }\left(E\left[\int_{0}^{T}\sum_{i=1}^{d}\frac{1}{\mu_{s}^{i}}\;ds\right]-d^{2}T\right). \end{array}$$

Since this expression is concave in α, we find the optimizer $\alpha^{*}$ to be given by

$$\alpha^{*}=\frac{2E\left[\int_{0}^{T}\left(\frac{1}{\mu_{s}^{1}},\text{…},\frac{1}{\mu_{s}^{d}}\right)B\mu_{s}\;ds\right]}{E\left[\int_{0}^{T}\sum_{i=1}^{d}\frac{1}{\mu_{s}^{i}}\;ds\right]-d^{2}T}-1.$$

Note that if *B* is the drift matrix of a volatility stabilized market model with parameter α, the right‐hand side yields exactly α, and we find the correct log‐optimal portfolio. However, when we take $\pi^{\alpha^{*}}$ as an approximate portfolio, for instance in the case of *B* being of the form (71), this leads to Figure 1. There, with the parameters p=0.15, q=0.3, r=0.2, the functions $t\mapsto E[\log{\hat{V}}_{t}]$ (blue) and $t\mapsto E[\log V_{t}^{\pi^{\alpha^{*}}}]$ (orange) are plotted, where the expected value is computed via a Monte Carlo simulation. This shows a significantly better performance of the log‐optimal portfolio and, thus, illustrates a clear benefit from going beyond functionally generated portfolios in SPT.

## Data Availability

The simulated data that support the findings of this paper are available from the corresponding author upon reasonable request.

## ENDNOTES

1 Here and throughout, we adopt the convention that $\frac{0}{0}:=0$.

2 Note that Φ plays the role of the embedding ι in the previous section.

3 Strictly speaking, the enhanced BDG inequality was proved for geometric rough paths constructed via Stratonovich integration. However, since ${\left[W\right]}_{t}=tI_{d}$, it is easy to see that it also holds for the Itô lift W.

## APPENDIX A ON THE ROUGH PATH FOUNDATION

In this appendix, we collect some results regarding rough integration, including its associativity and a Fubini type theorem. While such elementary results are well‐known for stochastic Itô integration and other classical theories of integration, the presented results seem to be novel in the context of rough path theory and are essential for the model‐free portfolio theory developed in the previous sections.

Throughout this section, we will consider a general *p*‐rough path X=(X,X)—that is, we will *not* impose Property (RIE)—and, as usual, we will assume that p,q, and *r* satisfy Assumption 3.2, so that in particular 1<p/2≤r<p≤q<∞.

### A.1 Products of controlled paths

As a first step towards the associativity of rough integration, we show that the product of two controlled paths is again a controlled path; see Friz and Hairer (2020, Corollary 7.4) for a similar result in a Hölder‐rough path setting.
Lemma A.1 Let $X\in C^{p\text{-var}}\left(\left[0,T\right];R^{d}\right)$. The product operator Π, given by

$$\begin{array}{ccc} V_{X}^{q}\left(\left[0,T\right];R^{d}\right)\times V_{X}^{q}\left(\left[0,T\right];R^{d}\right) & \to & V_{X}^{q}\left(\left[0,T\right];R^{d}\right),\\ \left(\left(F,F^{\prime }\right),\left(G,G^{\prime }\right)\right) & \mapsto & (FG,{\left(FG\right)}^{\prime }), \end{array}$$

where ${\left(FG\right)}^{i}:=F^{i}G^{i}$ and ${\left({\left(FG\right)}^{\prime }\right)}^{ij}:={\left(F^{\prime }\right)}^{ij}G^{i}+F^{i}{\left(G^{\prime }\right)}^{ij}$ for every 1≤i,j≤d, is a continuous bilinear map, and comes with the estimate

(A.1)
$$∥\left(F,F^{\prime }\right)\left(G,G^{\prime }\right){∥}_{V_{X}^{q}}\leq C{\left(1+∥X{∥}_{p}\right)}^{2}∥F,F^{\prime }{∥}_{V_{X}^{q}}{∥G,G^{\prime }∥}_{V_{X}^{q}},$$

where the constant *C* depends on p,q,r, and the dimension *d*. We call $\Pi (\left(F,F^{\prime }\right),\left(G,G^{\prime }\right))$ the product of $\left(F,F^{\prime }\right)$ and $\left(G,G^{\prime }\right)$, which we sometimes denote simply by FG.

It is clear from its definition that Π is a bilinear map. Suppose $\left(F,F^{\prime }\right),\left(G,G^{\prime }\right)\in V_{X}^{q}$. For all 1≤i,j≤d and $\left(s,t\right)\in \Delta_{\left[0,T\right]}$, we have

(A.2)
$$\begin{array}{ccc} {∥\left(FG\right)}^{\prime }{∥}_{q} & ≲ & ∥F^{\prime }{∥}_{q}{∥G∥}_{\infty }+∥F^{\prime }{∥}_{\infty }{∥G∥}_{q}+{∥F∥}_{q}∥G^{\prime }{∥}_{\infty }+{∥F∥}_{\infty }{∥G^{\prime }∥}_{q}\\ & ≲ & {(∥F∥}_{\infty }+{∥F∥}_{q}+∥F^{\prime }{∥}_{\infty }+∥F^{\prime }{∥}_{q}{)(∥G∥}_{\infty }+{∥G∥}_{q}+∥G^{\prime }{∥}_{\infty }+∥G^{\prime }{∥}_{q})\\ & ≲ & {\left(1+∥X{∥}_{p}\right)}^{2}∥F,F^{\prime }{∥}_{V_{X}^{q}}{∥G,G^{\prime }∥}_{V_{X}^{q}}. \end{array}$$

To identify the remainder $R^{FG}$, we compute

$$\begin{array}{cc} {\left(FG\right)}_{s,t}^{i} & =F_{s,t}^{i}G_{s}^{i}+F_{s}^{i}G_{s,t}^{i}+F_{s,t}^{i}G_{s,t}^{i}\\ & =\left(\sum_{j=1}^{d}{\left(F^{\prime }\right)}_{s}^{ij}X_{s,t}^{j}+{\left(R^{F}\right)}_{s,t}^{i}\right)G_{s}^{i}+F_{s}^{i}\left(\sum_{j=1}^{d}{\left(G^{\prime }\right)}_{s}^{ij}X_{s,t}^{j}+{\left(R^{G}\right)}_{s,t}^{i}\right)+F_{s,t}^{i}G_{s,t}^{i}\\ & =\sum_{j=1}^{d}\left({\left(F^{\prime }\right)}_{s}^{ij}G_{s}^{i}+F_{s}^{i}{\left(G^{\prime }\right)}_{s}^{ij}\right)X_{s,t}^{j}+{\left(R^{F}\right)}_{s,t}^{i}G_{s}^{i}+F_{s}^{i}{\left(R^{G}\right)}_{s,t}^{i}+F_{s,t}^{i}G_{s,t}^{i}\\ & =\sum_{j=1}^{d}{\left({\left(FG\right)}^{\prime }\right)}_{s}^{ij}X_{s,t}^{j}+{\left(R^{FG}\right)}_{s,t}^{i}, \end{array}$$

where ${\left(R^{FG}\right)}_{s,t}^{i}:={\left(R^{F}\right)}_{s,t}^{i}G_{s}^{i}+F_{s}^{i}{\left(R^{G}\right)}_{s,t}^{i}+F_{s,t}^{i}G_{s,t}^{i}$. Using the fact that 2r≥p, we then estimate

(A.3)
$$\begin{array}{ccc} ∥R^{FG}{∥}_{r} & ≲ & ∥R^{F}{∥}_{r}{∥G∥}_{\infty }+{∥F∥}_{\infty }∥R^{G}{∥}_{r}+{∥F∥}_{2r}{∥G∥}_{2r}\\ & ≲ & {(1+∥X∥}_{p})(∥R^{F}{∥}_{r}∥G,G^{\prime }{∥}_{V_{X}^{q}}+∥F,F^{\prime }{∥}_{V_{X}^{q}}∥R^{G}{∥}_{r})+{∥F∥}_{p}{∥G∥}_{p}\\ & ≲ & {\left(1+∥X{∥}_{p}\right)}^{2}∥F,F^{\prime }{∥}_{V_{X}^{q}}{∥G,G^{\prime }∥}_{V_{X}^{q}}. \end{array}$$

The estimate (A.1) then follows from Equations (A.2) and (A.3).□

### A.2 Associativity of rough integration

The following proposition provides an associativity result for rough integration.
Proposition A.2 Let X=(X,X) be a *p*‐rough path and let $\left(Y,Y^{\prime }\right),\left(F,F^{\prime }\right),\left(G,G^{\prime }\right)\in V_{X}^{q}$ be controlled paths. Then, the pair $\left(Z,Z^{\prime }\right):=\left(\int_{0}^{\cdot }F_{u}\;dG_{u},FG^{\prime }\right)\in V_{X}^{q}$, and we have that

$$\int_{0}^{\cdot }Y_{u}\;dZ_{u}=\int_{0}^{\cdot }Y_{u}F_{u}\;dG_{u},$$

where on the left‐hand side, we have the integral of $\left(Y,Y^{\prime }\right)$ against $\left(Z,Z^{\prime }\right)$, and on the right‐hand side, we have the integral of $\left(YF,{\left(YF\right)}^{\prime }\right)$ against $\left(G,G^{\prime }\right)$, each defined in the sense of Lemma 2.6.

The fact that $\left(Z,Z^{\prime }\right)\in V_{X}^{p}$ follows from the estimate in Equation (6) combined with the relation $G_{s,t}=G_{s}^{\prime }X_{s,t}+R_{s,t}^{G}$. It also follows from Equation (6) that the function $H^{\int F\;dG}$, defined by

$$Z_{s,t}=\int_{s}^{t}F_{u}\;dG_{u}=F_{s}G_{s,t}+F_{s}^{\prime }G_{s}^{\prime }X_{s,t}+H_{s,t}^{\int F\;dG}$$

for $\left(s,t\right)\in \Delta_{T}$, has finite $\hat{p}$‐variation for some $\hat{p}<1$, and we can thus conclude that $\lim_{|P|\to 0}\sum_{[s,t]\in P}\left|H_{s,t}^{\int F\;dG}\right|=0$. We similarly obtain

$$\begin{array}{cc} \int_{s}^{t}Y_{u}\;dZ_{u} & =Y_{s}Z_{s,t}+Y_{s}^{\prime }Z_{s}^{\prime }X_{s,t}+H_{s,t}^{\int Y\;dZ},\\ \int_{s}^{t}Y_{u}F_{u}\;dG_{u} & =Y_{s}F_{s}G_{s,t}+{\left(YF\right)}_{s}^{\prime }G_{s}^{\prime }X_{s,t}+H_{s,t}^{\int YF\;dG}, \end{array}$$

with

$$\lim_{|P|\to 0}\sum_{[s,t]\in P}|H_{s,t}^{\int Y\;dZ}|=\lim_{|P|\to 0}\sum_{[s,t]\in P}\left|H_{s,t}^{\int YF\;dG}\right|=0.$$

Noting that ${\left(YF\right)}^{\prime }=YF^{\prime }+Y^{\prime }F$, we then calculate

$$\begin{array}{cc} \int_{s}^{t}Y_{u}\;dZ_{u} & =Y_{s}Z_{s,t}+Y_{s}^{\prime }Z_{s}^{\prime }X_{s,t}+H_{s,t}^{\int Y\;dZ}\\ & =Y_{s}\left(F_{s}G_{s,t}+F_{s}^{\prime }G_{s}^{\prime }X_{s,t}+H_{s,t}^{\int F\;dG}\right)+Y_{s}^{\prime }F_{s}G_{s}^{\prime }X_{s,t}+H_{s,t}^{\int Y\;dZ}\\ & =Y_{s}F_{s}G_{s,t}+\left(Y_{s}F_{s}^{\prime }+Y_{s}^{\prime }F_{s}\right)G_{s}^{\prime }X_{s,t}+Y_{s}H_{s,t}^{\int F\;dG}+H_{s,t}^{\int Y\;dZ}\\ & =\int_{s}^{t}Y_{u}F_{u}\;dG_{u}-H_{s,t}^{\int YF\;dG}+Y_{s}H_{s,t}^{\int F\;dG}+H_{s,t}^{\int Y\;dZ}. \end{array}$$

Taking $\lim_{|P|\to 0}\sum_{[s,t]\in P}$ on both sides, we obtain $\int_{0}^{T}Y_{u}\;dZ_{u}=\int_{0}^{T}Y_{u}F_{u}\;dG_{u}$.□

Remark A.3 Denoting the integration operator by •, the result of Proposition A.2 may be expressed formally as Y•(F•G)=(YF)•G. We, therefore, refer to this result as the *associativity* of rough integration.

### A.3 The canonical rough path lift of a controlled path

Given a *p*‐rough path X=(X,X) and a controlled path $\left(Z,Z^{\prime }\right)\in V_{X}^{q}$, one can use Lemma 2.6 to enhance *Z* in a canonical way to a *p*‐rough path Z=(Z,Z), where Z is defined by

(A.4)
$$Z_{s,t}:=\int_{s}^{t}Z_{u}\;dZ_{u}-Z_{s}Z_{s,t},\;\text{for}\;\left(s,t\right)\in \Delta_{\left[0,T\right]},$$

with the integral defined as in Equation (5). Indeed, we observe the following.
Lemma A.4 Let X=(X,X) be a *p*‐rough path and $\left(Z,Z^{\prime }\right)\in V_{X}^{q}$ be a controlled path. Then, Z=(Z,Z), as defined in Equation (A.4), is a *p*‐rough path. Moreover, if $\left(Y,Y^{\prime }\right)\in V_{Z}^{q}$, then $\left(Y,Y^{\prime }Z^{\prime }\right)\in V_{X}^{q}$ and

$$\int_{0}^{T}Y_{u}\;dZ_{u}=\int_{0}^{T}Y_{u}\;dZ_{u},$$

where on the left‐hand side, we have the rough integral of $\left(Y,Y^{\prime }\right)$ against Z, and on the right‐hand side, we have the integral of $\left(Y,Y^{\prime }Z^{\prime }\right)$ against $\left(Z,Z^{\prime }\right)$ as defined in Equation (5).

That Z=(Z,Z) is a *p*‐rough path follows immediately from Lemma 2.6. That $\left(Y,Y^{\prime }Z^{\prime }\right)\in V_{X}^{q}$ can be shown in a straightforward manner using the definition of controlled paths. Arguing similarly as in the proof of Proposition A.2 and using the same notation, we calculate, for $\left(s,t\right)\in \Delta_{\left[0,T\right]}$,

$$\begin{array}{cc} \int_{s}^{t}Y_{u}\;dZ_{u} & =Y_{s}Z_{s,t}+Y_{s}^{\prime }Z_{s,t}+H_{s,t}^{\int Y\;dZ}\\ & =Y_{s}Z_{s,t}+Y_{s}^{\prime }\left(Z_{s}^{\prime }Z_{s}^{\prime }X_{s,t}+H_{s,t}^{\int Z\;dZ}\right)+H_{s,t}^{\int Y\;dZ}\\ & =\int_{s}^{t}Y_{u}\;dZ_{u}-H_{s,t}^{\int Y\;dZ}+Y_{s}^{\prime }H_{s,t}^{\int Z\;dZ}+H_{s,t}^{\int Y\;dZ}. \end{array}$$

Taking $\lim_{|P|\to 0}\sum_{[s,t]\in P}$ on both sides, we obtain $\int_{0}^{T}Y_{u}\;dZ_{u}=\int_{0}^{T}Y_{u}\;dZ_{u}$.□

### A.4 The exponential of a rough path

Based on the bracket of a rough path (recall Definition 2.9), one can introduce the rough exponential analogously to the stochastic exponential of Itô calculus.
Lemma A.5 For a one‐dimensional *p*‐rough path X=(X,X) (so that in particular *X* is real‐valued) such that $X_{0}=0$, we introduce the rough exponential by

$$V_{t}:=\exp\left(X_{t}-\frac{1}{2}{\left[X\right]}_{t}\right),\;t\in \left[0,T\right].$$

Then ,*V* is the unique controlled path in $V_{X}^{p}$ satisfying the linear rough differential equation

(A.5)
$$V_{t}=1+\int_{0}^{t}V_{u}\;dX_{u},\;t\in \left[0,T\right],$$

with Gubinelli derivative $V^{\prime }=V$.

Applying the Itô formula of Proposition 2.10 with $Y=X-\frac{1}{2}\left[X\right]$, $Y^{\prime }=1$, and f=exp, we observe that the Young integrals cancel, so that *V* does indeed satisfy Equation (A.5). The uniqueness of solutions to Equation (A.5) follows from the stability of rough integration, provided in this setting by Friz and Zhang (2018, Lemma 3.4).□

### A.5 A Fubini‐type theorem for rough integration

In this subsection, we provide a Fubini‐type theorem for Bochner and rough integrals. A result of this type is mentioned in a Hölder‐rough path setting in Friz and Hairer (2020, Exercise 4.10).
Theorem A.6 Let X=(X,X) be a *p*‐rough path, let A be a measurable subset of $V_{X}^{q}$, and let ν be a probability measure on A. If $\int_{A}{∥K,K^{\prime }∥}_{V_{X}^{q}}\;d\nu <\infty$, then

$$\int_{0}^{T}\int_{A}K_{u}\;d\nu \;dX_{u}=\int_{A}\int_{0}^{T}K_{u}\;dX_{u}\;d\nu .$$

Due to $\int_{A}{∥K,K^{\prime }∥}_{V_{X}^{q}}\;d\nu <\infty$, the controlled path $\int_{A}\left(K,K^{\prime }\right)\;d\nu \in V_{X}^{q}$ exists as a well‐defined Bochner integral. For s<t, we have

$$\begin{array}{c} \int_{A}\int_{s}^{t}K_{u}\;dX_{u}\;d\nu -\int_{A}K_{s}\;d\nu \;X_{s,t}-\int_{A}K_{s}^{\prime }\;d\nu \;X_{s,t}=\int_{A}\left(\int_{s}^{t}K_{u}\;dX_{u}-K_{s}X_{s,t}-K_{s}^{\prime }X_{s,t}\right)\;d\nu \end{array}$$

and, by the estimate in Equation (4),

(A.6)
$$|\int_{s}^{t}K_{u}\;dX_{u}-K_{s}X_{s,t}-K_{s}^{\prime }X_{s,t}|\leq C(∥R^{K}{∥}_{r,[s,t]}{∥X∥}_{p,[s,t]}+∥K^{\prime }{∥}_{q,[s,t]}{∥X∥}_{\frac{p}{2},\left[s,t\right]}).$$

Since 1/r+1/p>1, there exists a $\hat{p}>p$ such that $1/r+1/\hat{p}=1$. By Hölder's inequality, for any partition P of [0, *T*], we have

$$\begin{array}{cc} \int_{A}\sum_{[s,t]\in P}∥R^{K}{∥}_{r,[s,t]}{∥X∥}_{p,[s,t]}\;d\nu & \leq \int_{A}{\left(\sum_{[s,t]\in P}{∥R^{K}∥}_{r,[s,t]}^{r}\right)}^{\frac{1}{r}}{\left(\sum_{[s,t]\in P}{∥X∥}_{p,[s,t]}^{\hat{p}}\right)}^{\frac{1}{\hat{p}}}\;d\nu \\ & \leq \int_{A}∥R^{K}{∥}_{r,[0,T]}\;d\nu \;{∥X∥}_{p,[0,T]}^{\frac{p}{\hat{p}}}\left(\max_{[s,t]\in P}{∥X∥}_{p,[s,t]}^{\frac{\hat{p}-p}{\hat{p}}}\right). \end{array}$$

Since $\int_{A}∥R^{K}{∥}_{r,[0,T]}\;d\nu \leq \int_{A}{∥K,K^{\prime }∥}_{V_{X}^{q}}\;d\nu <\infty$, and since $\left(s,t\right)\mapsto {∥X∥}_{p,[s,t]}$ is uniformly continuous, we deduce, treating the second term on the right‐hand side of Equation (A.6) similarly, that

$$\lim_{|P|\to 0}\sum_{[s,t]\in P}\int_{A}\left(\int_{s}^{t}K_{u}\;dX_{u}-K_{s}X_{s,t}-K_{s}^{\prime }X_{s,t}\right)\;d\nu =0.$$

Thus, we obtain

$$\begin{array}{cc} \int_{A}\int_{0}^{T}K_{u}\;dX_{u}\;d\nu & =\lim_{|P|\to 0}\sum_{[s,t]\in P}\int_{A}\int_{s}^{t}K_{u}\;dX_{u}\;d\nu \\ & =\lim_{|P|\to 0}\sum_{[s,t]\in P}\int_{A}K_{s}\;d\nu \;X_{s,t}+\int_{A}K_{s}^{\prime }\;d\nu \;X_{s,t}=\int_{0}^{T}\int_{A}K_{u}\;d\nu \;dX_{u}. \end{array}$$

□

## APPENDIX B ROUGH PATH THEORY ASSUMING PROPERTY (RIE)

In this section, we provide additional results concerning rough path theory assuming Property (RIE), and, in particular, we give a proof of Theorem 2.12. As usual, we adopt Assumption 3.2.

### B.1 On the bracket of a rough path

We begin with some properties of the bracket of a rough path, introduced in Definition 2.9.
Lemma B.1 Let X=(X,X) be a *p*‐rough path and let $\left(K,K^{\prime }\right)\in V_{X}^{q}$. Recall from Proposition A.2 that $\left(Z,Z^{\prime }\right):=\left(\int_{0}^{\cdot }K_{u}\;dX_{u},K\right)\in V_{X}^{q}$. Let Z=(Z,Z) be the canonical rough path lift of *Z*, as defined in Equation (A.4), so that in particular, the bracket [Z] of Z exists. Then,

$$\left[Z\right]=\int_{0}^{\cdot }\left(K_{u}⊗K_{u}\right)\;d{\left[X\right]}_{u},$$

where the right‐hand side is defined as a Young integral.

Since [X] has finite p/2‐variation, the integral

$$\int_{0}^{T}\left(K_{u}⊗K_{u}\right)\;d{\left[X\right]}_{u}=\lim_{|P|\to 0}\sum_{[s,t]\in P}\left(K_{s}⊗K_{s}\right){\left[X\right]}_{s,t}$$

exists as a Young integral. In the following, we shall abuse notation slightly by writing $H_{s,t}=o(|t-s|)$ whenever a function *H* satisfies $\lim_{|P|\to 0}\sum_{[s,t]\in P}\left|H_{s,t}\right|=0$. We have

$$\begin{array}{cc} {\left[Z\right]}_{s,t} & =Z_{s,t}⊗Z_{s,t}-2\text{Sym}\left(Z_{s,t}\right)\\ & =\left(K_{s}X_{s,t}+K_{s}^{\prime }X_{s,t}\right)⊗\left(K_{s}X_{s,t}+K_{s}^{\prime }X_{s,t}\right)-2\left(Z_{s}^{\prime }⊗Z_{s}^{\prime }\right)\text{Sym}\left(X_{s,t}\right)+o(|t-s|)\\ & =\left(K_{s}X_{s,t}\right)⊗\left(K_{s}X_{s,t}\right)-2\left(K_{s}⊗K_{s}\right)\text{Sym}\left(X_{s,t}\right)+o(|t-s|)\\ & =\left(K_{s}⊗K_{s}\right){\left[X\right]}_{s,t}+o(|t-s|). \end{array}$$

Taking $\lim_{|P|\to 0}\sum_{[s,t]\in P}$ on both sides, we obtain ${\left[Z\right]}_{T}=\int_{0}^{T}\left(K_{u}⊗K_{u}\right)\;d{\left[X\right]}_{u}$.□

Proposition B.2 Suppose that $S\in C(\left[0,T\right];R^{d})$ satisfies (RIE) with respect to *p* and ${\left(P^{n}\right)}_{n\in N}$. Let S=(S,S) be the associated rough path as defined in Equation (8). Let $\left(K,K^{\prime }\right)\in V_{S}^{q}$ and $\left(Z,Z^{\prime }\right)=\left(\int_{0}^{\cdot }K_{u}\;dS_{u},K\right)\in V_{S}^{q}$. Let Z=(Z,Z) be the canonical rough path lift of *Z* as defined in Equation (A.4), so that in particular, the bracket [Z] of Z exists. Then the following hold:

(i) The bracket [Z] has finite total variation, and is given by
  $${\left[Z\right]}_{t}=\lim_{n\to \infty }\sum_{k=0}^{N_{n}-1}Z_{t_{k}^{n}\wedge t,t_{k+1}^{n}\wedge t}⊗Z_{t_{k}^{n}\wedge t,t_{k+1}^{n}\wedge t},\;t\in \left[0,T\right].$$
(ii) Let Γ be a continuous path of finite p/2‐variation. Then, the path Y:=Z+Γ admits a canonical rough path lift Y=(Y,Y), such that
  (B.1)
  $${\left[Y\right]}_{t}={\left[Z\right]}_{t}=\lim_{n\to \infty }\sum_{k=0}^{N_{n}-1}Y_{t_{k}^{n}\wedge t,t_{k+1}^{n}\wedge t}⊗Y_{t_{k}^{n}\wedge t,t_{k+1}^{n}\wedge t},\;t\in \left[0,T\right].$$

(i) Since, by Lemma 2.11, [S] has finite variation, it follows from Lemma B.1 that the same is true of [Z]. By the estimate in Equation (4), we know that $Z_{s,t}=K_{s}S_{s,t}+K_{s}^{\prime }S_{s,t}+H_{s,t}$ for some *H* satisfying $\lim_{|P|\to 0}\sum_{[s,t]\in P}\left|H_{s,t}\right|=0$. It follows that
  $$\begin{array}{cc} \lim_{n\to \infty }\sum_{k=0}^{N_{n}-1}Z_{t_{k}^{n}\wedge t,t_{k+1}^{n}\wedge t}⊗Z_{t_{k}^{n}\wedge t,t_{k+1}^{n}\wedge t} & =\lim_{n\to \infty }\sum_{k=0}^{N_{n}-1}\left(K_{t_{k}^{n}\wedge t}S_{t_{k}^{n}\wedge t,t_{k+1}^{n}\wedge t}\right)⊗\left(K_{t_{k}^{n}\wedge t}S_{t_{k}^{n}\wedge t,t_{k+1}^{n}\wedge t}\right)\\ & =\int_{0}^{t}\left(K_{u}⊗K_{u}\right)\;d{\left[S\right]}_{u}={\left[Z\right]}_{t}. \end{array}$$
(ii) Since Γ has finite p/2‐variation, the Young integrals $\int_{s}^{t}Z_{s,u}⊗d\Gamma_{u}$, $\int_{s}^{t}\Gamma_{s,u}⊗dZ_{u}$, and $\int_{s}^{t}\Gamma_{s,u}⊗d\Gamma_{u}$ are well‐defined, and the function Y, defined by
  $$Y_{s,t}=Z_{s,t}+\int_{s}^{t}Z_{s,u}⊗d\Gamma_{u}+\int_{s}^{t}\Gamma_{s,u}⊗dZ_{u}+\int_{s}^{t}\Gamma_{s,u}⊗d\Gamma_{u},$$
  also has finite p/2‐variation. It follows that Y=(Y,Y) is a *p*‐rough path. The equality ${\left[Y\right]}_{t}={\left[Z\right]}_{t}$ follows easily from the integration by parts formula for Young integrals. The second equality in Equation (B.1) follows by a similar argument to the one in the proof of part (i).

□

### B.2 Proof—the rough integral as a limit of Riemann sums

Proof of Theorem 2.12 Let $\left(Y,Y^{\prime }\right)\in V_{S}^{q}$. Recalling the Itô formula for rough paths (Proposition 2.10), it follows from the associativity of Young and rough integrals (recall Proposition A.2) that

$$\int_{0}^{t}Y_{u}\;df{\left(S\right)}_{u}=\int_{0}^{t}Y_{u}Df\left(S_{u}\right)\;dS_{u}+\frac{1}{2}\int_{0}^{t}Y_{u}D^{2}f\left(S_{u}\right)\;d{\left[S\right]}_{u}.$$

By Perkowski and Prömel (2016, Theorem 4.19), we have

$$\int_{0}^{t}Y_{u}Df\left(S_{u}\right)\;dS_{u}=\lim_{n\to \infty }\sum_{k=0}^{N_{n}-1}Y_{t_{k}^{n}}Df\left(S_{t_{k}^{n}}\right)S_{t_{k}^{n}\wedge t,t_{k+1}^{n}\wedge t},$$

the convergence being uniform in t∈[0,T]. By Friz and Hairer (2020, Lemma 5.11), we have the pointwise convergence

(B.2)
$$\lim_{n\to \infty }\sum_{k=0}^{N_{n}-1}Y_{t_{k}^{n}}D^{2}f\left(S_{t_{k}^{n}}\right)S_{t_{k}^{n}\wedge t,t_{k+1}^{n}\wedge t}^{⊗2}=\int_{0}^{t}Y_{u}D^{2}f\left(S_{u}\right)\;d{\left[S\right]}_{u}.$$

Recalling Pólya's theorem (see, e.g., Rao (1962)), which asserts that pointwise convergence of distribution functions on R to a continuous limit implies the uniformity of this convergence, we see from the proof of Friz and Hairer (2020, Lemma 5.11) that the convergence in Equation (B.2) also holds uniformly for t∈[0,T]. Thus, we obtain

(B.3)
$$\int_{0}^{t}Y_{u}\;df{\left(S\right)}_{u}=\lim_{n\to \infty }\sum_{k=0}^{N_{n}-1}\left(Y_{t_{k}^{n}}Df\left(S_{t_{k}^{n}}\right)S_{t_{k}^{n}\wedge t,t_{k+1}^{n}\wedge t}+\frac{1}{2}Y_{t_{k}^{n}}D^{2}f\left(S_{t_{k}^{n}}\right)S_{t_{k}^{n}\wedge t,t_{k+1}^{n}\wedge t}^{⊗2}\right),$$

where the convergence is uniform in t∈[0,T]. For every *n* and *k*, we have, by Taylor expansion,

(B.4)
$$\begin{array}{c} \begin{array}{cc} & Y_{t_{k}^{n}}f{\left(S\right)}_{t_{k}^{n}\wedge t,t_{k+1}^{n}\wedge t}\\ & \;=Y_{t_{k}^{n}}Df\left(S_{t_{k}^{n}}\right)S_{t_{k}^{n}\wedge t,t_{k+1}^{n}\wedge t}+\frac{1}{2}Y_{t_{k}^{n}}D^{2}f\left(S_{t_{k}^{n}}\right)S_{t_{k}^{n}\wedge t,t_{k+1}^{n}\wedge t}^{⊗2}+Y_{t_{k}^{n}}R_{t_{k}^{n}\wedge t,t_{k+1}^{n}\wedge t}, \end{array} \end{array}$$

where

$$R_{u,v}:=\int_{0}^{1}\int_{0}^{1}\left(D^{2}f\left(S_{u}+r_{1}r_{2}S_{u,v}\right)-D^{2}f\left(S_{u}\right)\right)S_{u,v}^{⊗2}\;r_{1}\;dr_{2}\;dr_{1}.$$

Since $f\in C^{p+\varepsilon }$, we have that $|R_{u,v}\left|≲\right|S_{u,v}{|}^{p+\varepsilon }$, from which we see that *R* has finite p/(p+ε)‐variation. Since p/(p+ε)<1, it follows that

$$\lim_{n\to \infty }\sum_{k=0}^{N_{n}-1}Y_{t_{k}^{n}}R_{t_{k}^{n}\wedge t,t_{k+1}^{n}\wedge t}=0,$$

where the convergence is uniform in t∈[0,T]. Thus, taking $\lim_{n\to \infty }\sum_{k=0}^{N_{n}-1}$ in Equation (B.4) and substituting into Equation (B.3), we deduce the result.□
